# Provenance and distribution of potentially toxic elements (PTEs) in stream sediments from the eastern Hg-district of Mt. Amiata (central Italy)

**DOI:** 10.1007/s10653-025-02434-8

**Published:** 2025-03-20

**Authors:** Federica Meloni, Enrico Dinelli, Jacopo Cabassi, Barbara Nisi, Giordano Montegrossi, Daniele Rappuoli, Orlando Vaselli

**Affiliations:** 1 Department of Earth Sciences, Via G. Pira, 4, 50121 Florence, Italy; 2 CNR-IGG, Institute of Geosciences & Earth Resources, Via G. Pira, 4, 50121 Florence, Italy; 3 Department of Biological, Geological and Environmental Sciences, P.za Porta S. Donato, 1, 40126 Bologna, Italy; 4 INGV, Istituto Nazionale di Geofisica e Vulcanologia, Via di Vigna Murata, 605, 00143 Rome, Italy; 5 INGV, Istituto Nazionale di Geofisica e Vulcanologia, Viale Carlo Berti Pichat, 6/2, 40127 Bologna, Italy; 6 Unione dei Comuni Amiata Val d’Orcia, Unità di Bonifica, Via Grossetana 209, 53025 Siena, Piancastagnaio Italy; 7 Parco Museo Minerario di Abbadia San Salvatore, Via Suor Gemma, Abbadia San Salvatore 1, 53021 Siena, Italy

**Keywords:** Central Italy, Mt. Amiata, Stream sediments, Mercury, Potentially toxic elements, Geochemical baseline

## Abstract

Geochemical analysis of Potentially Toxic Elements (PTEs) in stream sediments is essential for understanding environmental impacts in areas with complex geology and mining activities. This study focuses on stream sediments from the eastern sector of the Mt. Amiata Hg-district (central Italy) to define the background values of As, Sb, Cr, V, Co, Cu, Ni, and Hg, the latter being speciated to assess its bioavailability and mobility for potential environmental risks. The stream sediments are divided into four different groups: (1) Volcanic, (2) Volcanic-dominated, (3) Sedimentary-dominated, and (4) Sedimentary. This subdivision is confirmed by spatial distribution maps. While Cr, Co, V, Cu, and Ni are related to mafic and ultramafic rocks, Hg shows higher concentrations (up to 850 mg/kg) close to the former mining sites and organic-rich areas. In streams draining the Mt. Amiata volcanics and hydrothermal zones, As is up to 311 mg/kg while Sb is uniformly distributed (up to 84 mg/kg), though depleted in volcanic rocks. Consolidated sediment clusters (group 1 and group 2 and group 3 and group 4, respectively) are used to calculate the geochemical background values (crucial for post-extractive land reclamation), which result to overcome the concentrations imposed by the Italian law for Hg in both clusters, As in cluster 1 and Co in cluster 2 (5.2–6.3, 20–24.3, 39.7–48.5, and 26.3–32.2 mg/kg, respectively). In the stream sediments, Hg speciation by thermal desorption highlights the presence of stable Hg forms. Thus, low Hg bioavailability is expected, being also consistent with the soils from the nearby mining areas.

**Supplementary Information:**

The online version contains supplementary material available at 10.1007/s10653-025-02434-8.

## Introduction

Stream sediments are outstanding geochemical tools that apply to investigations on local geology and mapping, exploration of ore deposits and environmental studies. Their composition is affected by the climatic conditions of the catchment area as well as by the feeding geological terrains (Levinson, 1974; Rose et al., 1979; Bonham-Carter & Goodfellow, 1986; Ottesen & Theobald, 1994; Cheng et al., 2007; Buccianti et al., 2008; Carranza et al., 2008; Garret et al., 2008; Farahbakhsh et al., 2019; Salomao et al., 2021; Doherty et al., 2023; Bourdeau et al., 2024). According to Meyer et al. (1979), stream sediments are to be mainly regarded as a compositionally variable matrix than a uniform geological body. Stream sediments indeed belong to a specific drainage catchment, and they are the integrated result of chemical weathering, physical erosion of rocks (including ore deposits) and soils, addition of atmospheric particulate (Ottesen & Theobald, 1994; Najafian et al., 2023 and references therein) and a number of possible sources related to anthropogenic activities.

When target mineralization and/or alteration are exposed to weathering and entrainment in drainage sediments and/or organic material, stream sediments are the most often employed sample media for reconnaissance exploration (Rose et al., 1979). On the other hand, mineralization abundance tends to decrease with increasing distance downstream (Kelepertzis et al., 2010). This is because the geomorphological and climatic regime strongly influences chemical erosion and hydromorphic dispersion (energy is being lost) of mobilized elements in ground and/or surface waters. In a few environments, precipitates and/or organic material may dominate (Doherty et al., 2023), and stream sediments from other non-polluting sources are being diluted (Plumlee, 1999). Organic substrate, iron (Fe)-manganese (Mn)-aluminum (Al) oxides, secondary authigenic phases and drainage sediments are potential factors that may favor the precipitation of chemically mobilized elements. While clastic dispersal is the primary cause influencing the geochemistry of stream sediments, other components affecting their chemical composition include chemically dispersed and/or extremely fine-grained silt, clays, colloids, and/or oxides. According to Feng and Kerrich (1990), some elements may undergo mutual fractionation during weathering, which might hinder their use as surrogate provenance markers.

Main earth alkaline and alkaline elements and other lithophile elements such as Ba, Rb, Sr, and Cs are examples of mutual fractionation since they are extremely mobile during weathering, transport, and sedimentation and are mostly partitioned in the liquid phase. On the other hand, in agreement with Salminen et al. (2005), the release of these elements in river sediments is low as Rb is re-absorbed by clay minerals (e.g. illite), while Sr is mostly contained in lithic fragments and detrital feldspars. Conversely, other elements are relatively recalcitrant to weathering, being hosted in refractory minerals, or adsorbed by clay minerals (Amireh et al., 2022). High field strength elements (Zr, Hf, Nb, Y), several transition elements (e.g. Sc, V, Co, Ni, Ta, Cr), and REEs are examples of immobile elements in stream waters characterized by slightly acid to slightly basic pH values. Aluminum, Si, Fe and Mn tend to be preferentially partitioned in the solid phase or transported as suspended material. Hence, immobile elements are extremely useful since they can serve as provenance proxies (Bhatia & Crook, 1986; Condie, 1993; Holland, 1978; Phillips et al., 2017; Roser & Korsch, 1986, 1988; Taylor & McLennan, 1985).

Since the time of the Etruscans, the southern part of Tuscany (Central Italy) has been known for the presence of polymetallic ore-deposits containing Fe, Zn, Cu, Pb, Sn (e.g. Dini et al., 2024 and references therein). Later on, during the industrialization period (second half of the nineteenth century), elements such as B, Sb, Mn, Ag and Hg were also exploited, although in the early eighties all mining activities in Tuscany shut down (D’Orazio et al., 2020; Nannoni et al., 2022 and reference therein).

Two areas in southern Tuscany stood out from the rest of the region: (1) the “Colline Metallifere” and the Elba Island, characterized by large deposits of pyrite (Dini, 2003), hematite, and Cu–Zn deposits, and (2) the Hg district of Mt. Amiata, classified as the third largest producer of Hg in the world (Rimondi et al., 2019), where more than 100,000 tons of liquid Hg were produced (Segreto, 1991). Most of these ore deposits are linked to the Neogene-Quaternary magmatic-hydrothermal activity (Dini et al., 2024 and references therein). Currently, these two areas are constellated by many abandoned mining sites where tailings, calcines and gangue material interact with meteoric precipitations, causing serious environmental impacts on surface and ground waters (e.g. Meloni et al., 2024a). It is about a couple decades that most these sites are under remediation, although some of them could be considered of relevant importance as source of critical raw materials (e.g. Sb, Cu) according to the new European Legislation (EU 2024/1252).

The European Directive 2000/60/EC, as well as the Italian Legislative Decree 152/2006, provides quality standards for hazardous substances mainly for marine-coastal and transitional water bodies (e.g. lagoons and estuaries). Curiously, stream sediments, though their importance, are apparently not an environmental matrix to pay attention to. The establishment of reasonable post-mining remediation targets in any decommissioned mine requires the knowledge of the residual geochemical signature for areas surrounding mine deposits, known as geochemical background, in pristine areas, or geochemical baseline where anthropogenic activities occurred (Gustavsson et al., 2012; Kelepertzis et al., 2010; Paternie et al., 2023; Protano et al., 1998; Runnels et al., 1998). With this in mind, and due to the presence of mining dumps along the watercourses in the study area that have not been subject to remediation, the sediments in the watercourses cannot be neglected. In the Hg district of Mt. Amiata, some remediation projects were completed (e.g. Siele and Morone mines), or are about to be over (e.g. Abbadia San Salvatore), or to be initiated (e.g. Solforate and Abetina mines). Numerous studies, carried out in this Hg district, have highlighted anomalous Hg concentrations in the river sediments downstream mining activity (e.g. D’Aglio et al., 1966; Protano et al., 1998; Rimondi et al., 2012, 2014a, 2014b, 2019; Colica et al., 2019; Fornasaro et al., 2022).

Mercury is classified as one of the most dangerous contaminants for ecosystems and human health due to its highly toxic effects on living organisms (e.g., Gonzalez-Raymat et al., 2017). In soils and stream sediments, Hg may be present in elemental (Hg^0^), inorganic (Hg_2_^2+^, Hg^2+^) or organic forms (e.g. methyl-Hg). The adverse health effects of Hg depend on its accumulation and speciation in the body (Kim et al., 2016). In its inorganic forms (Hg^2+^), Hg occurs in ore minerals as cinnabar (α-HgS) and metacinnabar (β-HgS), can be regarded as insoluble in aqueous solutions, their solubility constants (logK_ps_) being of − 53 and − 52 mol/L, respectively; (Ariya et al., 2015), and notably less toxic than methyl-Hg or HgCl_2_. Therefore, understanding the presence of Hg species in solid environmental matrices, such as sediments or soils, is of pivotal importance.

This study is aimed at: (1) investigating the source(s) that characterizes the stream sediments in the eastern sector of Mt. Amiata, where most of the mining activity was concentrated; (2) identifying the geogenic or anthropic origin of PTEs in the stream sediments with particular attention to Hg; (3) determining the geochemical background values in the river basins draining the study area and (4) assessing the speciation of Hg in the stream sediments to comprehend whether it can be considered as a potential contaminant of surface waters.

## Study area

**Fig. 1:**
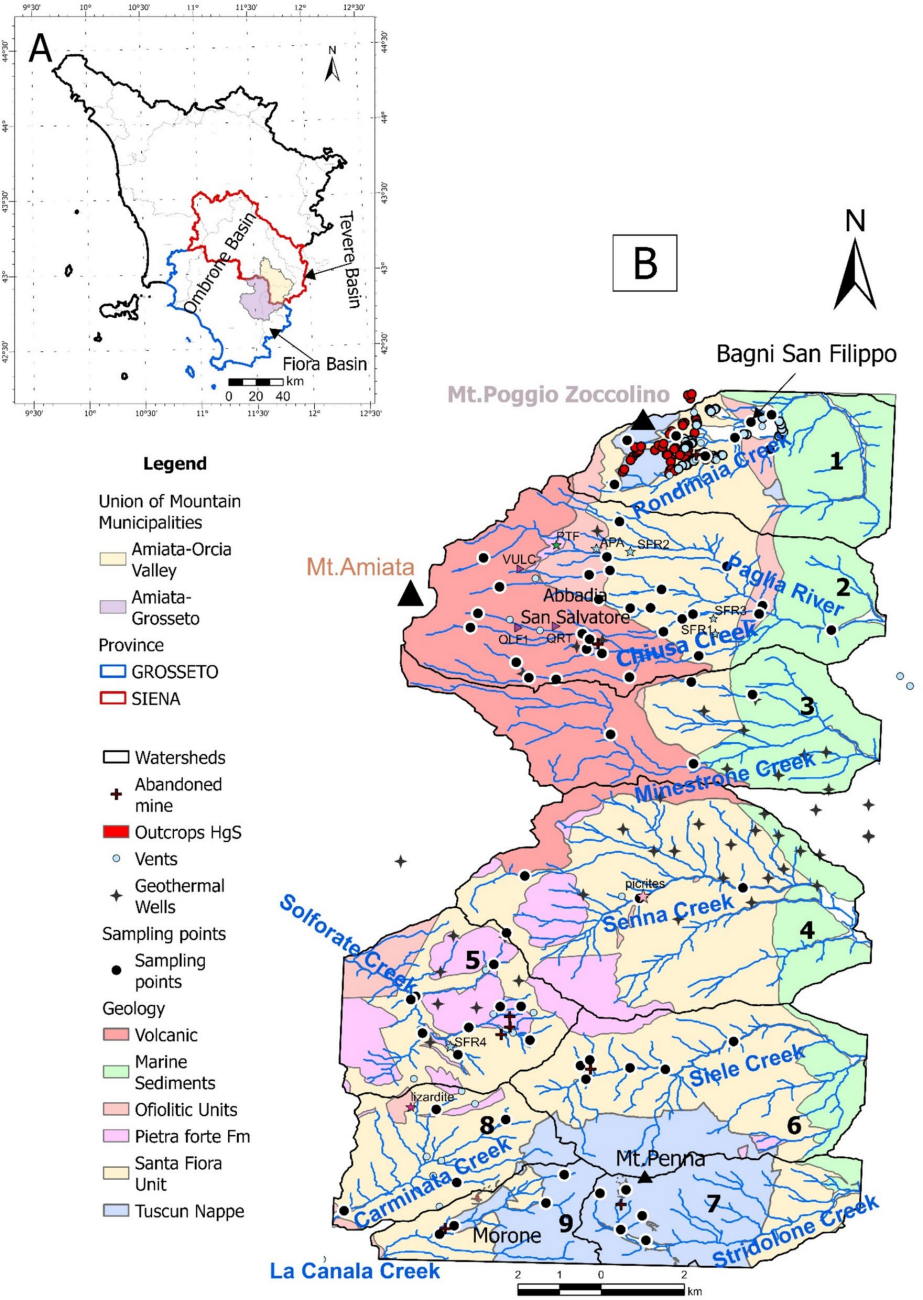
**A** Tuscany Region, Grosseto and Siena Province, Ombrone, Tevere and Fiora Basin. **B** Schematic geological map of the study area. Hg-mineralization, and Hg-abandoned mines, the nine catchments where the main rivers, creeks are reported

The study area is located in the eastern sector of the Mt. Amiata volcano (300–200 ka old, Laurenzi et al., 2015), between the SE part of the Siena Province and the NE part of the Grosseto province, and includes the northernmost portion of three various basins: Fiora, Tevere and Ombrone (Fig. 1A). In the study area, it is possible to subdivide these three basins into nine watersheds, being characterized by eight main creeks (Rondinaia, Minestrone, Senna, Solforate, Siele, Stridolone, Carminata, and La Canala) and one main watercourse (the Paglia river), developing three different hydrological drainage networks from the mountain to the plains: SW-NE, W-E, and NE-SW (Fig. 1B). The surface waters drain eight of the most important former Hg-mines of the Mt. Amiata Hg district (i.e. Abbadia San Salvatore (ASS, hereafter), Siele, Morone, Solforate Rosselli, Solforate Schwarzenberg, Abetina, Petrineri, and Cornacchino, whose activity terminated between 1930 and 1982). In the upper part, the Rondinaia Creek (watershed 1, Fig. 1B) crosses the Tuscan Nappe (Mt. Poggio Zoccolino, 1030 m a.s.l.), a sedimentary cover of the Adria continental paleo-margin (Brogi & Fabbrini, 2010) and the Quaternary travertine plate deposited by the thermal waters present in the area (Chiodini et al., 2020; Fig. 1B).

The northern-central and central part of the area is characterized by the Paglia river (watershed 2 in Fig. 1B), and the Minestrone and Senna creeks (watersheds 3 and 4, respectively, Fig. 1B). They drain the Ligurian Complex, characterized by the Ligurian Units (Lower Cretaceous-Eocene), which consist of the Santa Fiora (External Ligurian Domain) and Ophiolitic Units, and the Mt. Amiata (1780 m a.s.l.) Quaternary volcano (304–231 ky; Laurenzi et al., 2015), the latter being composed by trachytic to olivine latitic lava flows and domes. The Santa Fiora Unit is made up of shales, sandstone, calcarenites, and marly-calcareous bodies (Marroni et al., 2015; Pandeli et al., 2005 and reference therein), while the Ophiolitic Unit includes the Palombini Shales Formation (Lower Cretaceous). Within this formation, remnants of the ancient ocean crust are found, namely “selagiti”, such as pillow lavas, and basaltic dykes (Upper Cretaceous; Pandeli et al., 2005 and reference therein). The Solforate, Siele, Stridolone, Carminata and La Canala creeks (watersheds 5, 6, 7, 8 and 9, respectively, Fig. 1B) flow over the Ligurian Complex and the Tuscan Nappe near Mt. Penna (1050 m a.s.l.).

In the study area, ore deposits, mainly consisting of cinnabar (HgS), realgar (As_4_S_4_), stibnite (Sb_2_S_3_) and pyrite (FeS_2_), are mostly occurring within the Ligurian complex and the Tuscan Nappe (Brogi & Fabbrini, 2010; Forconi, 2011; Losacco, 1960 and references therein), whereas they are scarcely present in the volcanic system. In addition, scattered throughout the study area, thermal waters, dry gas (CO_2_-rich) vents, and fossil and active travertine deposits (i.e. Bagni di San Filippo, Fig. 1B) occur (Chiodini et al., 2020; Tassi et al., 2009; Vaselli et al., 2012). About 54% of the study area is covered by forests, while ca. 27% are devoted to agricultural areas and grazing lands whereas urban and industrial areas and rock outcrops occupy the remaining 19% of the investigated territory.

## Materials and analytical methods

### Sampling strategy and analysis methods

Between January and December 2022, sixty-three stream sediments from the nine watersheds were collected (Fig. 1B) with a plastic shovel. These samples were integrated with five stream sediments from the database created by Fornasaro et al. (2022) (i.e. MSIE 05, MSIE11, MSIE 16 SIE189, SIEBR 2 in watershed 6) for the Siele and Paglia Rivers, and six sediments from Nannoni, A (personal communication) (i.e. 9163A22, 9171A22, 9080A22, 9081A22, 9083A22, and 9211A22 in watershed 5), for a total of seventy-four stream sediments.

The sampled material was stored in 1L polyethylene containers before being transferred to the laboratory. For each sample, the geographical coordinates (UTM WGS84—32N) were acquired with a Garmin GPS with an average error of 3 m. Moreover, six rocks from the main outcrops of the study area were sampled, as follows: three rocks from the Ofiolitic Unit (Lizardite, Filone and Altered Filone), a sedimentary rock from the Santa Fiora Unit (SFR4), a sandstone sample from the Pietraforte Fm. (PTF) and a Triassic anhydrite pertaining to the Burano Fm. (Burano). In addition, the rocks reported by Meloni et al., (2023a, 2023b), and four main volcanic lithologies in the study area (Olivine Latitic lava flow, Pianello Fm., Bellaria Fm., and Quaranta Fm.; Conticelli et al., 2015) were also included in this study. Characteristics and main mineralogical phases (Meloni et al., 2023b) of the investigated rock suite were analyzed using transmitted light microscopy. The stream sediments were oven-dried at 30 °C to minimize as much as possible the release of gaseous mercury, if present. Each sample was then sieved at 2 mm according to the protocol adopted by the Environmental Protection Agency of Tuscany (ARPAT) and described in Meloni et al. (2022). The < 2 mm fraction was then ground by a planetary with agate mortars and balls for 30 min.

The mineralogical composition was semi-quantitatively determined using X-Ray Diffraction (XRD) with Cu-K α radiation. The analysis was conducted with a D8 “Da Vinci” Diffractometer (Bruker) at the CRIST (Centro di Servizi di Cristallografia Strutturale) Laboratories (University of Florence).

The concentration of major oxides (SiO_2_, TiO_2_, Al_2_O_3_, Fe_2_O_3_, MnO, MgO, CaO, Na_2_O, K_2_O, P_2_O_5_) and trace elements (Nb, Zr, Y, Sr, Rb, Ce, Ba, La, Ni, Cr, Co, S, Cu, Zn, Pb, As, V, Cl, Nd) was determined in the rocks and stream sediments using a X-Ray Fluorescence spectroscopy (XRF) on pressed powder pellets using a Panalytical Axios 4000 equipped with a Rh tube at the Department of Biological, Geological, and Environmental Sciences (University of Bologna). The overall accuracy was within the reproducibility range, as detailed in Lancianese and Dinelli (2015), due to a calibration curve constructed using certified reference materials. Reproducibility for major elements was generally better than 5%, while trace elements showed an average reproducibility < 10%. Loss on ignition (LOI) was gravimetrically assessed by heating the samples for 2 h at 950 °C (LOI%). This measure represents the weight percentage of volatile substances, such as structural water in the mineral lattices, gases, inorganic carbon, and organic matter (OM).

OM was also determined via gravimetry. About 0.25 g of stream sediment were placed in fiber quartz crucibles and then heated at 550 °C for 1h. The Hg concentrations were measured following the EPA 7473 (2007) method, using DMA 80 (Milestone), at the accredited Laboratories of C.S.A. Group Ltd. (Rimini, Italy) with LoQ (Limit of Quantification) of 0.005 mg/kg. Arsenic, Sb, Co, Ni, Cu, Cr_tot_, and V were analyzed in the same laboratory after aqua regia digestion, following the EPA 3051A + 6010D method, using ICP-AES (Agilent 720 ES), with LoQ 1 mg/kg for As and Sb, and 0.5 mg/kg for the other elements, respectively. Three replicates were performed for each sample and the error was < 10%. The pH was determined in a soil–water suspension with a 1:5 w/v ratio, following the IRSA-CNR (1985) method, at the Department of Earth Sciences of Florence.

Mercury in the stream sediment leachates was also analyzed by the Laboratories of C.S.A. Group Ltd. (Rimini, Italy) using methods UNI 10802 (2013), UNI EN 12457–2 (2004), UNI EN 16191 (2012), and UNI EN ISO 17294–2 (2016), with LoQ of 0.1 µg/L. Arsenic and Sb in leachates were measured using ICP-MS (Agilent 7800) at the Department of Earth Sciences of Florence, after 24 h of interaction between soil and MilliQ water (ratio 1:10) under agitation. The LoQ was 0.1 µg/L.

### Thermal desorption and Hg speciation

Mercury speciation by thermal desorption (TD) technique was carried out in those stream sediments where the Hg concentration was > 5 mg/kg, this limit being able to provide reliable speciation results (Meloni et al., 2024b). TD implies the use of Lumex-915 + coupled with Pyro-915 + , following the method proposed by Meloni et al. (2024b). The heating ramp with three different steps was selected to warm-up the instrument: (1) first step at 28.8 °C min^−1^ for 140 s; (2) second step at 40.8 °C min^−1^ for 340 s and (3) third step at 49.8 °C min^−1^ for 360 s. The temperature at the beginning of the heating ramp was set up at 36 °C and at the end of each run the temperature reached 635 °C. Hg-free air was used as carrier gas with a flow rate of ca. 3 L min^−1^. The analytical curve was constructed with four different aliquots (5, 10, 20 and 30 mg) of the NIST 2711A standard (mass fraction of Hg = 7.42 ± 0.18 mg/kg). Pure Hg standards (Meloni et al., 2024b; Rumayor et al., 2013) were used for the interpretation of the thermo-desorption profiles (TDP). Additionally, the total Hg concentrations and the corresponding proportion of the species (area in %) were determined using the Biester and Scholz (1996) method by integrating the area subtended by each peak.

### Statistical analysis and determination of geochemical baseline and background

R and RStudio (R Core Team, 2021) were used to statistically analyze the chemical data and compute summary exploratory statistics of the metal concentrations in soils. Chemical data below LoQ were substituted with two-thirds of the LoQ for statistical computations (Gozzi, 2020; Gozzi et al., 2021). Using ArcGis-Pro 3.0, dot-distribution maps of stream sediments were produced. The Spearman correlation matrix was used for the correlation analysis among the stream sediment PTEs since it is relatively robust against data outliers (Reimann et al., 2017).

Although river sediments are not regulated under the Italian and European Environmental Legislation, defining natural geochemical background values or threshold values can help authorities to understand the source of potential environmental contaminations. In this regard, to calculate the background values, the ProUCL 5.2 software (Singh & Maichle, 2015) was used following the guidelines provided by Meloni et al. (2023b) and expressed as an interval of concentrations (for more details see Meloni et al., 2023b). According to many authors (e.g. Cave et al., 2012; Johnson et al., 2012; Meloni et al., 2023b; Reiman & de Caritat, 2017), when the distribution only follows the log-normal distribution, the graphical methods (Cumulative Probability Diagram CP-plot) was used to verify the value obtained by ProUCL. The background values can be recognized in correspondence with the breaking slope present in the CP-plot. As suggested by SNPA (2017), the geochemical background value of river sediments will only be calculated for those metals (i.e. As, Sb, Cr, Co, Cu, Ni and V) analyzed after extraction in aqua regia and Hg analyzed by EPA 7473 method.

## Results

### Mineralogical composition

Tables S1.1 and S1.2 (Supplementary Materials S1) list the mineralogical features for the investigated rocks and stream sediments, respectively. Supplementary Material S2 summarizes the main petrographic characteristics of the studied rocks. The XRD analysis of the six rocks sampled for this study highlighted that PTF mainly consists of calcite, quartz, and dolomite, with minor contents of plagioclase and phyllosilicate (e.g. mica, illite, chlorite, kaolinite). SFR4 has calcite, quartz, phyllosilicate and minor plagioclase. The Burano rock is characterized by anhydrite and trace of calcite, whereas lizardite has tiny talc veins, and a high amount of serpentine crystals. The Filone sample is dominated by phyllosilicates (chlorite, biotite) and pyroxene. Analcime is a minor component, while K-feldspar is present as a trace component. Conversely, the main components of Altered Filone are calcite, phyllosilicates (chlorite and biotite) and antigorite (serpentine group), with few quartz, plagioclase and analcime crystals. According to Tangari et al. (2020), analcime is a typical hydrothermal alteration in volcanic materials. The XRD analysis of other sedimentary rocks (SFR1, 2, 3) and volcanic rocks (QRT, VULC and OLF1) are reported in Meloni et al. (2023b).

The microscope observations support the XRD analyses, except for analcime and kaolinite, illite and dolomite, which were not recorded, likely due to the fact that their contents is < 4%. According to Faraone and Stoppa (1990) and Stoppa et al. (2014), the Filone sample can be classified as an alkaline lamprophyre. The main components are clinopyroxene, biotite, muscovite, chlorite and, to a lesser extent, serpentine, due to the alteration of olivine (present as a relict) and biotite (Fig. 12B, C, Supplementary Material S2). The formation of serpentine and thus the alteration of olivine and biotite, as well as the presence of calcite, are likely associated with hydrothermal metamorphism. As accessory minerals, apatite and Fe-oxy-hydroxides are present. In this rock, serpentine was not recorded by XRD analysis, whilst it is found in the altered sample (Altered Filone) as antigorite. Chlorite and serpentine content increases when compared to that occurring in the unaltered rock. The microscope observations of the three volcanic rocks (QRT, VULC and OLF1) confirmed the XRD analysis reported in Meloni et al. (2023b).

In the stream sediments, K-feldspar and plagioclase are minor minerals, whereas quartz and phyllosilicates (clay minerals and mica) are the dominant minerals. K-feldspar is the juvenile mineral in the stream sediments collected from the volcanic lithology. The dominant component in the stream sediments draining the sedimentary lithologies is calcite. Gypsum occurs in Salto 1, Salto 3, STA41 and STA15. Ankerite is only recorded in STA16, while hematite sporadically occurs as trace.

### Descriptive statistics of major and trace elements in rocks and stream sediments

**Fig. 2:**
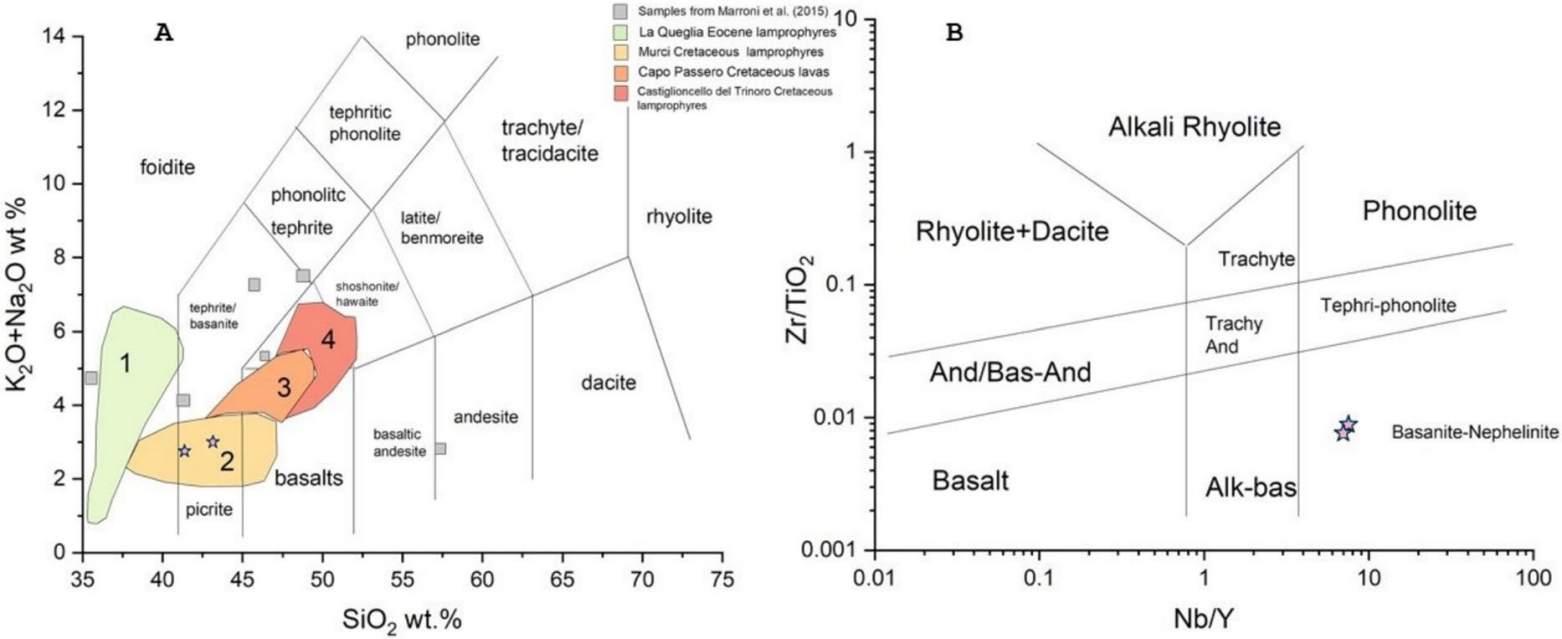
**A** The Total Alkali vs. SiO_2_ (TAS) diagram for Filone and Altered Filone (modified after Faraone & Stoppa, 1990 and Stoppa et al., 2014); **B** Nb/Y vs Zr/TiO_2_ classification diagram, in logarithmic scale (Pearce, 1996), for the two most basic rocks

**Fig. 3:**
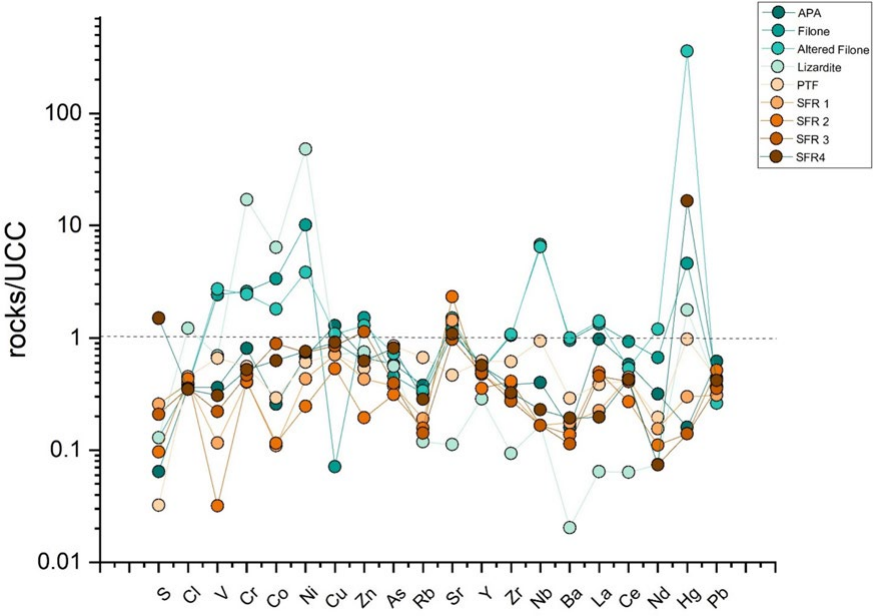
Spider diagram of the rocks analyzed by XRF. Concentrations are normalized to UCC (Upper Continental Crust: Rudnick & Gao, 2003. Elements are ordered according to increasing atomic number

The concentrations of major (expressed as oxides in wt.%) and trace elements (in mg/kg) of the rocks SFR1, SFR2, SFR3, SFR4 and APA, with the exception of the Burano sample, and of those reported in Meloni et al. (2023b) are listed in Table 1. SiO_2_ is the most abundant major oxide, except for SFR2, where CaO prevails (44.8wt %). CaO in the other rocks is the second constituent except for Filone, Altered Filone, and Lizardite, where MgO shows the highest content (38.3%). LOI is ranging from 10.0 to 40.0 wt.%. According to Marroni et al., (2015 and reference therein) and based on the Total Alkali Silica (TAS) diagram (Le Maitre, 2002), the lamprophyres from the Valle del Senna Creek vary from foidite to tephrite, trachybasalt, and andesite. Therefore, by plotting the results of the two rocks (Filone and Altered Filone) on the TAS diagram from Marroni et al. (2015) (Fig. 2A), they can be classified as basanite/picrite. Further confirmation of the mantle source of this rock is provided by the Nb/Y vs. Zr/TiO_2_ binary diagrams (Pearce, 1996) (Fig. 2B), where the two rocks fall within the basanite-nephelinite field. These two rocks have SiO_2_ and MgO concentrations within the range reported by Marroni et al. (2015), i.e. 52.7–27.3 wt.%, and 2.0–10.4 wt.%, respectively. Figure 3 shows a spider diagram normalized to the Upper Continental Crust (UCC, Rudnick & Gao, 2003). While serpentinite is also enriched in Cl, the two basanite/picrite rocks are enriched in La, Nb, V, and Zn. In addition, the altered basanite/picrite is also enriched in Cu, and Nd. It should be noted that these samples are characterized by a Hg content of 0.23 and 18.1 mg/kg, respectively.

**Table 1:** Major (SiO_2_, TiO_2_, Al_2_O_3_, Fe_2_O_3_, MnO, MgO, CaO, Na_2_O, K_2_O, P_2_O_5_ and LOI) expressed as wt.%, and trace elements (mg/kg) for the rock samples analyzed with XRF. Arsenic and Sb, and Hg (mg/kg) were analyzed after aqua regia digestion and after method EPA 7473, respectively

		APA	Filone	Altered Filone	Lizardite	PTF	SFR 1	SFR 2	SFR 3	SFR4
XRF	SiO_2_	27.3	37.2	37.9	37.2	51.7	24	10.7	39.1	41.3
	TiO_2_	0.2	2.3	2.7	0.1	0.4	0.1	0.1	0.1	0.2
	Al_2_O_3_	6	9.5	9.3	2	8.2	2.7	1.7	5.3	5.2
	Fe_2_O_3_	4.5	9.9	9.2	7.5	3	3.1	1.6	10.4	6.2
	MnO	0.1	0.1	0.1	0.1	0.1	0.2	0.1	0.3	0.2
	MgO	0.9	23.6	14.1	38.3	4.3	1.4	0.4	2.5	1.7
	CaO	30.2	4.3	11.2	0.1	15.2	34.2	44.9	20	24
	Na_2_O	0.4	0.8	0.7	0.1	0.5	0.2	0.2	0.1	0.3
	K_2_O	1	1.7	2	0	1.6	0.5	0.4	0.2	0.6
	P_2_O_5_	0.1	0.5	0.6	0	0.1	0.1	0.1	0.1	0.1
	LOI	29.2	10	12	14.3	14.7	33.4	40	21.9	20.2
	S	40	80	160	80	20	160	60	130	930
	Cl	135	140	145	453	166	147	160	133	129
	V	35	234	264	67	64	11	3	22	30
	Cr	74	238	224	1564	51	37	38	43	48
	Co	5	58	31	111	5	2	2	15	11
	Ni	32	476	180	2252	29	20	12	35	36
	Cu	36	2	31	20	20	20	15	24	26
	Zn	45	101	87	50	36	29	13	77	42
	As	3	2	4	3	4	2	2	2	4
	Rb	32	27	28	10	56	16	13	12	24
	Sr	417	387	477	36	150	459	740	311	350
	Y	12	11	11	6	13	10	8	10	12
	Zr	75	204	207	18	119	60	79	53	63
	Nb	5	81	77	2	11	2	2	2	3
	Ba	99	597	626	13	181	110	86	71	121
	La	30	41	44	2	12	7	15	14	6
	Ce	37	59	34	4	26	28	17	26	27
	Nd	9	18	32	2	5	4	3	2	2
	Pb	11	6	5	6	7	5	9	6	7
EPA 7473	Hg	0.008	0.23	18.01	0.088	0.049	0.015	0.007	0.007	0.83
Aqua regia	As	1	1	3	1	2	1	1	1	2
	Sb	4	< 1	< 1	< 1	2	1	< 1	3	< 1

According to De Castro (1914), cinnabar (HgS) was found in the calcareous veins or at contact with the serpentinite itself (from the mid-nineteenth century to the early 2000s, the green rocks within the Senna River, here classified as basanite/picrite, were considered serpentinites). For this reason, in 1846, the Modigliani Society carried out investigations and the first Hg-mine, Casa di Paolo, opened (De Castro, 1914). The rocks in the Santa Fiora Unit are enriched in Sr (up to 740 mg/kg). SFR3 differs with respect to SFR2 due to its enrichment in Zn (up to 77 mg/kg), while SFR4 distinguishes from the other rocks for S and Hg, whose concentrations are up to 930 mg/kg and 0.83 mg/kg, respectively. The main statistics (minimum, maximum, mean, median, standard deviation (SD), skewness and 95 percentile) of major oxides, LOI and trace elements (including As, Sb and Hg) of stream sediments are listed in Table 2.

**Table 2:** Minimum (min), maximum (max), mean, median, standard deviation (SD), Skewness, 95% percentile (95%itle) of SiO_2_, TiO_2_, Al_2_O_3_, Fe_2_O_3_, MnO, MgO, CaO, Na_2_O, K_2_O, P_2_O_5_, LOI, S, V, Cr, Co, Ni, Cu, As, Rb, Sr (analyzed by XRF) and Hg (analyzed by EPA 7473 method) in the stream sediments, and mean of the same elements from FOREGS Database (Salminen et al., 2005)

	Min	max	Mean	median	SD	Skewness	95%ile	mean-FOREGS
SiO_2_	34.3	62.6	50.1	49.4	6.5	−0.187	60.1	60.1
TiO_2_	0.4	1.2	0.7	0.8	0.2	−0.208	1	0.68
Al_2_O_3_	11.3	27.3	17.7	18.1	2.8	−0.255	21.3	10.2
Fe_2_O_3_	2.9	11.6	6.8	6.9	1.9	0.31	10.4	4.07
MnO	0	0.6	0.2	0.1	0.1	1.825	0.4	0.112
MgO	0.7	4.7	1.8	1.7	0.7	1.01	2.5	1.77
CaO	1.1	18.6	5.3	4.2	3.9	1.373	12.8	5.81
Na_2_O	0.2	1.6	0.7	0.6	0.4	0.984	1.4	1.14
K_2_O	1.2	7.6	3.4	3.2	1.7	0.932	6.8	2.08
P_2_O_5_	0.1	0.5	0.2	0.1	0.1	3.073	0.2	0.14
LOI	2.7	24.6	12.7	12.7	5.4	0.062	21.7	–
S	50	19,760	1014	410	2490	6.299	3992	–
V	66.8	234	138.6	144.6	39.5	−0.096	193.1	68.3
Cr	36.5	172	98.5	105.9	34.6	−0.194	148.5	92.8
Co	6	54	23.7	23.5	11	0.607	44	11.2
Ni	18	92	52.7	57.2	18.7	−0.399	79.4	35.2
Cu	12.6	69	42.9	47.1	16.1	−0.565	66.4	22.1
As	4.9	68.6	24	13.9	19	0.996	60.8	10.1
Rb	42.6	376	155.8	127.8	101.9	0.924	369	77.9
Sr	131.6	500	289.4	271.4	98.9	0.345	453.2	171
Hg	0.088	850	50.6	2.4	146.6	4.2	260	0.081

All concentrations of the trace metals are expressed in mg/kg, while those of LOI and the major oxides are in wt.%

The stream sediment statistics for pH, As, Sb, Cr_tot_, Cu, Co, V, and Ni (in mg/kg), analyzed after aqua regia digestion, and organic matter (OM, in wt.%) are listed in Table 3. Additionally, the mean values of major oxides and trace elements analyzed by XRF and after aqua regia dissolution of stream sediments from the FOREGS-Geochemical Atlas of Europe (http://weppi.gtk.fi/publ/foregsatlas/) are also reported for comparison in Tables 2 and 3, respectively. The whole dataset, including the geographic coordinates, is listed in Table S3.1, S3.2 and S3.3 (Supplementary Materials S3).

**Table 3:** Minimum (min), maximum (max), mean, median, standard deviation (SD), Skewness, 95% percentile (95%ile) of pH, As, Sb, Co, Ni, Cu, Cr, V and organic matter (OM) in the stream sediments analyzed by aqua regia extraction, and mean of the same elements from FOREGS database

	min	max	mean	median	SD	Skewness	95%ile	mean-FOREGS
pH	4.2	8.9	7.6	7.7	0.6	− 2.8	8.2	–
As	1.5	84	13.9	5.8	36.8	7.3	35.5	9.5
Co	2.1	46.7	7.1	4.8	11.8	5.5	10.5	10.3
Ni	3.9	95.9	13.0	11.7	8.2	1.5	26.9	28.6
Cu	2.7	84.1	23.7	21.2	14.3	1.5	48.1	19
Cr	3.9	80	28.6	27.0	18.6	0.6	60.9	31
Sb	< 1	83.8	28.9	28.1	18.3	1.1	59.3	–
V	7.5	99.2	28.5	22.9	18.1	1.8	70.7	33
OM	1.5	28.5	6.2	5.1	4.4	2.9	15.1	–

All concentrations, except pH and OM (%), are in mg/kg

**Fig. 4:**
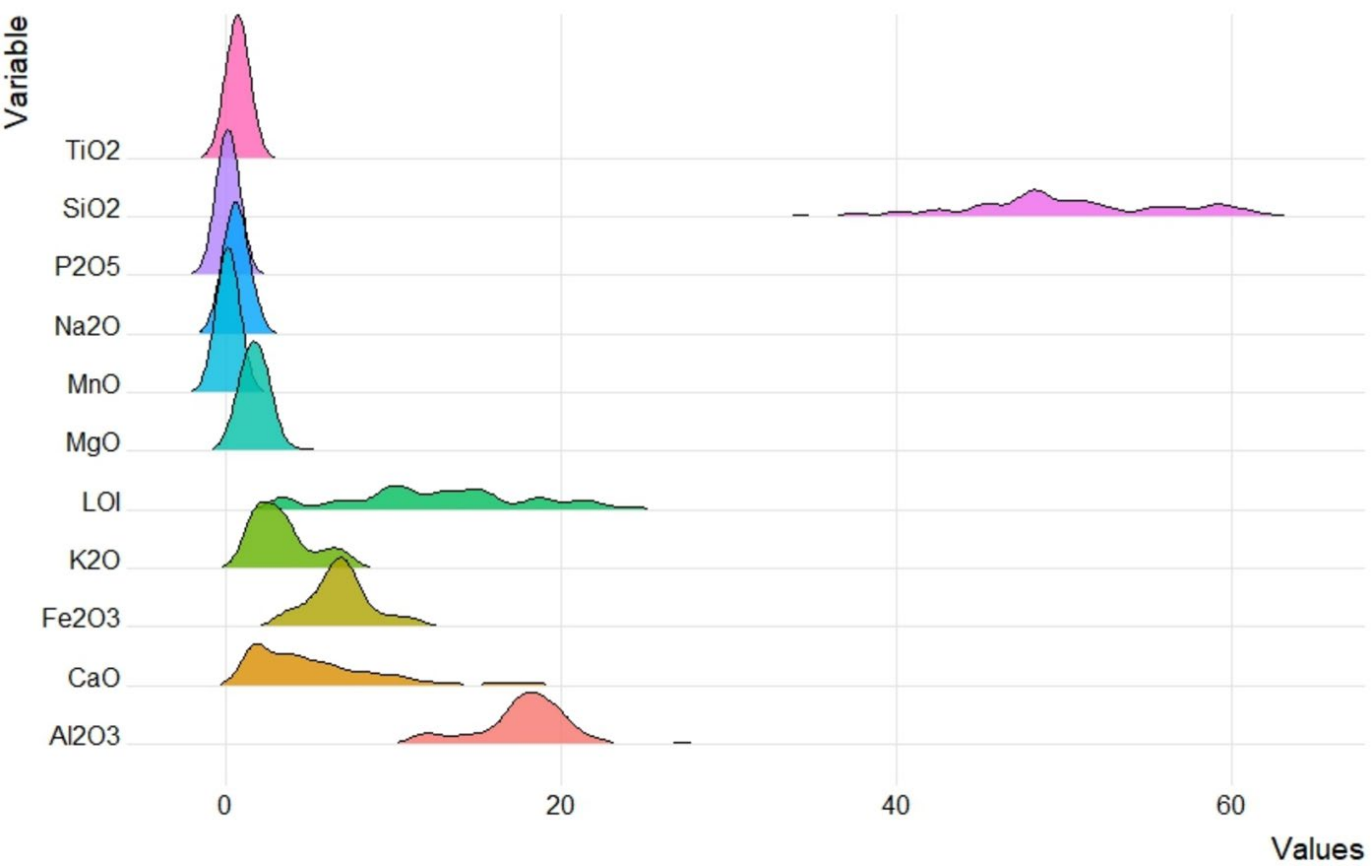
Ridge plots of major elements and LOI (in wt.%) of the stream sediments

In the study area, SiO_2_, Al_2_O_3_, LOI, Fe_2_O_3_ and CaO are the most abundant components of the sediments, with a mean value of 50.1, 17.7, 12.7 and 5.3 wt.% (Table 2 and Fig. 4), respectively. This is in good agreement with the XRD analysis, where quartz, phyllosilicates, calcite and K-feldspar are the main mineralogical phases. By comparing the average concentrations of major and trace elements in stream sediments with those of the main European rivers, the concentrations of Al_2_O_3_, Fe_2_O_3_, K_2_O, As, Co, Cr, Cu, Ni, Rb, Sr, and V are higher than those reported by FOREGS (Salminen et al., 2005). In particular, Rb and V differ from the European averages by, at least, one order of magnitude. Mercury had largely variable concentrations (from 0.088 to 850 mg/kg), with the mean values higher with respect to those reported in the FOREGS database for the stream sediments (Salminen et al., 2005).

**Fig. 5:**
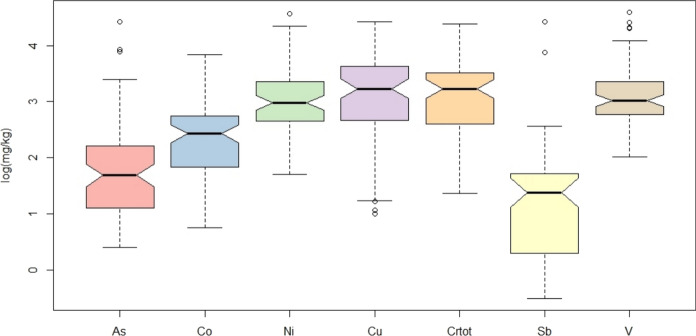
Boxplots of trace elements determined after aqua regia digestion (values in logarithmic scale)

The pH in the stream sediments (Table 3) is tendentially neutral or alkaline. Only the sample STA41 (watershed 1) shows an acidic pH (4.2). Arsenic and Sb have largely variable concentrations since they span of two orders of magnitude (from 2 to 311 mg/kg, and from < 1 to 83.8 mg/kg, respectively) (Fig. 5). Differently, Co, Cr, Cu, and Ni, analyzed after aqua regia extraction, are characterized by concentrations varying within one order of magnitude (from 2.6 to 46.7 mg/kg, from 5.5 to 80.0 mg/kg, from 2.7 to 84.2 mg/kg, and from 3.9 to 95.9 mg/kg, respectively), while those of V are oscillating between 7.5 and 99.2 mg/kg. By comparing the mean concentrations with those from the FOREGS database, all the elements in the stream sediments from the study area, except Sb and Cr, result to be enriched. Moreover, OM evidences a heterogeneous distribution, with a minimum and maximum content of 1.5% and 28.5%, respectively (Table 3).

### Hg leachates in stream sediments

Table 4 illustrates the results of the Hg leachates in the stream sediments. Most samples have Hg concentration below the instrumental detection limit (0.1 µg/L), and only two samples (Salto 4 and STA 47, in watersheds 5 and 7 respectively) show a Hg value higher than the maximum content allowed by the Italian law (1 µg/L: Legislative Decree 31/2001) (Table 4).

**Table 4:** Concentration of Hg (µg/L) in the stream sediment leachates

Sample	Hg μg/L	Sample	Hg μg/L	Sample	Hg μg/L	Sample	Hg μg/L	Sample	Hg μg/L
STA1	0.2	STA19	0.2	STA37	b.d.l	MSIE-16	b.d.l	9083A22	b.d.l
STA2	0.3	STA20	0.1	STA38	b.d.l	MSIE-04	n.d	9084A22	b.d.l
STA3	0.1	STA21	b.d.l	STA39	b.d.l	sie 189	b.d.l	9211A22	b.d.l
STA4	0.3	STA22	b.d.l	STA40	b.d.l	SIE Br2	b.d.l	9163A22	b.d.l
STA5	0.1	STA23	b.d.l	STA41	b.d.l	MSIE 05	n.d	9189A22	b.d.l
STA6	b.d.l	STA24	b.d.l	STA42	b.d.l	Msie 11	b.d.l		
STA7	0.1	STA25	b.d.l	STA43	b.d.l	Salto 1	b.d.l		
STA8	0.2	STA26	b.d.l	STA44	0.2	Salto 2	b.d.l		
STA9	0.1	STA27	b.d.l	STA45	b.d.l	Salto 3	b.d.l		
STA10	0.1	STA28	b.d.l	STA46	0.4	Salto 4	2.5		
STA11	0.1	STA29	b.d.l	STA47	1.6	Green Lake	b.d.l		
STA12	0.2	STA30	b.d.l	STA48	b.d.l	GIT0	n.d		
STA13	0.1	STA31	0.6	STA49	b.d.l	GIT4	n.d		
STA14	0.1	STA32	b.d.l	STA50	b.d.l	GIT12	n.d		
STA15	0.1	STA33	b.d.l	STA51	b.d.l	PAO1	n.d		
STA16	0.2	STA34	b.d.l	STA52	b.d.l	9171A22	b.d.l		
STA17	0.1	STA35	b.d.l	STA53	0.5	9080A22	b.d.l		
STA18	0.1	STA36	b.d.l	STA54	b.d.l	9081A22	b.d.l		

b.d.l, below detection limit (0.1 µg/L); n.d, not determined

### TD Hg in stream sediments

In Table 5, the area subtended by the curve in % for each Hg species and each sample is reported. Mercury is mainly occurring as α-HgS and β-HgS while less is associated with OM.

**Table 5:** Sample ID, % of OM–Hg, % β–HgS, % α–HgS, % HgSO_4_, % HgCl_2_

Sample	OM-Hg	β-HgS	α-HgS	HgSO_4_	Sample	OM-Hg	β-HgS	α-HgS	HgCl_2_
STA1	45.5	39.5	15.0	–	STA52	36.1	11.8	52.1	–
STA2	31.5	–	68.5	–	STA53	–	–	100	–
STA3	41.0	–	59.0	–	STA54	2.6	–	97.4	–
STA4	–	–	100	–	SIE189	2.7	–	97.3	–
STA5	22.1	12.8	65.1	–	MSIE04	19	–	81.0	–
STA8	–	–	100	–	MSIE05	76.4	–	23.6	–
STA16	50.5	–	49.5	–	MSIE11	7.2	91.7	1.1	–
STA20	68.5	10.1	21.4	–	MSIE16	80.8	–	19.2	–
Green Lake	–	–	100	–	SIBR2	22.9	7.0	70.1	–
STA21	3.4	18.5	78.1	–	9171A22	7.33	92.67	–	–
STA22	7.6	–	92.4	–	9081A22	14.7	74.8	10.5	–
STA23	20.4	12.0	67.6	–	9083A22	–	–	100	–
STA24	7.8	2.2	90	–	SALTO1	4.7	49	46.3	–
STA30	–	–	100	–	SALTO2	41.7	12.7	45.6	–
STA44	6.2	6.2	87.6	–	SALTO3	9.6	6.6	83.8	–
STA45	100	–	–	–	SALTO4	0.8	17.2	77.3	4.7
STA46	10.3	–	89.6	–	GIT4	–	–	100	–
STA47	1.07	3.03	54.2	41.7	GIT12	–	–	100	–
STA50	4.1	7.5	88.4	–					

## Discussion

### Sources of major and trace elements in stream sediments

**Fig. 6:**
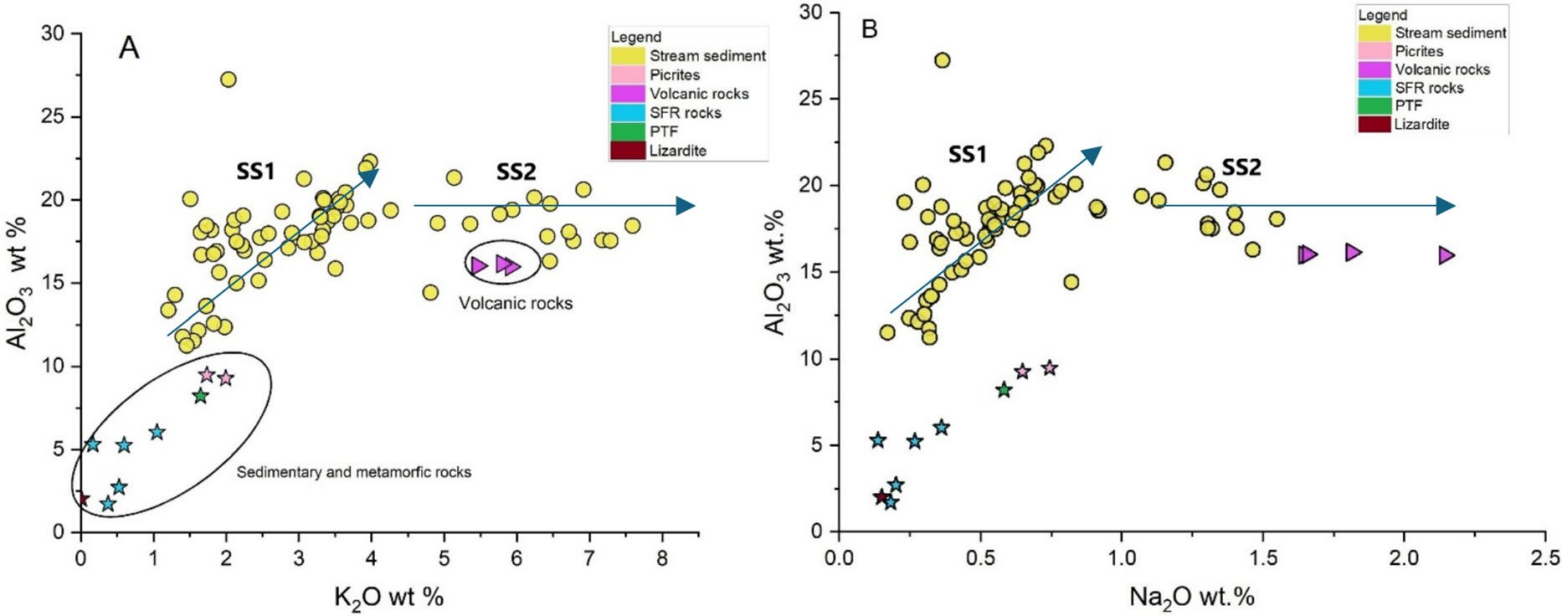
Binary diagrams of K_2_O vs. Al_2_O_3_ (wt.%) (**A**) and Na_2_O vs Al_2_O_3_ (wt.%) (**B**) of stream sediments (yellow circle) and sedimentary (SFR rock, PTF-green star), metamorphic (lizardite-brown star, picrites-blue stars) and volcanic rocks (purple triangle)

According to Rose et al. (1979), stream sediments often represent the mineralogical and geochemical composition of the outcropping rocks in the drainage basin upstream of the sampling sites. The chemical data from the current study are compared to those from the main geological units that constitute the different basins to assess their contribution. The main oxides can be considered markers of grain distribution (Dinelli et al., 2005) or indicators of the percentage of fine material (e.g. Al_2_O_3_, Bianchini et al., 2012). Basically, they capture the distribution of the dominant minerals (Zhao et al., 2019). Two different groups of stream sediments can be recognized in the Al_2_O_3_ vs. K_2_O and Al_2_O_3_ vs. Na_2_O diagrams (Fig. 6A and B, respectively). The first group (SS1) shows a positive correlation between Al_2_O_3_ vs. K_2_O and Al_2_O_3_ vs. Na_2_O, whereas the second group (SS2) is characterized by an increase in K_2_O and Na_2_O at almost constant Al_2_O_3_ concentration. SS1 refers to those stream sediments that have a higher Al_2_O_3_ concentration compared to the sedimentary and metamorphic (blue and violet stars, respectively) rocks. In most cases, the median of the volcanic rocks (purple triangles) is likely associated with Al-bearing mineralogical phases such as clay minerals (Bianchini et al., 2012; Cox et al., 1995; Hossain et al., 2017) or related to different alteration processes that favor Al_2_O_3_ over Na_2_O. SS2 is represented by stream sediments that approach the concentrations of K_2_O and Na_2_O of the median values of the volcanic rock samples, probably due to the fact that the alteration of K-feldspar is slower than that of Na-plagioclase. The XRD data confirm the presence of K-feldspar and a relatively high amount of plagioclase in the SS2 samples.

**Fig. 7:**
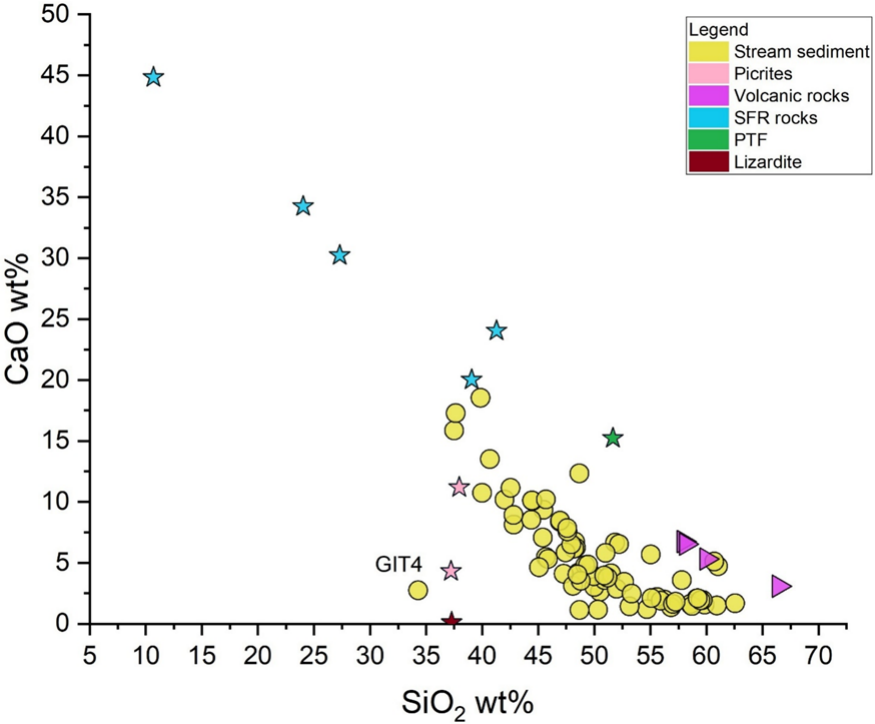
The SiO_2_ vs. CaO (wt.%) binary diagram. Stream sediments (yellow circle) and sedimentary (SFR Rock and PTF in green stars), metamorphic (lizardite and picrites in brown and blue star, respectively) and volcanic rocks (purple triangle) are also reported

The binary diagram SiO_2_ vs. CaO (Fig. 7) evidences the negative correlation between the two parameters, which is due to the increasing abundance of carbonate minerals (mostly calcite) over the silicate fraction. Sample GIT 4, collected from the Fosso della Chiusa creek (Fig. 1B), is fed by waters discharging from the mining galleries of the former mining areas of ASS and rich in Fe-Al-oxy-hydroxides (Lazzaroni et al., 2022; Vaselli et al., 2021). This sample diverts from the main trend, showing lower concentrations of SiO_2_ and CaO with respect to the other stream sediments. The presence of Fe is supported by XRD analysis, where goethite was recorded.

**Fig. 8:**
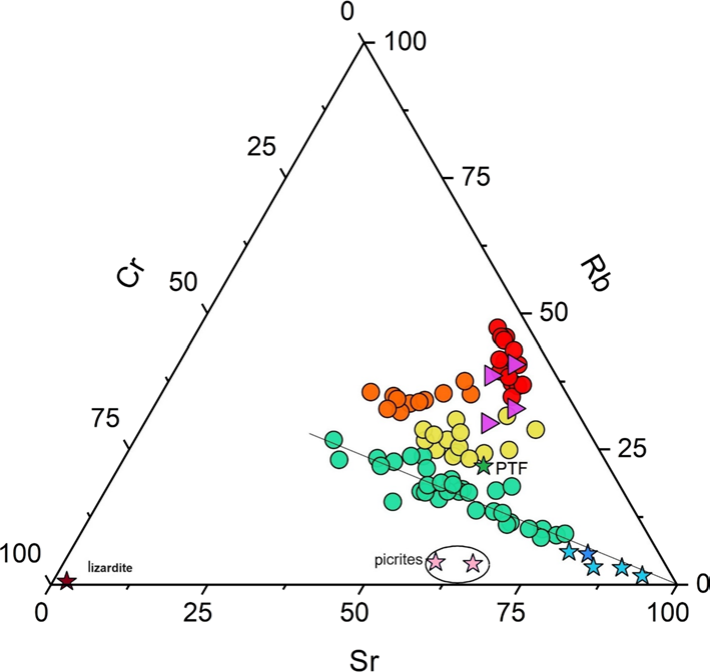
The Sr-Rb-Cr ternary diagrams (mg/kg). Red circles: stream sediments of volcanic provenance (Volcanic); orange circles: mixing between a sedimentary and a dominating volcanic component (Volcanic-dominated); yellow circles: mixing processes between volcanic and sedimentary components where stream sediments are prevalently draining sedimentary rocks (Sedimentary-dominated); Green circles: stream sediments of carbonate provenance (Sedimentary). The violet triangles are the median of the four volcanic lithologies from the study areas. The light blue stars represent the SFR 1, 2, 3 and 4 rocks. The green star represents PTF and the pink stars the picrites

Trace elements can be regarded as more diagnostic tools than the main oxides of the rock source (e.g. Lee, 2018 and references therein). The Sr-Rb-Cr ternary diagram (Fig. 8) allows to better identify the studied stream sediments. Strontium was selected because tends to be enriched in the carbonate component, being a Ca-substitute in carbonate minerals (e.g. calcite). Rubidium is a lithophile element and does not form own minerals, but it occurs in many common minerals since it isomorphogenically replaces potassium in K-minerals (e.g. K-feldspar). Furthermore, since it has a very large ionic radius, it behaves incompatibly and concentrates in the late stage of crystal fractionation of magmas (e.g. Salminen et al., 2005). Consequently, it can be considered a marker for sediments of volcanic origin. Chromium is mainly found in oxides (chromite, magnetite, ilmenite), which are resistant to weathering, while clinopyroxene and tends to be enriched in mafic and ultramafic rocks (Mielke, 1979).

The ternary diagram of Fig. 8, which shows the concentration values of carbonate sedimentary rocks (SFR1, 2, 3, 4 and PTF), lizardite, the two picrites, and the median of the four volcanic facies within the study area, allows to recognize four distinct groups, as follows:

1. Group 1 (Volcanic): stream sediments of volcanic origin (red circles), falling within the volcanic rock field. These sediments have a constant Sr value, medium–low Rb values, and medium–high Cr values. The variable proportion of Cr and Rb are be due to different quantities of pyroxene, olivine, mica, and K-feldspar.
2. Group 2 (Volcanic-dominated): nearly constant content of Rb and Cr (median values Rb/Cr = 1.25) and more variable Sr. This group refers to a mixing process between volcanic and sedimentary components, though the former is preponderating over the latter (orange circles).
3. Group 3 (Sedimentary-dominated): relatively constant Sr values, high Cr values, and low Rb values (yellow circles). This cluster is related to a mixing process between the volcanic and sedimentary components, where the carbonate component prevails. This is also confirmed by the fact that the PTF rock (sandstone), falling within this cluster, is characterized by the presence of sialic minerals such as K-feldspar, which are typical of volcanic material.
4. Group 4 (Sedimentary): streams of sedimentary origin (green circles) and distributed along the line depicted from the Sr vertex in Fig. 8, with a relatively constant Rb/Cr ratio of about 0.62.

**Fig. 9:**
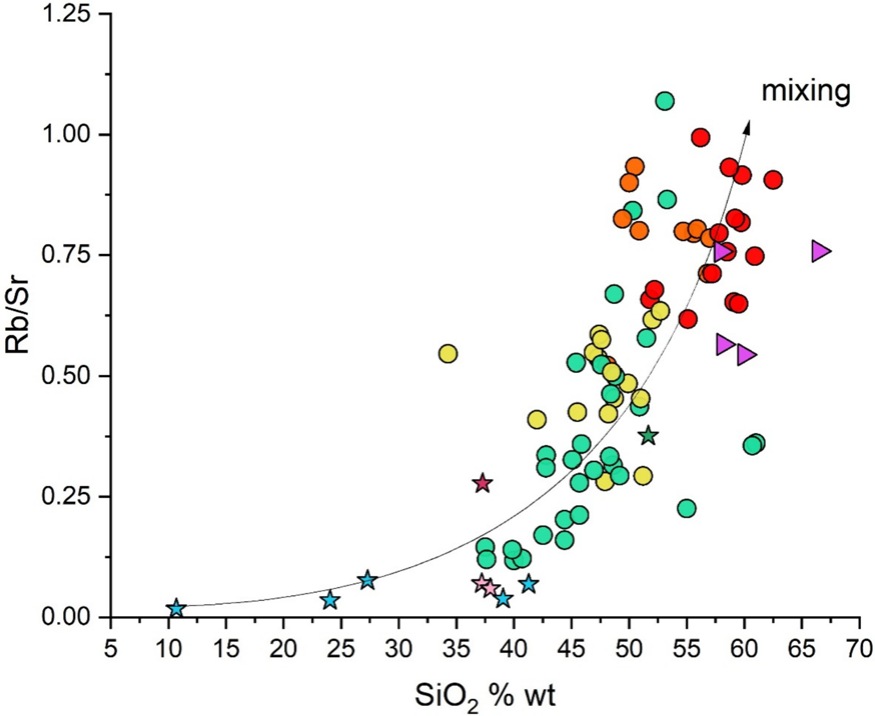
SiO_2_ (wt. %) vs. Rb/Sr binary diagram for stream sediments and the studied rocks. Symbols as in Fig. 8

**Fig. 10:**
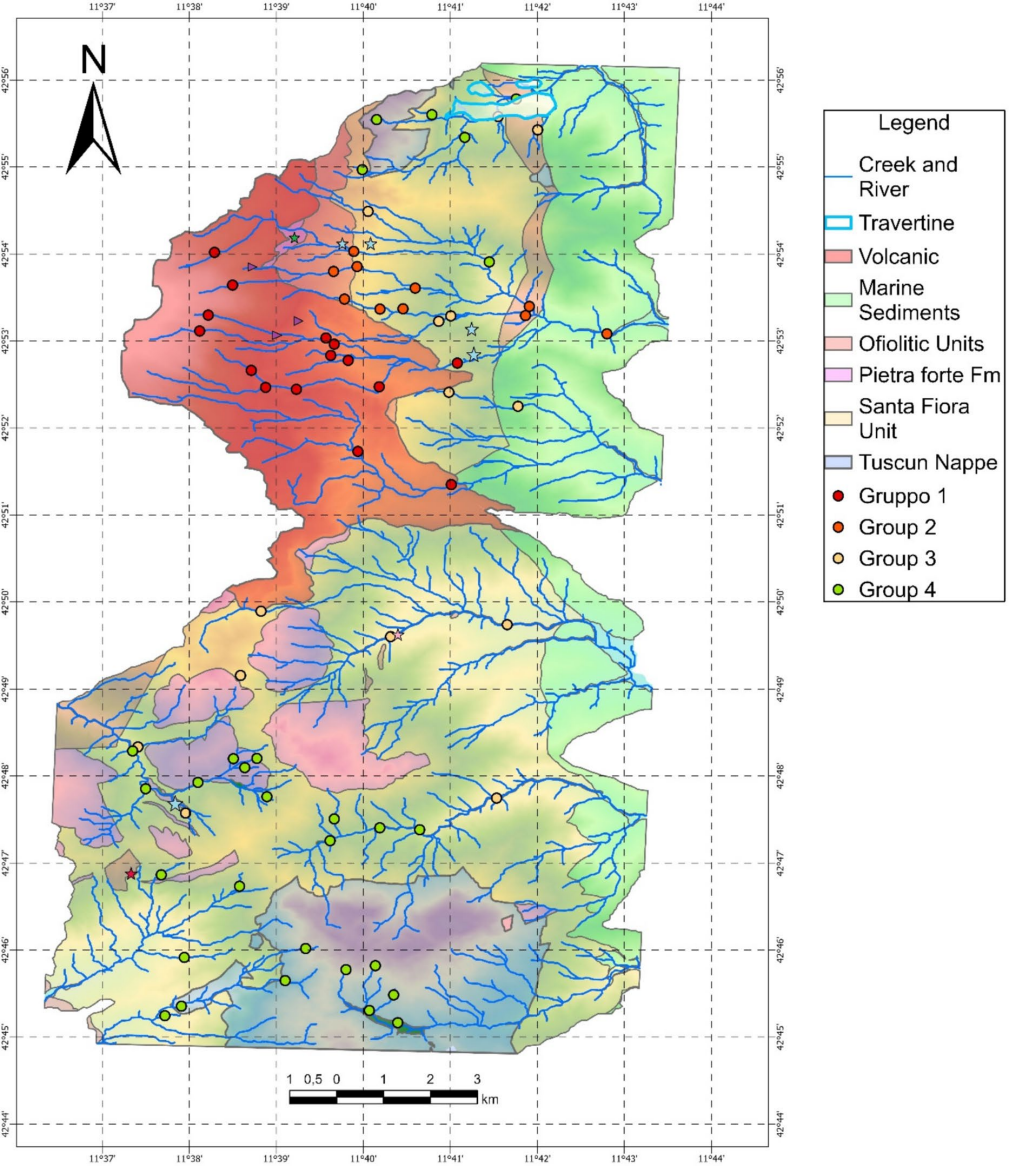
Dot-map of the four different groups with respect to the geology of the study area. Symbols as in Fig. 8

The SiO_2_ vs. Rb/Sr binary plot of Fig. 9 and the dot-maps in Fig. 10, where the distribution of the four groups of Fig. 8 is reported, further supports the physical mixing process between a volcanic and sedimentary component. Figure 9 shows a gradual transition from samples of limestone origin with low silica content and low Rb/Sr ratios to sediments of volcanic origin with high SiO_2_ values (> 50 wt.%) and high Rb/Sr ratios (> 0.5) due to the presence of quartz and K-feldspar and mafic minerals. The result of the mixing process can clearly be seen in Fig. 10, where the stream sediments are divided into the four identified groups. The further we move away from the Mt. Amiata volcanic complex (Fig. 10), the femic component decreases and eventually disappears (Table S2 in supplementary material S1) and the Rb/Sr ratio tends to decrease.

**Fig. 11:**
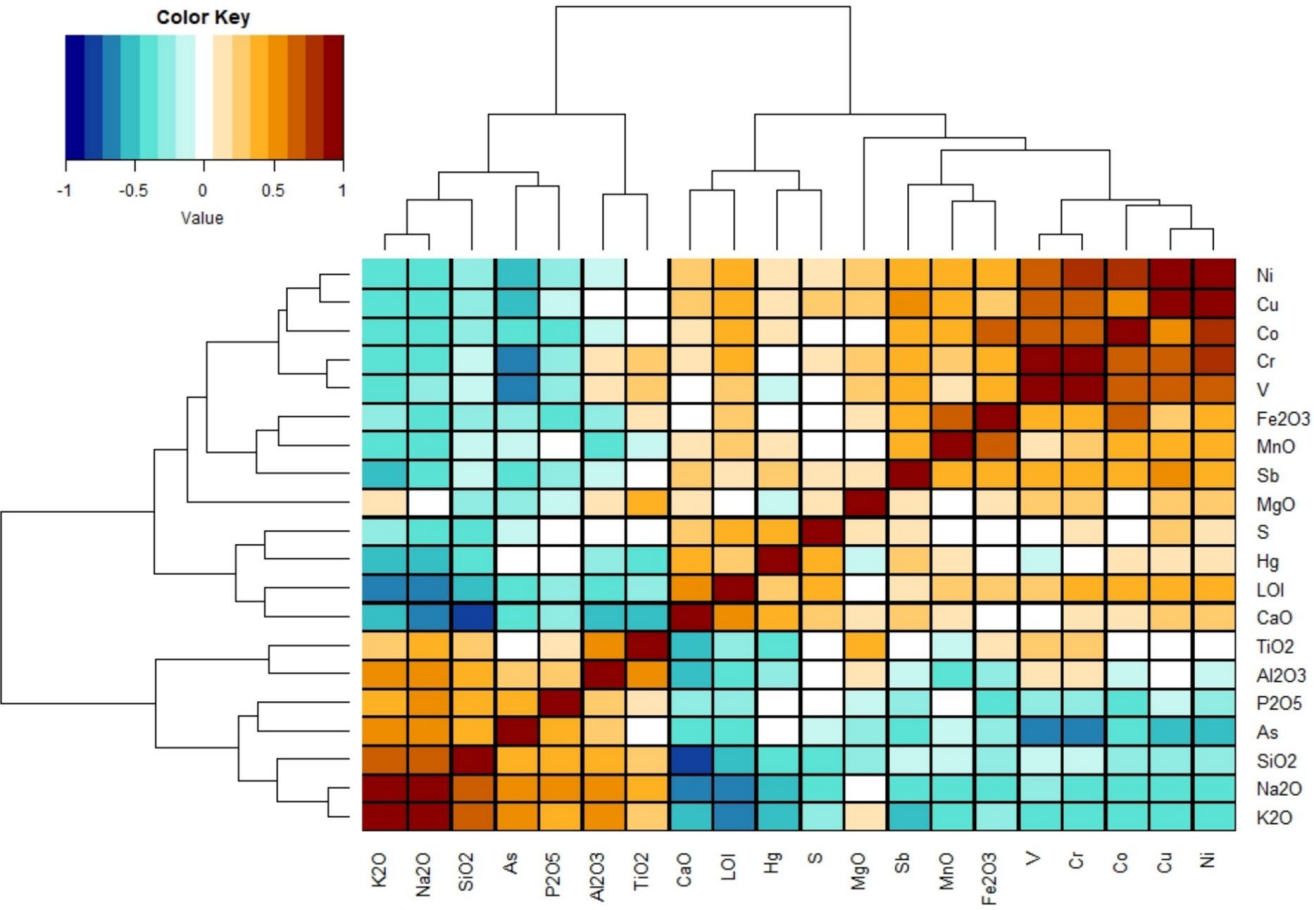
Correlation matrix (Spearman’s correlation type) with hierarchical clustering of the major oxides and selected trace metals. The R value of Spearman correlation is reported in the schematic color legend at the top left corner

The Spearman’s correlation map (Fig. 11), with hierarchical clustering, is illustrated to understand the possible relation between major oxides and trace elements. The clustering in Fig. 11 shows, even in this case, the presence of four groups, as follows:

1. K_2_O, Na_2_O, SiO_2_, P_2_O_5_ and As. This cluster (Volcanic) reflects the abundance of quartz, plagioclase and other silicate minerals and accessory minerals such as apatite, as also evidenced by XRD analysis. The positive correlation between As and K_2_O and Na_2_O (R = 0.5) is more lithological than chemical since As is associated with the volcanic products (Meloni et al., 2023b; Rajendran et al., 2024; Raju, 2022).
2. Al_2_O_3_ and TiO_2_. These two elements (R = 0.6) are highly resistant to chemical alteration. According to Salminen et al., (2005), high values of TiO_2_ and Al_2_O_3_ in stream sediments (> 0.82% and > 13.4%, respectively) are found in areas where crystalline basement rocks of intermediate to mafic signature area occur due to the abundance of detrital oxides, feldspars and phyllosilicate minerals. This reflects the concentrations of the Volcanic-dominated group where the mean value of TiO_2_ is 0.81% and that of Al_2_O_3_ is 19.97%, the highest contents being 0.95% and 22.31% respectively.
3. MgO, Sb, Fe_2_O_3_, MnO, V, Cr, Co, Cu and Ni. The relatively high R-value (> 0.5 to 1) between Fe_2_O_3_, MgO, and MnO and the siderophile PTEs highlight that the Sedimentary-dominated group is partly affected by a geochemical component derived from mafic to ultramafic minerals. According to Pandeli et al. (2017), serpentinitic olistolites and ophicalci are indeed present in the ophiolitic units of the Ligurian complex. However, Fe–Mn (oxi)hydroxides and clay minerals (e.g. chlorite) may also contribute to the positive correlation.
4. CaO, LOI, Hg and S. This cluster (Sedimentary) has a weak to good correlation (0.4 < R < 0.6). The statistically significant correlation among Hg, LOI, and S can be explained by the affinity of Hg with organic matter and sulfides, both decomposing at the LOI temperature (950 °C). On the other hand, Hg mineralization in the Mt. Amiata district is mainly associated with carbonate formations, as suggested by the positive correlation between Hg and CaO.

**Fig. 12:**
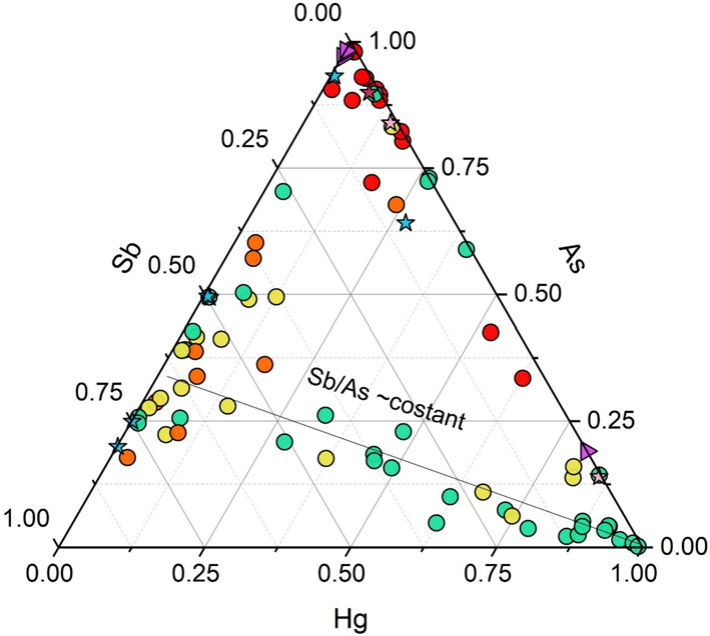
Triangular diagram of Hg, As and Sb for the rocks and stream sediments. Symbols as in Fig. 8

**Fig. 13:**
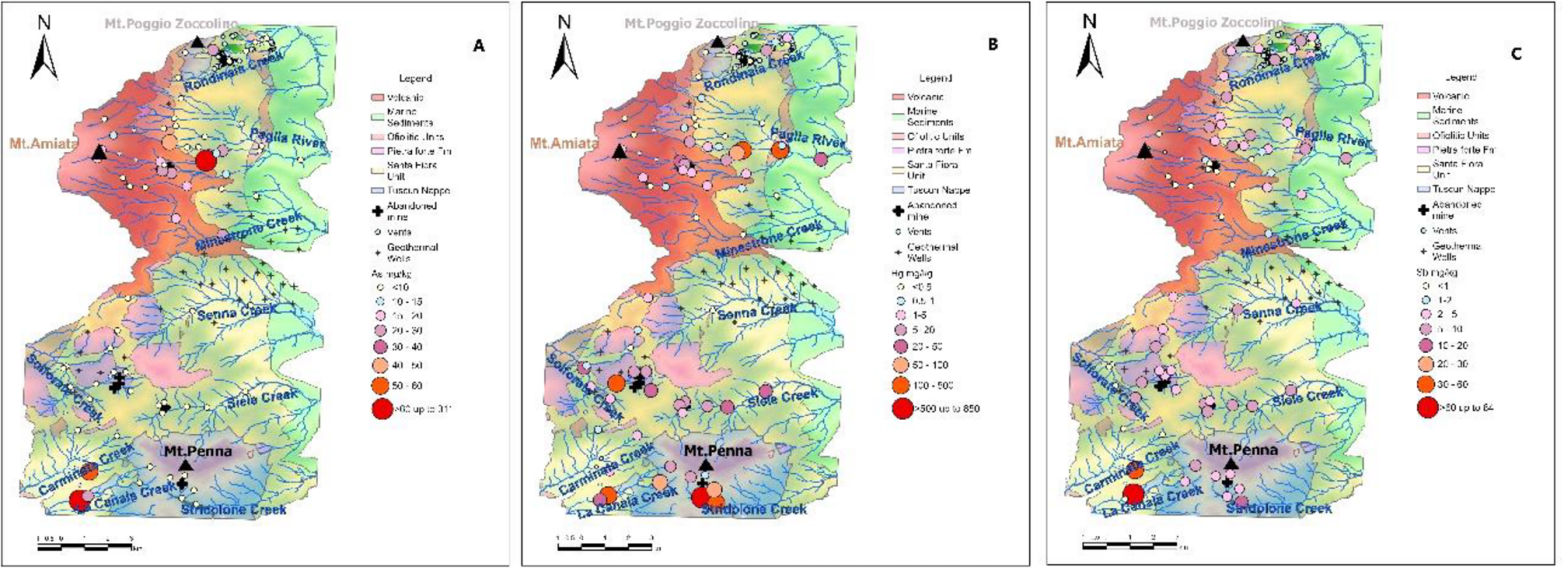
Dot-maps of As (**A**), Hg (**B**) and Sb (**C**) in the stream sediments (in mg/kg). See the text for further details

A more complicated issue is related to the presence of Sb in the Sedimentary-dominated group. In the Mt. Amiata district, stibnite (Sb_2_S_3_) and, subordinately, stibiconite [Sb^3+^Sb^5+^_2_O_6_(OH)] as alteration of stibnite (Brogi et al., 2011) are the most common Sb-minerals although, to the best of our knowledge, specific studies in the study area have not been carried out. The low correlation between Sb and the other elements (0.4 < R < 0.5; Fig. 11) in the Sedimentary-dominated group therefore does not rule out that Sb could be hosted in other minerals. For example, in the Apuan Alps (NW Tuscany) at the Bottino mine, bottinoite (Ni[Sb(OH)_6_]x6H_2_O) was found (Bonazzi et al., 1992). In the nearby Cetine di Cotorniano mine (Siena), secondary Sb minerals are abundant, including schafarzikite (FeSb_2_O_4_) and triphyite (FeSbO_4_). Meloni et al. (2021) suggested the presence of these two minerals, in addition to romeite (Ca_2_Sb_2_O_7_), in the soils from the Lame mining dump (Abbadia San Salvatore mine). The low correlation (R = 0.3) between Hg and Sb seems to agree with Brogi et al. (2011), who stated that Hg is often found associated with silicified carbonates rocks such as jaspers, although it was also recognized in sandstone formations (e.g. Pietraforte Fm.) and mineralized clays called Biocca (Dini, 2017), mostly consisting of kaolinite and montmorillonite. Consequently, a distinct origin for Hg and Sb is highly probable and related to two distinct periods of mineralization (Brogi et al., 2011). The Sb-rich fluids would indeed have been emplaced after the formation of the Hg-rich deposits. It should be noted that Hg and Sb are rarely correlated each other (Arisi Rota et al., 1971 and reference therein). According to many authors (e.g. Stea, 1971; Lattanzi, 1999; Rimondi, 2015), Sb ore deposits show a preferred association with the tectonic contact between the Triassic Tuscan formation and the overlying Ligurian Flysch. Moreover, Lotti (1910) reported that Sb-rich ore deposits were associated with hydrothermal vents (also called “*putizze*”, Tassi et al., 2009). It is to mention that some ore deposits such as that of Abbadia San Salvatore are indeed Sb-poor whereas those of Morone are characterized by a higher concentration of Sb. Additionally, the decoupling between Hg and Sb and the association between the tectonic contact and the “*putizze*” area are further supported by the Hg, As and Sb triangular diagram in Fig. 12. In fact, on the Sb side, some samples, mainly from the Pietrineri mine area, the Morone mine, and some sites located along the Pagliola stream, are positioned (Fig. 13C). Here, in addition to the Sb mineralization (Brogi et al., 2011; De Castro, 1914; Rimondi et al., 2015), the “*putizze*” area and the tectonic contact between the Triassic Tuscan Fm. and the Ligurian Flysch are also present. As shown in Fig. 13B, high Hg concentrations (> 1 mg/kg, i.e. the Italian Legal Limit for soils destined to residential and public areas) are located near the abandoned mines or along the main rivers draining them. On the other hand, Sb is homogeneously distributed over the entire study area, except in the volcanic complex (Fig. 13C), where the lowest concentrations are recorded. Concentrations between 60 < Sb < 200 mg/kg are located in the southern part of the study area, near the Morone mine, confirming the hypothesis that Hg and Sb mineralization occurred asynchronously.

As far as arsenic is concerned, volcanic rocks (purple triangles), Volcanic group and fluvial sediments from the Solforate stream, showing high concentrations of As (Fig. 13A) similar to those recorded in the hydrothermal areas (e.g. Bagni San Filippo) or close to the gas vents (Fig. 13B and C), are positioned on the upper right of the Hg-As-Sb diagram (Fig. 12), also showing high Hg (Fig. 13B).

Fluvial sediments deposited by sedimentary formations are distributed along the straight line dictated by a relatively constant Sb/As ratio (mean value Sb/As = 1.09) and a variable amount of Hg (Fig. 12), showing how As and Sb are evenly distributed in the Sedimentary and Sedimentary-dominated groups (Fig. 13A and C).

### Geochemical background values for the stream sediments

One of the aims of this work was to compute the geochemical background values of potential toxic elements (PTEs, i.e. Hg, As, Sb, Cr, V, Ni, Co, Cu). The Italian environmental authorities (SNPA, 2017) require these values to be obtained with samples analyzed after aqua regia digestion, and with EPA 7473 method for Hg (Table S2, Supplementary Material S2). Although river sediments are not regulated under the Italian and European Environmental Legislation, defining natural geochemical background values or threshold values can help authorities to understand the source of potential environmental contaminations. Since river sediments reflect rock and soil weathering, in order to compute geochemical background values and according to Figs. 8 and 9, sediments were divided into two clusters: (1) Volcanic and Volcanic-dominated, and (2) Sedimentary and Sedimentary-dominated.

The background values are calculated with the software ProUCL 5.2. If the dataset follows a normal distribution, the background values are computed with the UTL 95-95 for volcanic and sedimentary provenance of the stream sediments. The gamma distribution UTL95-95 is preferred when the distribution is both log-normal and gamma. After checking outlier tests and identifying each one in the Q-Q and box plots, any possible outlier is removed. The geochemical background values, as defined by Reiman and de Caritat (2017), Santos-Frances et al. (2017) and Meloni et al. (2023b), should be referred to a range of concentrations for a particular location, rather than to a single number, being each concentration impacted by an analytical uncertainty. Thus, for the defined distributions, a confidence interval of ± 10% is applied to account for measurement uncertainty. Tables 6 and 7 report the type of distribution, the 95th and 99th percentiles of the distribution with no outliers, the median, and the recommended geochemical background for clusters 1 and 2, respectively. Furthermore, if after the elimination of outliers, the dataset shows two or more populations, these are split up and studied separately. This is the case of Co, Ni and Cu in cluster 1, and As and Sb in cluster 2. Concerning cluster 1, two populations can be identified for Co, Ni, and Cu. The first one is characterized by those samples of purely volcanic origin that had concentrations below 6, 15 and 11 mg/kg, respectively, whilst the second population corresponds to the previously identified Group 2 (Figs. 8 and 9). For each of these two identified populations, the distribution type is then reviewed, and a reference value is assessed for each one, as shown in Table 6.

**Table 6:** PTEs, type of distribution (Dist), 95% percentile, 99% percentile, median, and proposed background values for cluster 1; – none

PTEs	Dist	95%ile	99%ile	Median	95% UTL95%	Background value
Hg	G/LN	3.4	4.6	0.6	5.72	5.2–6.3
Sb	N	7.5	9.0	4.1	9.5	8.5–10.5
As	G/LN	30.8	44.3	9.4	44.1	39.7–48.5
Crtot	G/LN	36.1	48.7	15.0	47.8	43.0–52.6
V	N	30.3	34.7	19.7	34.6	31.2–38.1
Co population 1	N	5.5	6.2	3.6	6.5	5.9–7.2
Co population 2	N	17.6	19.6	11.8	21.1	19–23.2
Ni population 1	N	9.0	10.1	6.6	10.6	9.6–11.6
Ni population 2	N	45.7	52.9	29.1	57.2	51.5–62.9
Cu population 1	LN	6.9	8.6	3.9	–	3.71–4.54
Cu population 2	N	61.3	73.1	32.6	80.2	72.2–88.2

**Table 7:** PTEs, type of distribution (Dist), 95% percentile, 99% percentile, median, and proposed background values for cluster 2, – none

PTEs	Dist	95%ile	99%ile	Median	95%UTL95%	Background value
Hg	G/LN	8.5	13.9	5.0	13.64	12.3–15.0
Sb	LN	7.1	8.6	4.3	–	4.92–6.01
As	N	6.07	7.11	3.0	6.79	6.11–7.47
Co	N	26.3	31.0	13.1	29.27	26.3–32.2
Ni	G/LN	49.1	63.5	21.8	58.7	52.8–64.6
Cu	N	61.9	73.5	29.2	69.04	62.1–75.9
Crtot	N	51.5	60.1	29.2	56.8	51.1–62.5
V	G/LN	47.3	59.9	16.5	55.7	50.13–61.3

In cluster 2, As and Sb are found in populations 2 and 3, respectively. The first As population is characterized by contents up to 11 mg/kg, whilst the second population has > 21 mg/kg. This population includes those samples that owe the As enrichment to the presence of hydrothermal vents (e.g. Bagni San Filippo, Morone), mineralization or inputs slightly influenced by volcanic lithology. Excluding this outlier population, the first population thus represents the background.

Antimony is characterized by three populations: the first population, with values > 11 mg/kg, consists of 3 samples (STA46, STA51, and STA53, in watersheds 7, 8, and 9, respectively), belonging to areas with Sb mineralization. The second population is instead characterized by samples with concentrations between 1 and 11 mg/kg, representing the background population. The third population includes those samples with values below < 1 mg/kg, corresponding to the instrumental detection limit.

Comparing the computed background values (last column in Tables 6 and 7) with those reported in the FOREGS database for the same elements, As results to have a background value for cluster 1 higher than that proposed by FOREGS (10–15 mg/kg). The high As values are due to the presence of volcanic lithologies (Fig. 10). Differently, Co is higher in cluster 2 with respect to cluster 1. For the stream sediments, the FORGES interval values for Co, Cr, Ni, V, and Cu are 13–17, 41–72, 38–71, 49–68, 30–43 mg/kg, respectively. These are lower for Co, V, and Cu in Table 7 and Co, Ni, and Cu in Table 6 for population 2, while they are higher than those for Co, Ni, and Cu in Table 6 for population 1. This is because Co, as well as Ni and V, are enriched in the stream sediments of sedimentary provenance, while they are depleted in those of volcanic origin. In contrast, Cr in Table 6, and Cr and Ni values in Table 7 are in agreement with the range reported in the FOREGS database. Mercury, in both clusters, is higher than that listed in the FOREGS dataset (0.093–0.2 mg/kg). On the other hand, the Sb values after extraction by aqua regia are not available in the FOREGS database. Protano et al. (1998) reported background geochemical values of Hg, Sb and As in stream sediments from southern Tuscany equal to 0.15, 0.4, and 7 mg/kg, respectively. These values, as well as the Hg background value (2–9 mg/kg) in the Siele area proposed by Fornasaro et al. (2022), result to be remarkably lower than those proposed in this study.

### Hg speciation by thermal desorption

**Fig. 14:**
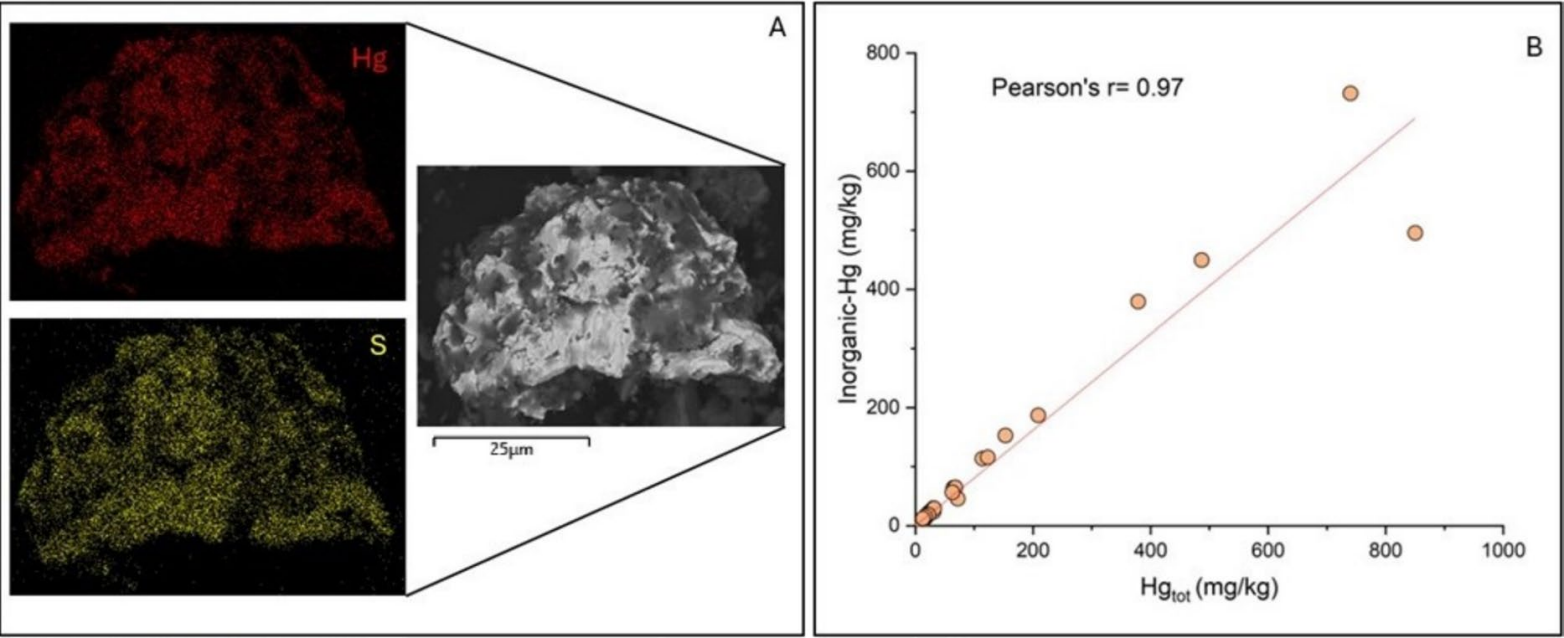
**A** clast of cinnabar by SEM images in the sample STA 22; **B** scatterplot of Hg_tot_ vs. inorganic (α-HgS + β-HgS) Hg in the stream sediments

When high concentrations of Hg are found, like in this study, assessing whether it is potentially bioavailable, i.e. able to participate in the biogeochemical cycling between water, sediment and biological communities, is critical. As reported in Table 5, Hg is mainly presented as α-HgS and β-HgS. The presence of α-HgS or β-HgS scattered in the matrix or in crystals of primary origin is further confirmed by Scanning Electron Microscope (SEM, Fig. 14A) investigations carried out on selected sediment samples (e.g. GIT4, STA 4 in watershed 2, Salto 1, STA 22, STA 42 in watershed 5, and STA 53 in watershed 8).

As reported by Meloni et al. (2023a), soils from the Abbadia San Salvatore mine showed a significant correlation (r-Pearson’s = 0.95) between inorganic Hg (α-HgS + β-HgS) and total Hg (Fig. 14B), whereas no relationship was found between OM-bound Hg and total Hg, as well as between Hg from leachates and Hg-OM, nor between % OM and Hg-OM. Only sample STA 47 (watershed 7) and Salto 4 (watershed 5) present HgSO_4_ and HgCl_2_, respectively. This reflects the fact that these two samples are the only streams to show concentrations of leachable Hg higher than that imposed by the Italian Environmental Legislation (1 µg/L; Table 4). In fact, both HgSO_4_ and HgCl_2_ are relatively soluble in water (logKps: −9.41 and −21.26 mol/L, respectively; Allison et al., 1990) when compared to α-HgS and β-HgS (Ariya et al., 2015). As reported in Table 5, MSIE11 (watershed 6), 9171A22 (watershed 8) and 9081A22 (watershed 5) are the only streams with high % of β-HgS (> 70%). These three samples were taken within three different former mining areas: the first one is from the Siele mine and the second in the Morene mine and the last from the Abetina-Solforate mine. According to Kim et al. (2004), the roasting of ores produced elevated β-HgS levels in calcined ore waste. Therefore, it is likely that β-HgS refers to the presence of roasting ore inside the stream sediments. This aspect was also underlined by Petranich et al. (2022), who stated that marine sediments from the Gulf of Trieste (NE Italy) are enriched in β-HgS, due to waters and suspended solids of the Isonzo river, which drains the former Hg mine of Idrija (Slovenia). Although the reclamation at Siele was completed in 2001, according to Fornasaro et al. (2022), the high Hg concentrations in the stream sediments and the presence of β-HgS are ascribable to a mismanagement of the local mining tailings, the latter being removed by meteoric waters and transported into the Siele. A similar situation is occurring in the Solforate/Abetina and Morone mines, although no reclamation activities are currently going on. At this mine, intense rainfalls have caused slumping and small landslides that have affected the mining waste and partly dumped into Solforate stream, contributing to an increased input of calcine into the stream itself.

However, the presence of other Hg soluble compounds within the analyzed sediments cannot be excluded. Petranich et al. (2022) indeed suggested that the TD technique may fail when Hg-bearing salts occur at low amount to be related to specific disassociation temperatures of pure chemical compounds. Nevertheless, the large predominance of insoluble inorganic species (i.e. cinnabar and metacinnabar) in the stream sediments from the eastern sector of Mt. Amiata points to a very limited availability to the biota and thus, to the Hg biogeochemical cycle.

## Conclusions

Stream sediment geochemistry and determination of the background values in the eastern portion of the Mt. Amiata (Southern Tuscany) were defined with the use of XRF and ICP-AES analysis and the Pro-UCL 5.2 software, respectively. Based on the Rb–Sr-Cr ternary diagram, the sources of stream sediments can be divided into four groups: (1) Volcanic, (2) Volcanic-dominated, (3) Sedimentary-dominated and (4) Sedimentary. The presence of physicochemical intermediate features between volcanic and sedimentary lithologies is confirmed by the SiO_2_ vs. Rb/Sr plot and supported by the As, Hg and Sb dot-maps where the distribution of the four groups is clearly evidenced (Fig. 10).

Chromium, Co, V, Cu and Ni derive from a mafic and ultramafic component, occurring within the Ligurian Unit, that characterizes the study area (e.g. scattered ophiolite outcrops) and are enriched in the finest material. Mercury, as reported in the dot-map of Fig. 13B, is associated with the mineralized and the former mining areas, as well as with the organic matter. Arsenic is found to be concentrated in the Mt. Amiata volcanic apparatus and in correspondence of hydrothermalized zones and gas vents. Antimony, on the other hand, is homogeneously distributed except in the Volcanic group, whose concentrations result to be lower when compared to the other groups. The highest concentrations are indeed referred to the southern part of the study area where Hg-Sb mineralization occurs.

In agreement with the four individuated groups, for the calculation of background values, groups 1 and 2 and groups 3 and 4 were combined in two different clusters. The determination of geochemical background values in river sediments, although not regulated by Italian and European legislation, is of great importance. In fact, they can allow the definition of reasonable post-extractive reclamation objectives in any decommissioned mine, as it is the case of those distributed in the Mt. Amiata area. Stream sediments may indeed reflect the environmental impact on surface waters that are crosscutting or flowing close to where former mining activities were operating.

Through thermal desorption, Hg speciation in stream sediments was successfully performed. By comparing leached Hg and Hg speciation data, Hg can unlikely be dispersed into the environment. Inorganic, insoluble Hg-species (i.e., cinnabar and metacinnabar) are indeed largely dominating the stream sediments where the total Hg content was > 5 mg/kg. This suggests a low bioavailability of Hg. The same results were also evidenced in soils from the former mining area of Abbadia San Salvatore.

## Supplementary Information

Below is the link to the electronic supplementary material.

Supplementary file1 (DOCX 38 KB)

Supplementary file2 (DOCX 4288 KB)

Supplementary file3 (DOCX 71 KB)

## Data Availability

No datasets were generated or analysed during the current study.

## References

[CR1] Allison, J.D., Brown, D.S., Novo-Gradac, K.J. (1990). MINTEQA2/PRODEFA2—A Geochemical Assessment Model for Environmental Systems—Version 3.0 User’s Manual; Environmental Research Laboratory, Office of Research and Development U.S. Environmental Protection Agency: Athens, GA, USA, 1990.

[CR2] Amireh, B. S., Saffarini, G. A., Amaireh, M. N., Jarrar, C. H., & Abed, A. A. (2022). Rare-earth and trace elements of the lower Cambrian-Lower Cretaceous siliciclastic succession of NE Gondwana in Jordan: From provenance to metasomatism. *Annales Societatis Geologorum Poloniae*. 10.14241/asgp.2022.05

[CR3] Arisi Rota, F., Brondi, A., Dessau, G., Franzini, M., Stea, B., & Vighi, L. (1971). I Giacimenti minerari. In: La Toscana Meridionale-Fondamenti geologico-minerari per una prospettiva di valorizazione delle risorse naturali, Fascicolo speciale dei Rendiconti della Società Italiana di Mineralogia e Petrologia, 27,442-501. In Italian.

[CR4] Ariya, P. A., Amyot, M., Dastoor, A., Deeds, D., Feinberg, A., Kos, G., Poulain, A., Ryjkov, A., Semeniuk, K., Subir, M., & Toyota, K. (2015). Mercury physicochemical and biogeochemical transformation in the atmosphere and at atmospheric interfaces: A review and future directions. *Chemical Reviews,**115*, 3760–3802. 10.1021/cr500667e 25928690 10.1021/cr500667e

[CR5] Bhatia, M. R., & Crook, A. W. (1986). Trace element characteristics of graywackes and tectonic setting discrimination of sedimentary basins. *Contributions to Mineralogy and Petrology,**92*, 181–193.

[CR6] Bianchini, G., Natali, C., Di Giuseppe, D., & Beccaluva, L. (2012). Heavy metals in soils and sedimentary deposits of the Padanian Plain (Ferrara, Northern Italy): Characterisation and biomonitoring. *Journal of Soils and Sediments,**12*, 1145–1153.

[CR7] Biester, H., & Scholz, C. (1996). Determination of mercury binding forms in contaminated soils: Mercury pyrolysis versus sequential extractions. *Environmental Science & Technology,**31*(1), 233–239. 10.1021/es960369h

[CR8] Bonazzi, P., Menchetti, S., Caneschi, A., & Magnanelli, S. (1992). Bottinoite, Ni(H_2_O)_6_[Sb(OH)_6_]_2_, a new mineral from the Bottino mine, Alpi Apuane, Italy. *American Mineralogist,**77*(11–12), 1301–1304.

[CR9] Bonham-Carter, G. F., & Goodfellow, W. D. (1986). Background corrections to stream geochemical data using digitized drainage and geological maps: application to Selwyn Basin, Yukon and Northwest Territories. *Journal of Geochemical Exploration,**25*, 139–155. 10.1016/0375-6742(86)90011-7

[CR10] Bourdeau, J. E., Zhang, S. E., Nwaila, G. T., & Ghorbani, Y. (2024). Data generation for exploration geochemistry: Past, present and future. *Applied Geochemistry*. 10.1016/j.apgeochem.2024.106124

[CR11] Brogi, A., & Fabbrini, L. (2010). The Monte Penna thrust (southern Tuscany, Italy): Geometric and kinematic of a collisional structure affecting the Tuscan Nappe during the Northern Apennines orogenic building. *Italian Journal of Geosciences,**129*, 74–90. 10.3301/IJC.2009.07

[CR12] Brogi, A., Fabbrini, L., & Liotta, D. (2011). Sb-Hg ore deposit distribution controlled by brittle structures: The case of the Selvena mining district (Monte Amiata, Tuscany, Italy). *Ore Geology Reviews,**41*, 35–48.

[CR13] Buccianti, A., Egozcue, J. J., & Pawlowsky-Glahn, V. (2008). Another look at the chemical relationships in the dissolved phase of complex river systems. *Mathematical Geosciences,**40*, 475–488. 10.1007/s11004-008-9168-2

[CR14] Carranza, E. J. M. (2008). *Geochemical Anomaly and Mineral Prospectivity Mapping in GIS* (1st ed.). Elsevier.

[CR15] De Castro, G. (1914). Le miniere di mercurio del M. Amiata. *Mem. Descr. della Carta Geol. d’It. v. XVI*, a cura del R. Uff. Geol. TIP. L. Cecchini, Roma, pp. 1–219., Amiata. (In Italian).

[CR16] Cave, M.R., Johnson, C.C., Ander, E.L., & Palumbo-Roe, B. (2012). Methodology for the determination of normal background contaminant concentrations in English soils. In: *British Geological Survey Commissioned Report*, CR/12/003. Available at: http://nora.nerc.ac.uk/19959/.

[CR17] Cheng, Q. (2007). Mapping singularities with stream sediment geochemical data for prediction of undiscovered mineral deposits in Gejiu, Yunnan Province, China. *Ore Geology Reviews,**32*, 314–324. 10.1016/j.oregeorev.2006.10.002

[CR18] Chiodini, G., Cardellini, C., Caliro, S., Avino, R., Donnini, M., Granieri, D., Morgantini, N., Sorrenti, D., & Frondini, F. (2020). The hydrotermal system of Bagni San Filippo (Italy): fluid circulation and CO2 degassing. *Italian Journal of Geosciences,**139*, 383–397.

[CR19] Colica, A., Benvenuti, M., Chiarantini, L., Costagliola, P., Lattanzi, P., Rimondi, V., & Rinaldi, M. (2019). From point source to diffuse source of contaminants: The example of mercury dispersion in the Paglia River (Central Italy). *CATENA,**172*, 488–500. 10.1016/j.catena.2018.08.043

[CR20] Condie, K. C. (1993). Chemical composition and evolution of the upper continental crust: Contrasting results from surface samples and shales. *Chemical Geology,**104*, 1–37.

[CR21] Conticelli, S., Boari, E., Burlamacchi, L., Cifelli, F., Moscardi, F., Laurenzi, M. A., Ferrari, P. L., Francalanci, L., Benvenuti, M. G., Braschi, E., & Moretti, P. (2015). Geochemistry and Sr-Nd-Pb isotopes of Monte Amiata volcano, Central Italy: Evidence for magma mixing between high-K calc-alkaline and leucititic mantle-derived magmas. *Italian Journal of Geosciences,**132*(2), 266–290. 10.3301/IJG.2015.12

[CR22] Cox, R., Lowe, D. R., & Cullers, R. L. (1995). The influence of sediment recycling and basement composition on evolution of mudrock chemistry in the southwestern United States. *GCA,**59*(14), 2919–2940.

[CR23] D’Orazio, M., Campanella, B., Bramanti, E., Ghezzi, L., Onor, M., Vianello, G., Vittori-Antisari, L., & Petrini, R. (2020). Thallium pollution in water, soils and plants from a past-mining site of Tuscany: Sources, transfer processes and toxicity. *Journal of Geochemical Exploration,**209*, 106434. 10.1016/j.gexplo.2019.106434

[CR24] Dall’Aglio, M., Da Roit, R., Orlandi, C., & Tonani, F. (1966). Prospezione geochimica del mercurio. Distribuzione del mercurio nelle alluvioni della Toscana. *L’industria Mineraria,**17*, 391–398. (In Italian).

[CR25] Dinelli, E., Cortecci, G., Lucchini, F., & Zantedeschi, E. (2005). Sources of major and trace elements in the stream sediments of the Arno river catchment (northern Tuscany, Italy). *Geochemical Journal,**39*, 531–545.

[CR26] Dini, A. (2003). Ore deposits, industrial minerals, and geothermal resources. *Periodico di Mineralogia,**72*, 41–52.

[CR27] Dini, A. (2017). Mines and minerals in the Mining district of Monte Amiata. In: E.S.A. Edizioni Scientifiche e Artistiche (Ed.), *Il Vulcano di Monte Amiata*. Nola (NA), Italy, pp. 343–369.

[CR28] Dini, A., Rielli, A., Di Giuseppe, P., Ruggieri, G., & Boschi, C. (2024). The Ophiolite-Hosted Cu-Zn VMS Deposits of Tuscany (Italy). *Minerals,**14*, 273. 10.3390/min14030273

[CR29] Doherty, M. E., Arndt, K., Chang, Z., Kelley, K., & Lavin, O. (2023). Stream sediment geochemistry in mineral exploration: a review of fine-fraction, clay-fraction, bulk leach gold, heavy mineral concentrate and indicator mineral chemistry. *Geochemistry: Exploration, Environment, Analysis*. 10.1144/geochem2022-039

[CR30] Farahbakhsh, E., Chandra, R., Eslamkish, T., & Müller, R. D. (2019). Modeling geochemical anomalies of stream sediment data through a weighted drainage catchment basin method for detecting porphyry Cu-Au mineralization. *Journal of Geochemical Exploration,**204*, 12–32.

[CR31] Faraone, D., & Stoppa, F. (1990). Petrology and regional implication of Early Cretaceous alkaline lamprophyres in the Ligure-Maremmano group (Southern Tuscany, Italy): An outline. *Ofioliti,**15*, 45–59.

[CR32] Feng, R., & Kerrich, R. (1990). Geochemistry of fine-grained clastic sediments in the Archaean Abitibi Greenstone Belt, Canada: Implications for provenance and tectonic setting. *GCA,**54*, 1061–1081.

[CR33] Forconi, S. (2011). Il Cinabro Sul Monte Amiata. *Stampa,**2000*, 223. In Italian.

[CR34] Fornasaro, S., Morelli, G., Rimondi, V., Fagotti, C., Friani, R., Lattanzi, P., & Costagliola, P. (2022). Mercury distribution around the Siele Hg mine (Mt. Amiata district, Italy) twenty years after reclamation: Spatial and temporal variability in soil, stream sediments, and air. *Journal of Geochemical Exploration,**232*, 106886. 10.1016/j.gexplo.2021.106886

[CR35] Garrett, R. G., Reimann, C., Smith, D. B., & Xie, X. (2008). From geochemical prospecting to international geochemical mapping: A historical overview. *Geochemistry: Exploration, Environment, Analysis,**8*, 205–217.

[CR36] Gonzalez-Raymat, H., Liu, G., Liriano, C., Li, Y., Yin, Y., Shi, J., Jiang, G., & Cai, Y. (2017). Elemental mercury: Its unique properties affect its behavior and fate in the environment. *Environmental Pollution,**229*, 69–86. 10.1016/j.envpol.2017.04.101 28577384 10.1016/j.envpol.2017.04.101

[CR37] Gozzi, C., Dakos, V., Buccianti, A., & Vaselli, O. (2021). Are geochemical regime shifts identifiable in river waters? Exploring the compositional dynamics of the Tiber River (Italy). *Science of the Total Environment,**785*, 147268. 10.1016/j.scitotenv.2021.147268 33940415 10.1016/j.scitotenv.2021.147268

[CR38] Gozzi, C., Sauro Graziano, R., & Buccianti, A. (2020). Part-whole relations: New insights about the dynamics of complex geochemical riverine systems. *Minerals,**10*(6), 501. 10.3390/min1006050

[CR39] Gustavsson, N., Loukola-Ruskeeniemi, K., & Tenhola, M. (2012). Evaluation of geochemical background levels around sulfide mines–A new statistical procedure with beanplots. *Journal of Applied Geochemistry,**27*(1), 240–249.

[CR40] Holland, H. D. (1978). *The chemistry of the atmosphere and oceans* (p. 369). Wiley-Interscience.

[CR41] Hossain, H. Z., Kawahata, H., Roser, B. P., Sampei, Y., Manaka, T., & Otani, S. (2017). Geochemical characteristics of modern river sediments in Myanmar and Thailand: Implications for provenance and weathering. *Geochemistry,**77*, 443–458.

[CR42] Johnson, C.C., Ander, E.L., Cave, M.R., & Palumbo-Roe, B. (2012). Normal Background Concentrations (NBCs) of Contaminants in English Soils: *Final Project Report. British Geological Survey Commissioned Report*. CR/12/035, 40 pp. Available at: http://nora.nerc.ac.uk/19946/.

[CR43] Kelepertzis, E., Argyraki, A., Daftsis, E. I., Ballas, D. & University of Athens (Greece), Faculty of Geology and Geoenvironment. (2010). Geochemical background heavy metal concentrations of stream sediments at mineralized areas of NE Chalkidiki. *Hellenic journal of geosciences*. 45, 153-162.

[CR44] Kim, C. S., Rytuba, J. J., & Brown, G. E. (2004). Geological and anthropogenic factors influencing mercury speciation in mine wastes: An EXAFS spectroscopy study. *Applied Geochemistry,**19*, 379–393.

[CR45] Kim, K. H., Kabir, E., & Jahan, S. A. (2016). A review on the distribution of Hg in the environment and its human impacts. *Journal of Hazardous Materials,**306*, 376–385. 10.1016/j.jhazmat.2015.11.031 26826963 10.1016/j.jhazmat.2015.11.031

[CR46] Lancianese, V., & Dinelli, E. (2015). Geochemical mapping based on geological units: A case study from the Marnoso-arenacea formation (Northern Apennines, Italy). *Geochemistry,**76*(2), 197–210.

[CR47] Lattanzi, P. (1999). Epithermal precious metal deposits of Italy-an overview. *Mineralium Deposita,**34*, 630–638.

[CR48] Laurenzi, M. A., Braschi, E., Casalini, M., & Conticelli, S. (2015). New ^40^Ar-^39^Ar dating and revision of the geochronology of the Monte Amiata Volcano, Central Italy. *Italian Journal of Geosciences,**134*, 255–265. 10.3301/IJG.2015.11

[CR49] Lazzaroni, M., Vetuschi Zuccolini, M., Nisi, B., Cabassi, J., Caliro, S., Rappuoli, D., & Vaselli, O. (2022). Mercury and Arsenic Discharge from Circumneutral Waters Associated with the Former Mining Area of Abbadia San Salvatore (Tuscany, Central Italy). *International Journal of Environmental Research and Public Health,**19*, 5131. 10.3390/ijerph19095131 35564526 10.3390/ijerph19095131 PMC9103097

[CR50] Le Maitre, R. W. (2002). *Igneous Rocks: a Classification and Glossary of Terms: Recommendations of the International Union of Geological Sciences Subcommission on the Systematics of Igneous Rocks* (p. 236). Cambridge University Press.

[CR51] Lee, C. T. (2018). Geochemical Classification of Elements. In W. M. White (Ed.), *Encyclopedia of Geochemistry. Encyclopedia of Earth Sciences Series* (pp. 545–549). Cham: Springer. 10.1007/978-3-319-39312-4_255

[CR52] Levinson, A. (1974). Introduction to Exploration Geochemistry*.* Applied Publishers.

[CR53] Losacco, U. (1960). Ricerche geologiche nella Toscana meridionale. I) Stratigrafia e tettonica del gruppo del M. Civitella-M. Elmo (Grosseto). *Bollettino Societa Geologica Italiana,**78*, 12–43. (In Italian).

[CR54] Lotti, B. (1910). La geologia della Toscana, in Memorie descrittive della Carta Geologica d’Italia, Vol. 13, *Regio Ufficio Geologico. Tipografia Nazionale*, Roma. (In Italian).

[CR55] Marroni, M., Moratti, G., Costantini, A., Conticelli, S., Benvenuti, M. G., Pandolfi, L., Bonini, M., Cornamusini, G., & Laurenzi, M. A. (2015). Geology of the Monte Amiata region, Southern Tuscany, Central Italy. *Italian Journal of Geosciences,**134*, 171–199. 10.3301/IJG.2015.13

[CR56] Meloni, F., Montegrossi, G., Lazzaroni, M., Rappuoli, D., Nisi, B., & Vaselli, O. (2021). Total and leached arsenic, mercury and antimony in the mining waste dumping area of Abbadia San Salvatore (Mt. Amiata, Central Italy). *Applied Sciences,**11*, 7893. 10.3390/app11177893

[CR57] Meloni, F. (2022). Determinazione dei valori di fondo di Cr Co, Ni nel Bacino del Torrente Stura (Comune di Barberino di Mugello). *Il Geologo,**16*, 27–33. (In Italian).

[CR58] Meloni, F., Farieri, A., Higueras, P. L., Esbrí, J. M., Nisi, B., Cabassi, J., Rappuoli, D., & Vaselli, O. (2023a). Mercury distribution in plants and soilsfrom the former mining area of Abbadia San Salvatore (Tuscany, Central Italy). *Environmental Geochemistry and Health,**45*(11), 8523–8538. 10.1007/s10653-023-01739-w 37648955 10.1007/s10653-023-01739-w PMC10611595

[CR59] Meloni, F., Nisi, B., Gozzi, C., Rimondi, V., Cabassi, J., Montegrossi, G., Rappuoli, D., & Vaselli, O. (2023b). Background and geochemical baseline values of chalcophile and siderophile elements in soils around the former mining area of Abbadia San Salvatore (Mt. Amiata, southern Tuscany, Italy). *Journal of Geochemical Exploration,**255*, 107324.

[CR60] Meloni, F., Montegrossi, G., Cabassi, J., Bianchi, F., Nisi, B., Rappuoli, D., & Vaselli, O. (2024a). Geochemical Surveys of Ground and Surface Waters in the Abandoned Hg-Mine of Abbadia San Salvatore (Central Italy): A preparatory investigation before remediation. *Water,**16*(9), 1210.

[CR61] Meloni, F., Higueras, L. P., Cabassi, J., Nisi, B., Rappuoli, D., & Vaselli, O. (2024b). Thermal desorption technique to speciate mercury in carbonate, silicate, and organic-rich soils. *Chemosphere,**365*, 143349. 10.1016/j.chemosphere.2024.143349 39278331 10.1016/j.chemosphere.2024.143349

[CR62] Meyer, W.T., Theobald, P.K., Jr. & Bloom, H. (1979). In: P.J. Hood (Ed.), Stream Sediment Geochemistry. Geophysics and Geochemistry in the Search for Metallic Ores. *Geological Survey of Canada, Econ, Geol. Report* 31, 411-434

[CR63] Mielke, J. (1979). Composition of the Earth’s crust and distribution of the elements. In: Siegel, F.R. (Ed.), Earth Science Series No. 16. UNESCO Report SC/GEO/544/3. *Intl. Geochem. Cosmochem*. pp. 13–37.

[CR64] Najafian, T., Mokhtari, A. R., Shahrestani, S., & Albanese, S. (2023). Is the pathway length of sediments relevant to assess the background value in stream sediment geochemical exploration? *Journal of Geochemical Exploration,**253*, 107278.

[CR65] Nannoni, A., Meloni, F., Benvenuti, M., Cabassi, J., Ciani, F., Costagliola, P., Fornasaro, S., Lattanzi, P., Lazzaroni, M., Nisi, B., Morelli, G., Rimondi, V., & Vaselli, O. (2022). Environmental impact of past Hg mining activities in the Monte Amiata district, Italy: A summary of recent studies. *AIMS Geosciences,**8*, 525–551.

[CR66] Ottesen, R., & Theobald, P. (1994). Stream sediments in mineral exploration. In M. Hale & J. A. Plant (Eds.), *Drainage Geochemistry. Handbook of Exploration Geochemistry* (pp. 147–184). Elsevier.

[CR67] Pandeli, E., Bertini, G., Castellucci, P. I. E. R. O., Morelli, M., & Monechi, S. (2005). The sub-Ligurian and Ligurian units of the Mt. Amiata geothermal Region (South-eastern Tuscany): New stratigraphic and tectonic data and insight into their relationships with the Tuscan Nappe. *Bollettino della Società Geologica Italiana,**3*, 55–71.

[CR68] Pandeli, E., Bertini, G., & Orti, L. (2017). Inquadramento geologico dell’area della Monte Amiata. In C. Principe, G. Lavorini, & L. Vezzoli (Eds.), *Il Vulcano di Monte Amiata* (pp. 21–54). ESA Ed.

[CR69] Paternie, E. D. E., Hakkou, R., Nga, L. E., Oyono, L. D. B., Bessa, A. Z. E., Oubaha, S., & Khalil, A. (2023). Geochemistry and geostatistics for the assessment of trace elements contamination in soil and stream sediments in abandoned artisanal small-scale gold mining (Bétaré-Oya, Cameroon). *Applied Geochemistry,**150*, 105592.

[CR70] Pearce, J. A. (1996). A user’s guide to basalt discrimination diagrams. Trace element geochemistry of volcanic rocks: Applications for massive sulphide exploration. *Geological Association of Canada, Short Course Notes,**12*, 79–113.

[CR71] Petranich, E., Predonzani, S., Acquavita, A., Mashyanov, N., & Covelli, S. (2022). Rapid thermoscanning technique for direct analysis of mercury species in contaminated sediments: From pure compounds to real sample application. *Applied Geochemistry,**143*, 105393.

[CR72] Phillips, O. A., Falana, A. O., & Adebayo, A. J. (2017). The geochemical composition of sediment as a proxy of provenance and weathering intensity: A case study of Southwest Nigeria’s Coastal Creeks. *Geology, Geophysics & Environment,**43*(3), 229. 10.7494/geol.2017.43.3.229

[CR73] Plumlee, G. S. (1999). The environmental geology of mineral deposits. In G. S. Plimmlee & M. J. Logsdon (Eds.), *The Environmental Geochemistry of Mineral Deposits, Part A Processes, Techniques, and Health Issues* (pp. 71–116). Society of Economic Geologists.

[CR74] Protano, G., Riccobono, F. & Sabatini, G. (1998). La cartografia geochimica della Toscana meridionale. Criteri di realizzazione e rilevanza ambientale attraverso gli esempi di Hg, As, Sb, Pb e Cd. *Mem. Descr. Carta Geol. D’It*. Volume LV, 109–140. (In Italian).

[CR75] R Core Team, R. (2021). A Language and Environment for Statistical Computing. R Foundation for Statistical Computing, Vienna, Austria. Available online: https:// www.R-project.org/ (accessed on 06/06/2024)

[CR76] Rajendran, S., Rathinam, V., Sharma, A., Vallinayagam, S., & Muthusamy, M. (2024). Arsenic and environment: A systematic review on arsenic sources, uptake mechanism in plants, health hazards and remediation strategies. *Topics in Catalysis,**67*(1), 325–341. 10.1007/s11244-023-01901-9

[CR77] Raju, N. J. (2022). Arsenic in the geo-environment: A review of sources, geochemical processes, toxicity and removal technologies. *Environmental Research,**203*, 111782. 10.1016/j.envres.2021.111782 34343549 10.1016/j.envres.2021.111782

[CR78] Reiman, C., & de Caritat, P. (2017). Establishing geochemical background variation and threshold values for 59 elements in Australian surface soil. *Science of the Total Environment,**578*, 633–648. 10.1016/j.scitotenv.2016.11.010 27863868 10.1016/j.scitotenv.2016.11.010

[CR79] Reimann, C., Filzmoser, P., Hron, K., Kynclov`a, P., & Garrett, R. G. (2017). A new method for correlation analysis of compositional (environmental) data-a worked example. *Science of the Total Environment,**607*, 956–971. 10.1016/j.scitotenv.2017.06.063 10.1016/j.scitotenv.2017.06.063 28724228

[CR80] Rimondi, V., Gray, J. E., Costagliola, P., Vaselli, O., & Lattanzi, P. (2012). Concentration, distribution, and translocation of mercury and methylmercury in mine-waste, sediment, soil, water, and fish collected near the Abbadia San Salvatore mercury mine, Monte Amiata district, Italy. *Science of the Total Environment,**414*, 318–327. 10.1016/j.scitotenv.2011.10.065 22169390 10.1016/j.scitotenv.2011.10.065

[CR81] Rimondi, V., Bardelli, F., Benvenuti, M., Costagliola, P., Gray, J. E., & Lattanzi, P. (2014a). Mercury speciation in the Mt. Amiata mining district: interplay between urbanization and contamination. *Chemical Geology,**380*, 110–118.

[CR82] Rimondi, V., Costagliola, P., Gray, J. E., Lattanzi, P., Nannucci, M., Paolieri, M., & Salvadori, A. (2014b). Mass loads of dissolved and particulate mercury and other trace elements in the Mt. Amiata mining district, Southern Tuscany (Italy). *Environmental Science and Pollution Research.,**21*, 5575–5585. 24414225 10.1007/s11356-013-2476-1

[CR83] Rimondi, V., Chiaranti, L., Lattanzi, P., Benvenuti, M., Beutel, M., Colica, A., Costagliola, P., Di Bendetto, F., Gabbani, G., Gray, J. E., Pandeli, E., Pattelli, G., Paolieri, M., & Ruggieri, G. (2015). Metallogeny, exploration and environmental impact of the Mt. Amiata mercury ore district (Southern Tuscany, Italy). *Italian Journal of Geosciences,**134*, 323–336. 10.3301/IJG.2015.02

[CR84] Rimondi, V., Costagliola, P., Lattanzi, P., Morelli, G., Cara, G., Cencetti, C., Fagotti, C., Fredduzzi, A., Marchetti, G., Sconocchia, A., & Torricelli, S. (2019). A 200 km-long mercury contamination of the Paglia and Tiber floodplain: Monitoring results and implications for environmental management. *Environmental Pollution,**255*, 113191. 10.1016/j.envpol.2019.113191 31542668 10.1016/j.envpol.2019.113191

[CR86] Rose, A. W., Hawkes, H. E., & Webb, J. S. (1979). *Geochemistry in Mineral Exploration* (2nd ed.). Academic Press.

[CR87] Roser, B. P., & Korsch, R. J. (1986). Determination of tectonic setting of sandstone-mudstone suites using SiO_2_ content and K_2_O/Na_2_O ratios. *The Journal of Geology,**94*, 635–650.

[CR88] Roser, B. P., & Korsch, R. J. (1988). Provenance signatures of sandstone-mudstone suites determined using discriminant function analysis of major-element data. *Chemical Geology,**67*(1–2), 119–139.

[CR89] Rudnick, R. L., & Gao, S. (2003). Composition of the Continental Crust. In H. D. Holland & K. K. Turekian (Eds.), *Treatise on Geochemistry* (pp. 1–64). Pergamon.

[CR90] Rumayor, M., Diaz-Somoano, M., Lopez-Anton, M. A., & Martinez-Tarazona, M. R. (2013). Mercury compounds characterization by thermal desorption. *Talanta,**114*, 318–322. 10.1016/j.talanta.2013.05.059 23953477 10.1016/j.talanta.2013.05.059

[CR91] Runnels, D. D., Dupom, D. P., Jones, R. L., & Cline, D. J. (1998). Determination of natural background concentrations of dissolved components in water at mining, milling and smelting sites. *Mining Engineering,**50*, 65–71.

[CR92] Salminen, R. (Chief. Ed.), Batista, M.J., Bidovec, M., Demetriades, A., De Vivo, B., De Vos, W., Duris, M., Gilucis, A., Gregorauskiene, V., Halamic, J., Heitzmann, P., Lima, A., Jordan, G., Klaver, G., Klein, P., Lis, J., Locutura, J., Marsina, K., Mazreku, A., O’Connor, P.J., Olsson, S.Å., Ottesen, R.T., Petersell, V., Plant, J.A., Reeder, S., Salpeteur, I., Sandström, H., Siewers, U., Steenfelt, A. & Tarvainen, T. (2005). *Geochemical Atlas of Europe. Part 1 - *Background* Information, Methodology and Maps.*

[CR93] Salomao, G. N., Dall’Agnol, R., Angelica, R. S., Sahoo, P. K., & Wang, X. (2021). Geochemical mapping in stream sediments of the Carajas mineral Province, part 2: Multi-element geochemical signatures using Compositional Data Analysis (CoDA). *Journal of South American Earth Sciences,**110*, 103361. 10.1016/j.jsames.2021.103361

[CR94] Santos-Francés, F., Martinez-Grana, A. A., Alonso-Rojo, P., & Garcia-Sanchez, A. (2017). Geochemical background and baseline values determination and spatial distribution of heavy melta polution in soils of the Andes mountain range (Cajamarca-Huancavelica, Peru). *International Journal of Environmental Research and Public Health,**14*, 859. 10.3390/ijerph14080859 28788105 10.3390/ijerph14080859 PMC5580563

[CR95] Segreto, L. (1991). Monte Amiata. Il mercurio italiano. In F. Angeli (Ed.), *Strategie Internazionali e Vincoli Extra-Economici. *AbeBooks Inc.

[CR96] Singh, A., & Maichle, R. (2015). ProUCL Version 5.1 user guide. U.S. Environmental Protection Agency, Washington, DC. EPA/600/R-07/041. https://www.epa.gov/sites/production/files/2016-05/documents/proucl_5.1_user-guide.pdf.

[CR97] SNPA. (2017). Linea guida per la determinazione dei valori di fondo per i suoli e per le acque sotterranee (ISBN 978–88–448–0880–8). (In Italian).

[CR98] Stea, B. (1971). Mineralizzazioni ad antimonio della Toscana meridionale: Zolfiere o Pereta (Scansano-Grosseto). *Rend Soc Ital Mineral Petrol Fascicolo Speciale La Toscana Meridionale,**27*, 435–437.

[CR99] Stoppa, F., Rukhlov, A. S., Bell, K., Schiazza, M., & Vichi, G. (2014). Lamprophyres of Italy: Early Cretaceous alkaline lamprophyres of Southern Tuscany, Italy. *Lithos,**188*, 97–112.

[CR100] Tangari, A. C., Marinangeli, L., Scarciglia, F., Pompilio, L., & Piluso, E. (2020). Volcanic holocrystalline bedrock and hydrothermal alteration: A terrestrial analogue for Mars. *Minerals,**10*, 1082. 10.3390/min10121082

[CR101] Tassi, F., Vaselli, O., Cuccoli, F., Buccianti, A., Nisi, B., Lognoli, E., & Montegrossi, G. (2009). A geochemical multi-methodological approach in hazard assessment of CO_2_-rich gas emissions at Mt. Amiata volcano (Tuscany, central Italy). *Water, Air, Soil Pollution Focus,**9*, 117–127.

[CR102] Taylor, S. R., & McLennan, S. M. (1985). *The Continental Crust: Its Composition and Evolution* (p. 312). Blackwell.

[CR103] Vaselli, O., Lazzaroni, M., Nisi, B., Cabassi, J., Tassi, F., Rappuoli, D., & Meloni, F. (2021). Discontinuous Geochemical Monitoring of the Galleria Italia Circumneutral Waters (Former Hg-Mining Area of Abbadia San Salvatore, Tuscany, Central Italy) Feeding the Fosso Della Chiusa Creek. *Environments,**8*, 15. 10.3390/environments8020015

[CR104] Vaselli, O., Tassi, F., Nisi, B., Rappuoli, D., Pancioli, V., & Ucciero, S. (2012). CO_2_ hazard vs. touristic attraction at the Mt. Amiata volcano (Italy). *Acta Vulcanologica,**23*, 63–70.

[CR105] Zhao, W., Liu, L., Chen, J., & Ji, J. (2019). Geochemical Characterization of major elements in desert sediments and implications for the Chinese loess source. *Science China Earth Sciences,**62*, 1428–1440. 10.1007/s11430-018-9354-y

