# Correction: Provenance and distribution of potentially toxic elements (PTEs) in stream sediments from the eastern Hg-district of Mt. Amiata (central Italy)

**DOI:** 10.1007/s10653-025-02499-5

**Published:** 2025-04-23

**Authors:** Federica Meloni, Enrico Dinelli, Jacopo Cabassi, Barbara Nisi, Giordano Montegrossi, Daniele Rappuoli, Orlando Vaselli

**Affiliations:** 1 Department of Earth Sciences, Via G. Pira, 4, 50121 Florence, Italy; 2 CNR-IGG, Institute of Geosciences & Earth Resources, Via G. Pira, 4, 50121 Florence, Italy; 3 Department of Biological, Geological and Environmental Sciences, P.za Porta S. Donato, 1, 40126 Bologna, Italy; 4 INGV, Istituto Nazionale di Geofisica e Vulcanologia, Via di Vigna Murata, 605, 00143 Rome, Italy; 5 INGV, Istituto Nazionale di Geofisica e Vulcanologia, Viale Carlo Berti Pichat, 6/2, 40127 Bologna, Italy; 6 Unione dei Comuni Amiata Val d’Orcia, Unità di Bonifica, Via Grossetana 209, 53025 Siena, Piancastagnaio Italy; 7 Parco Museo Minerario di Abbadia San Salvatore, Via Suor Gemma, Abbadia San Salvatore 1, 53021 Siena, Italy

**Correction to: Environ Geochem Health (2025) 47:123** 10.1007/s10653-025-02434-8

In this article, the wrong figure appeared as Fig. 13. For completeness and transparency, both correct and incorrect versions are displayed below.

The original article has been corrected.

Incorrect version:

**Fig. 13:**
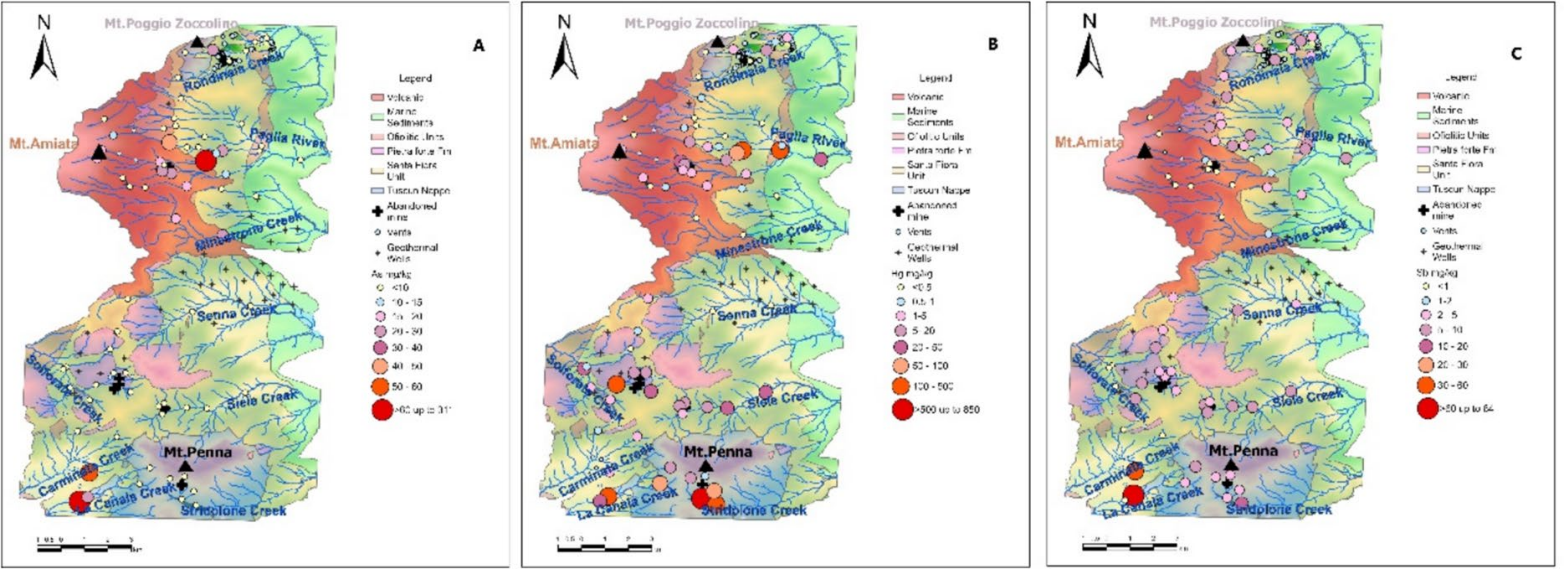
Dot-maps of As (A), Hg (B) and Sb (C) in the stream sediments (in mg/kg). See the text for further details

Correct version:

**Fig. 13:**
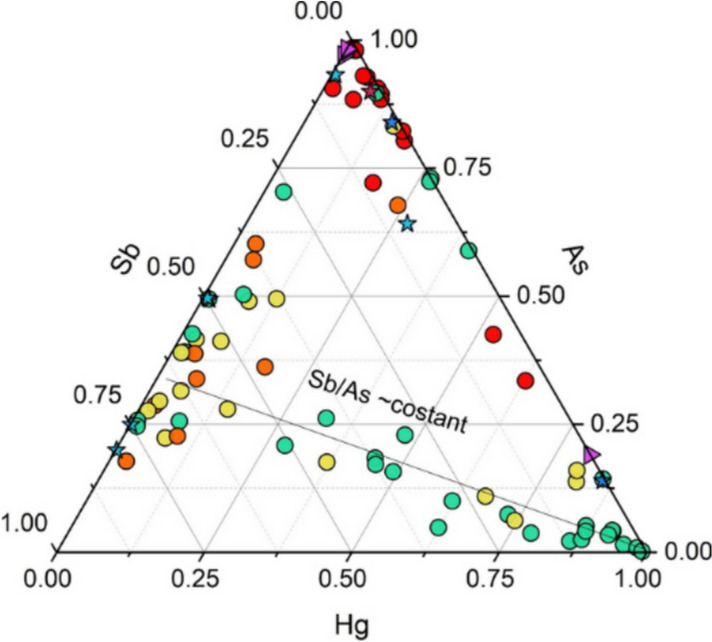
Dot-maps of As (**A**), Hg (**B**) and Sb (**C**) in the stream sediments (in mg/kg). See the text for further details

