# Supplementary material for: Provenance and distribution of potentially toxic elements (PTEs) in stream sediments from the eastern Hg-district of Mt. Amiata (central Italy)
Source: Environ Geochem Health. 2025 Mar 20;47(4):123. doi: 10.1007/s10653-025-02434-8 (PMC11925987; doi:10.1007/s10653-025-02434-8)
Supplement: Supplementary file 1 — Supplementary file1 (DOCX 38 KB) [file 10653_2025_2434_MOESM1_ESM.docx]

**Supplementary Material S1- Stream sediments XRD Results.**

Table S1: Result of XRD Semi-quantitative analysis for the stream sediments.

| **Sample** | **Quartz** | **Phillosilicate** | **K-feldspar** | **Plagioclase** | **Calcite** | **Ankerite** | **Gesso** | **Hematite** | **Dolomite** | **Gerstleyte** | **Goethite** |
| --- | --- | --- | --- | --- | --- | --- | --- | --- | --- | --- | --- |
| **STA1** | XXX | X | - | X | - | - | - | - | - | - | - |
| **STA2** | TRACCE | TRACCE | TRACCE | TRACCE | TRACCE | - | - | - | - | - | - |
| **STA3** | TRACCE | TRACCE | X | - | TRACCE | - | - | - | - | - | - |
| **STA4** | X | X | XX | X | - | - | - | - | - | - | - |
| **STA5** | X | TRACCE | TRACCE | TRACCE | - | - | - | - | - | - | - |
| **STA6** | XX | TRACCE | X | TRACCE | TRACCE | - | - | - | - | - | - |
| **STA7** | XX | TRACCE | X | - | - | - | - | - | - | - | - |
| **STA8** | TRACCE | X | XX | X | X | - | - | - | - | - | - |
| **STA9** | XX | X | - | TRACCE | X | - | - | - | - | - | - |
| **STA10** | XX | X | TRACCE | TRACCE | TRACCE | - | - | - | - | - | - |
| **STA11** | X | X | XXX | X | - | - | - | - | - | - | - |
| **STA12** | - | X | X | X | - | - | - | - | - | - | - |
| **STA13** | - | TRACCE | XXX | X | TRACCE | - | - | - | - | - | - |
| **STA14** | TRACCE | TRACCE | XXX | TRACCE | - | - | - | - | - | - | - |
| **STA15** | X | TRACCE | X | X | - | - | TRACCE | - | - | - | - |
| **STA16** | TRACCE | TRACCE | X | XXX | - | X | - | - | - | - | - |
| **STA17** | XX | X | - | TRACCE | X | - | - | - | - | - | - |
| **STA18** | XX | TRACCE | TRACCE | X | X | - | - | - | - | - | - |
| **STA19** | XXX | XXX | XXX | XX | - | - | - | - | - | - | - |
| **STA20** | XXX | XXX | - | XX | XX | - | - | - | - | - | - |
| **STA21** | XXX | X | - | - | XXX | - | - | TRACCE | - | - | - |
| **STA22** | XXX | X | - | - | XXX | - | - | TRACCE | - | - | - |
| **STA23** | XXX | X | - | - | XX | - | - | TRACCE | - | - | - |
| **STA24** | XXX | XX | - | TRACCE | XX | - | - | TRACCE | - | - | - |
| **STA25** | XXX | XXX | - | XXX | - | - | - | - | - | - | - |
| **STA26** | XXX | XX | - | XXX | XX | - | - | - | - | - | - |
| **STA27** | XXX | XX | - | - | XX | - | - | TRACCE | - | - | - |
| **STA28** | XXX | XX | - | X | XX | - | - | - | - | - | - |
| **Sample** | **Quartz** | **Phillosilicate** | **K-feldspar** | **Plagioclase** | **Calcite** | **Ankerite** | **Gesso** | **Hematite** | **Dolomite** | **Gerstleyte** | **Goethite** |
| **STA29** | XXX | XX | - | X | XX | - | - | - | - | - | - |
| **STA30** | XXX | XX | - | - | XX | - | - | - | - | - | - |
| **STA31** | XXX | X | - | XX | X | - | - | - | XX | - | - |
| **STA32** | XX | XX | XX | X | - | - | - | - | - | - | - |
| **STA33** | XXX | X | - | XX | - | - | - | - | - | - | - |
| **STA34** | XXX | XXX | - | XXX | XXX | - | - | - | - | - | - |
| **STA35** | XX | XXX | XXX | X | XXX | - | - | - | - | - | - |
| **STA36** | XXX | XXX | - | X | XXX | - | - | - | X | - | - |
| **STA37** | XXX | XX | - | X | XX | - | - | - | - | - | - |
| **STA38** | XXX | XX | - | X | XX | - | - | - | - | - | - |
| **STA39** | XXX | XX | - | X | XX | - | - | - | - | - | - |
| **STA40** | XXX | XX | - | X | XX | - | - | - | - | - | - |
| **STA41** | XXX | XX | - | X | XX | - | XX | - | - | - | - |
| **STA42** | XXX | XX | - | TRACE | XX | - | - | TRACE | - | - | - |
| **STA43** | XXX | XX | - | X | XX | - | - | - | - | - | - |
| **STA44** | XXX | XX | - | X | XX | - | - | - | - | - | - |
| **STA45** | XXX | XX | - | TRACE | XX | - | - | - | - | - | - |
| **STA46** | XXX | X | - | TRACE | X | - | - | TRACCE | - | - | - |
| **STA47** | XXX | X | - | TRACE | X | - | - | TRACE | - | - | - |
| **STA48** | XXX | XX | - | X | X | - | - | - | - | - | - |
| **STA49** | XXX | XX | - | TRACE | XX | - | - | - | - | - | - |
| **STA50** | XXX | X | - | TRACE | X | - | - | - | - | - | - |
| **STA51** | XXX | XX | - | XX | XX | - | - | - | - | - | - |
| **STA52** | XXX | XX | - | - | XX | - | - | - | - | - | - |
| **STA53** | XXX | XX | - | X | XX | - | - | - | - | - | - |
| **STA54** | XXX | XX | - | TRACE | XXX | - | - | - | - | - | - |
| **MSIE-16** | XXX | X | - | - | XX | - | - | - | - | - | - |
| **MSIE-04** | XXX | X | - | - | XXX | - | - | - | - | - | - |
| **Sie 189** | XXX | XX | - | - | XX | - | - | - | - | - | - |
| **SIE Br2** | XXX | XX | - | X | XX | - | - | - | - | - | - |
| **MSIE 05** | XXX | XX | - | - | XXX | - | - | - | - | - | - |
| **Sample** | **Quartz** | **Phillosilicate** | **K-feldspar** | **Plagioclase** | **Calcite** | **Ankerite** | **Gesso** | **Hematite** | **Dolomite** | **Gerstleyte** | **Goethite** |
| **Msie 11** | XXX | XX | - | - | X | - | - | - | - | X | - |
| **PA01** | XX | TRACCE | X | - | X | - | - | - | - | - | - |
| **GIT12** | XX | TRACCE | X | - | X | - | - | - | - | - | - |
| **GIT4** | XX | - | X | - | X | - | - | - | - | - | X |
| **GIT0** | - | X | - | - | - | - | - | - | - | - | TRACE |
| **9189A22** | XXX | XX | - | X | XX | - | - | - | - | - | - |
| **9171A22** | XX | X | - | - | XXX | - | - | TRACE | - | - | - |
| **9080A22** | XXX | XX | - | X | X | - | - | - | - | - | - |
| **9081A22** | XXX | XX | - | X | XX | - | - | - | - | - | - |
| **9083A22** | XXX | XX | - | TRACE | XXX | - | - | TRACE | - | - | - |
| **9211A22** | XXX | XX | - | X | XX | - | - | - | - | - | - |
| **9163A22** | XXX | XX | - | X | XXX | - | - | - | - | - | - |
| **Salto1** | XXX | XX | - | - | XX | - | X | TRACE | X | - | - |
| **Salto2** | XXX | XX | - | TRACE | X | - | - | TRACE | - | - | - |
| **Salto3** | XXX | XX | - | X | XXX | - | XX | - | XX | - | - |
| **Salto4** | XXX | XX | - | - | XX | - | - | TRACE | XX | - | - |
| **Lake Green** | XXX | XXX | XXX | - | X | - | - | - | - | - | - |
