# Supplementary material for: Provenance and distribution of potentially toxic elements (PTEs) in stream sediments from the eastern Hg-district of Mt. Amiata (central Italy)
Source: Environ Geochem Health. 2025 Mar 20;47(4):123. doi: 10.1007/s10653-025-02434-8 (PMC11925987; doi:10.1007/s10653-025-02434-8)
Supplement: Supplementary file 2 — Supplementary file2 (DOCX 4288 KB) [file 10653_2025_2434_MOESM2_ESM.docx]

**Supplementary material S2 - Petrographic observations**

**Rock sample APA**


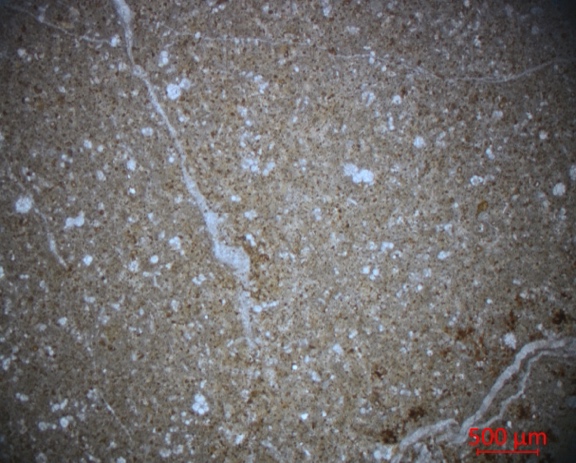

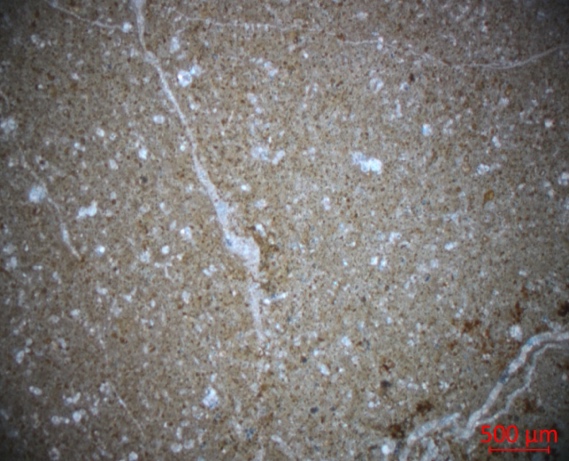


A

C

B


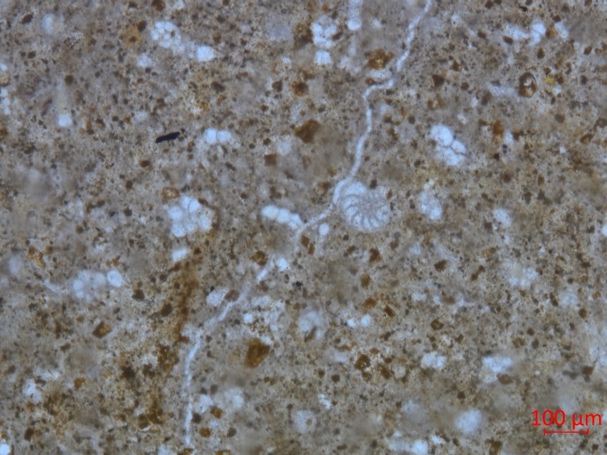


Figure 1: Sample APA. A) thin section at parallel and B) crossed nicols; C) detail a calcified ammonite and other organic fragments can be observed.

The sample APA can be classified as a biomicrite (Folk, 1959) or mudstone bioclastite for Dunham (1962) with spicules, calcified radiolarians, small foraminifera and ammonites (see Figure C). The section is cut by veins of very fine calcite. The matrix appears to be a brown microcrystalline calcitic matrix with quartz crystals scattered within. Oxides are scattered in the matrix.

**Rock Sample SFR 1**

A

B


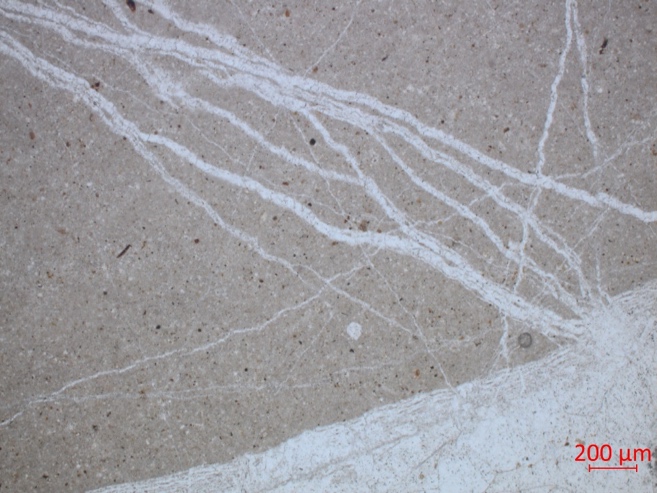

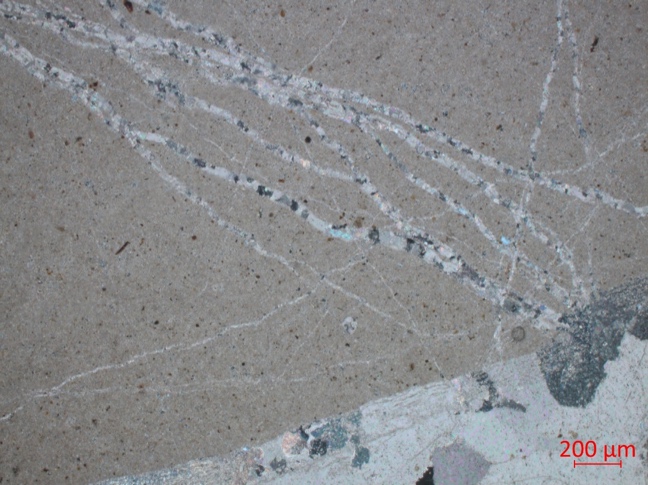


Cc

Figure 2: Sample SFR 1 A) photograph of parallel and B) crossed nicols..Cc: Calcite

Sample SFR 1 can be classified as a biomicrite (Folk, 1959) or a mudstone bioclastite (Dunham, 1962). In this case, the sample is characterised but a higher calcite contribution. Calcite veins running through the carbonate matrix where small quartz crystals can be found.

**Rock Sample SFR 2**


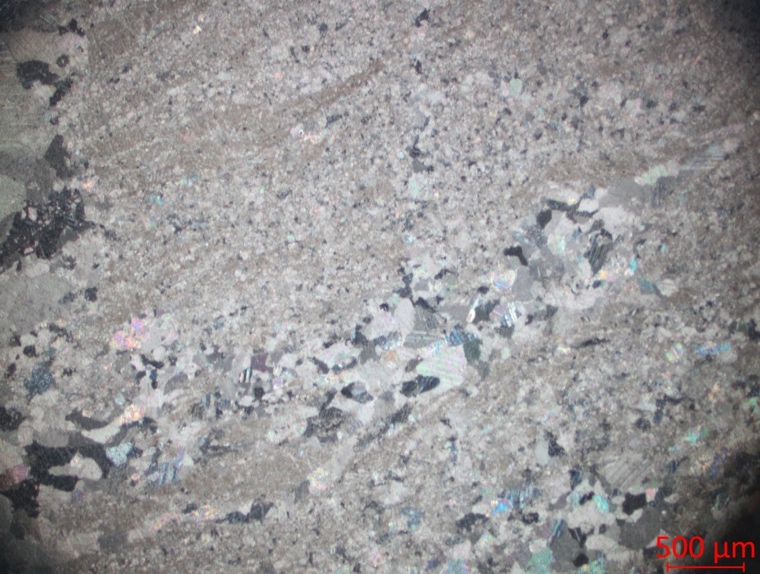


Figure 3: : Sample SFR 2 photograph of parallel crossed nicols

This sample can be classified as wackestone, as the calcite clast content has increased and quartz clasts are visible. Numerous calcite veins are present with different dimensions. The matrix is always micritic calcitic with scattered quartz. Small recrystallised fragments of foraminifera are always present.

**Rock Sample SFR 3**

A

B


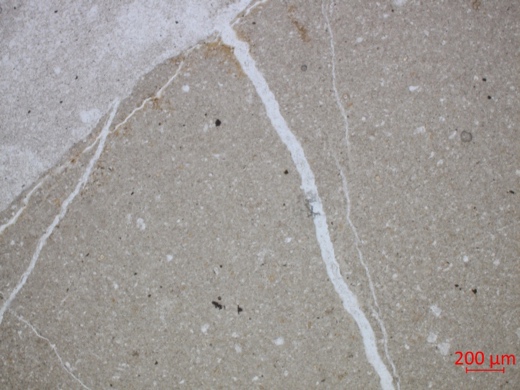

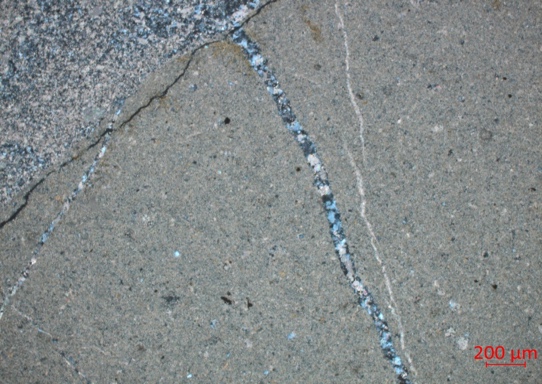


Figure 4: : Sample SFR 3 A) photograph of parallel and B) crossed nicols.

Mudstone with alternating veins of calcite and quartz crystals with different dimensions. Foraminifers and iron oxides are present in the microcrystalline calcite matrix.

**Rock Sample SFR 4**

A

B


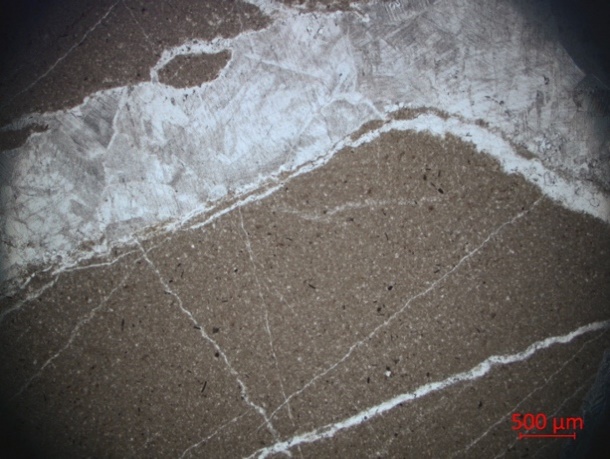

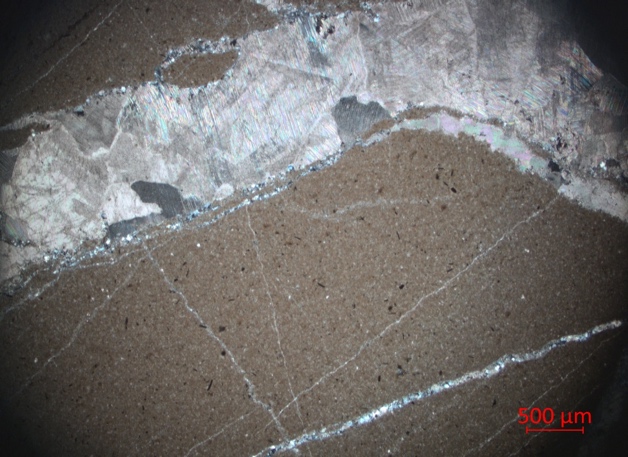


Figure 5: : Sample SFR 4 A) photograph of parallel and B) crossed nicols

Mudstone with veins of well-crystallised calcite, muscovite and albite running through it. The calcite crystals are in contact with scattered quartz agglomerates. The matrix is made of calcite and microcrystalline quartz. Iron oxides in the matrix and small microfossils occur.

**Rock sample PTF**

B

A


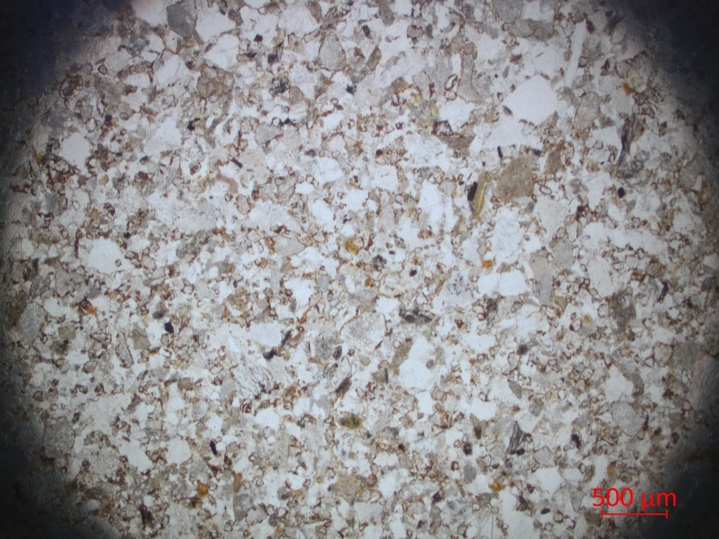

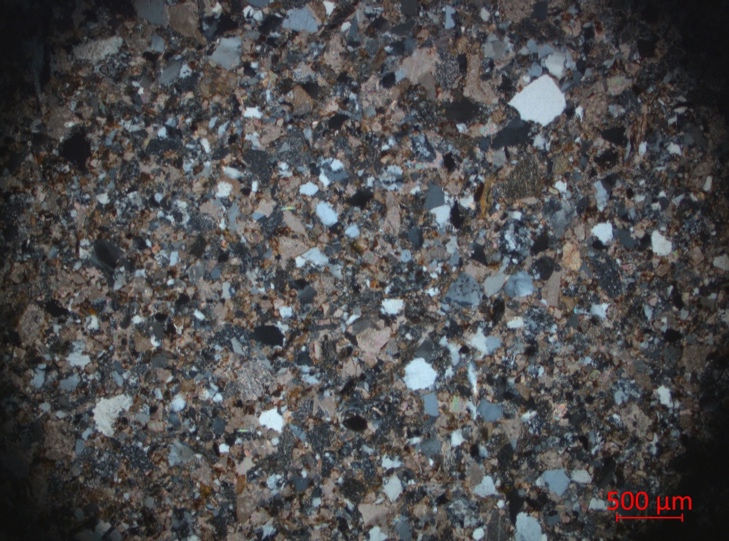


Kf

cc

Qz

Figura 6: : Sample PTF A) photograph of parallel and B) crossed nicols

Feldspatic-arcose. The clasts are in contact each other in a sustained grain texture. We find a minimum of a quartz phyllosilicate matrix and a quartzitic cement. The main grains are quartz with wavy extinction, both geminated and ungrown k-feldspars, plagioclase with polysynthetic gemination and albite-Carlsbad. Femic minerals (biotite and muscovite) and micritic limestones are observed. Fragments of metamorphic rocks such as quartzites characterised by striated quartz, chlorite and goethite are also found. Zircon as accessory mineral.

**Rock sample Burano**


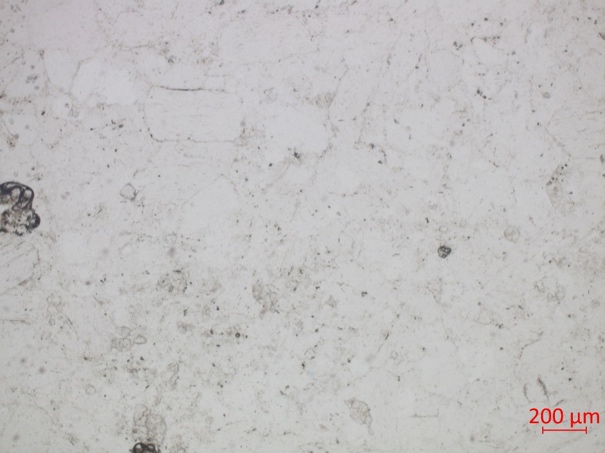

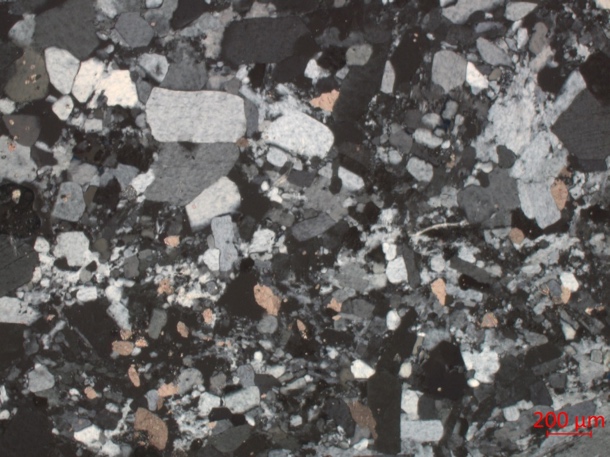


Cc

B

A

Figure 7: : Sample Burano A) photograph of parallel and B) crossed nicols. Cc: calcite

Prismatic chalk crystals (black to white in colour) with calcite and a few calcite clasts scattered in the matrix of various sizes. Zircon as accessory mineral.

**Rock sample Serpentinite**


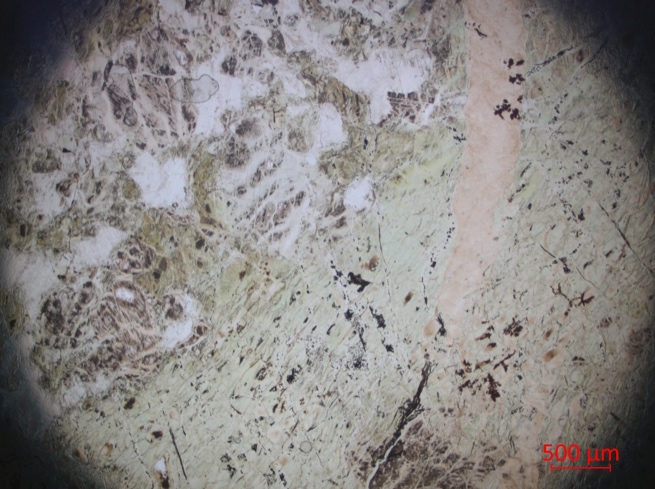

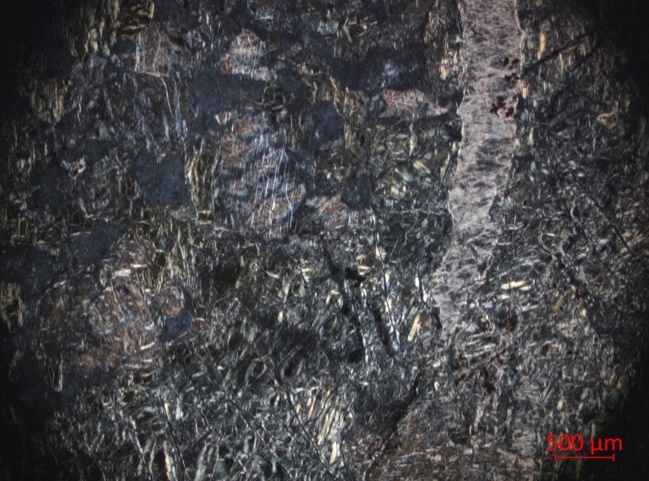


B

A

talc

Figure 8: : Sample Serpentine A) photograph of parallel and B) crossed nicols.

The serpentinite specimen shows a felt structure with lizardite veins and sometimes larger or smaller talc veins are present. Scattered in the matrix are Fe oxides and ilmenite. In parallel nicols (Fig. 8A) the sample appears to be covered by a patina of chlorite that gives the rock its classic green colour.

**Rock sample OLF1**

B


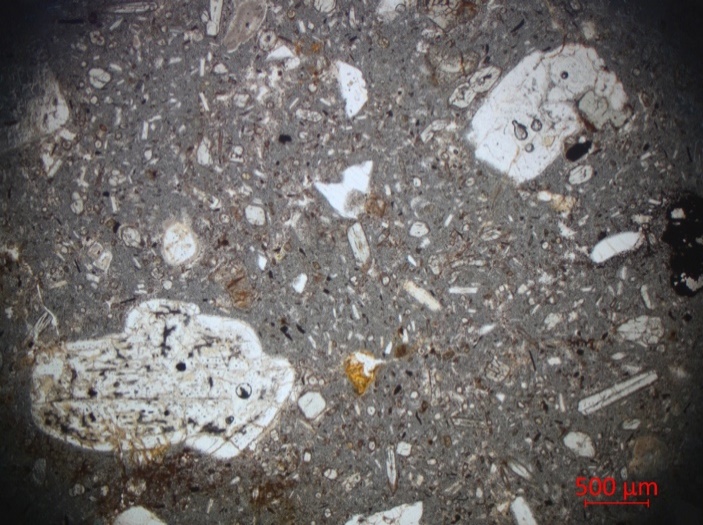

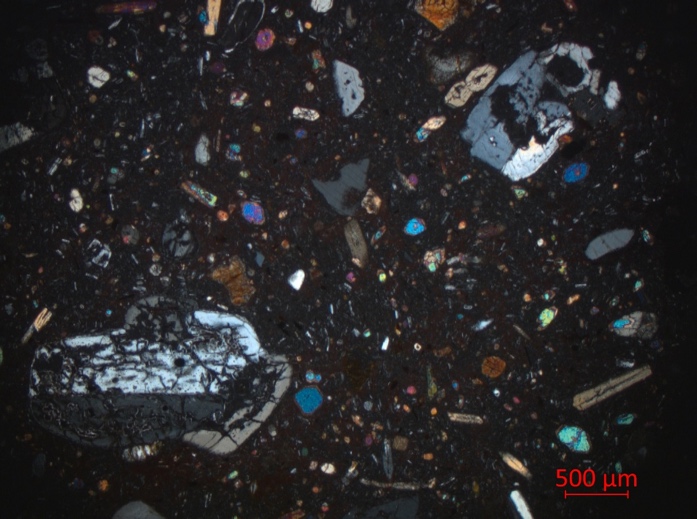


opx

kf

m

Ol

cpx

A

Figure 9: Sample OLF1 A) photograph of parallel and B) crossed nicols. Kf: K-feldspar; opx: orto-pyroxene; m: muscovite; Ol: olivine: cpx: clinopyroxene

Hypohaline volcanic rock from Ol-latitic lavas with porphyritic, aphanitic texture. Olivine are in the microcrystalline groundmass with plagioclase, k-feldspar, quartz and clinopyroxene. The rock cement is characterised by glass. Carlsbad and zoned plagioclase, biotite and muscovite are present. Orthopyroxene is found in the matrix and also within plagioclase. Clinopyroxene is abundant and often characterised by an iron oxide alteration rim. Zircon as accessory mineral.

**Rock sample Vulc**

B


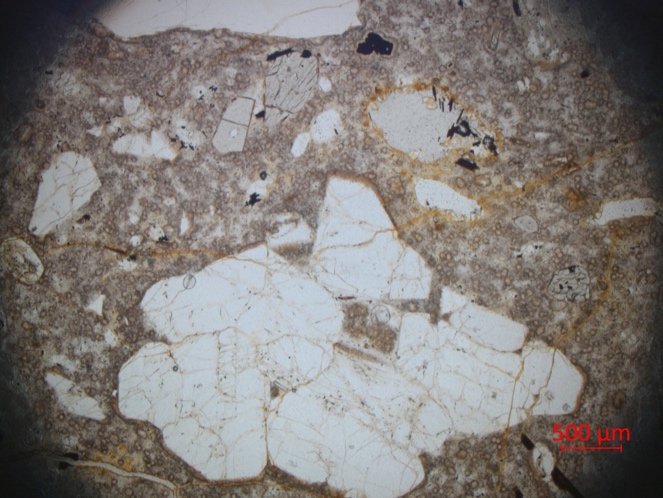

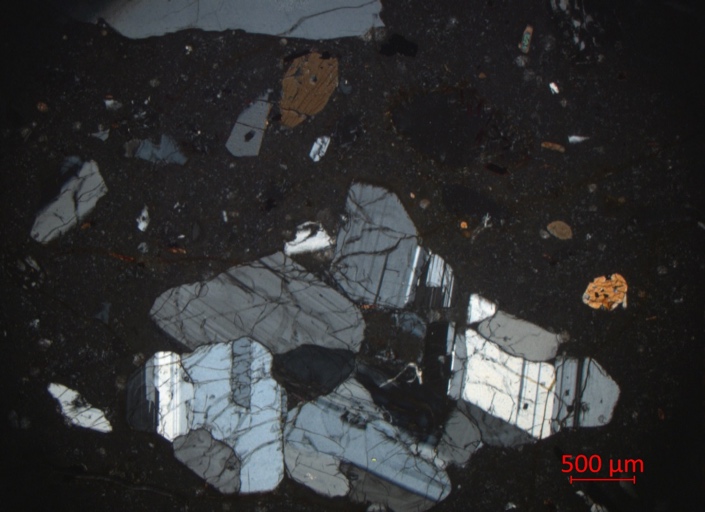


A

Figure 10: Sample VULC A) photograph of parallel and B) crossed nicols.

Hypohaline volcanic rock with porphyritic, aphanitic texture. The Vulc rock has a glassy perlitic texture. Large k-feldspars (sanidine) and plagioclase are present. Orthopyroxene often contains quartz inclusions, while biotite can be found in plagioclase. Iron oxides and ilmenite are present.

**Rock sample QRT**


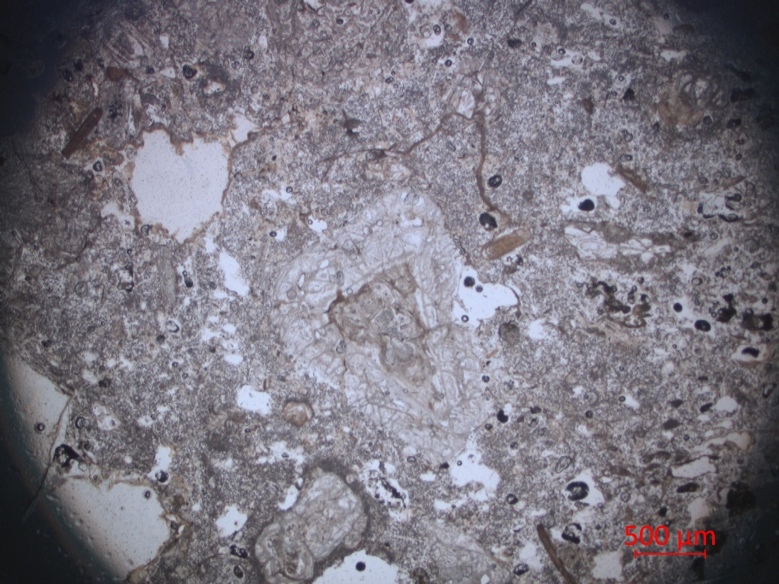

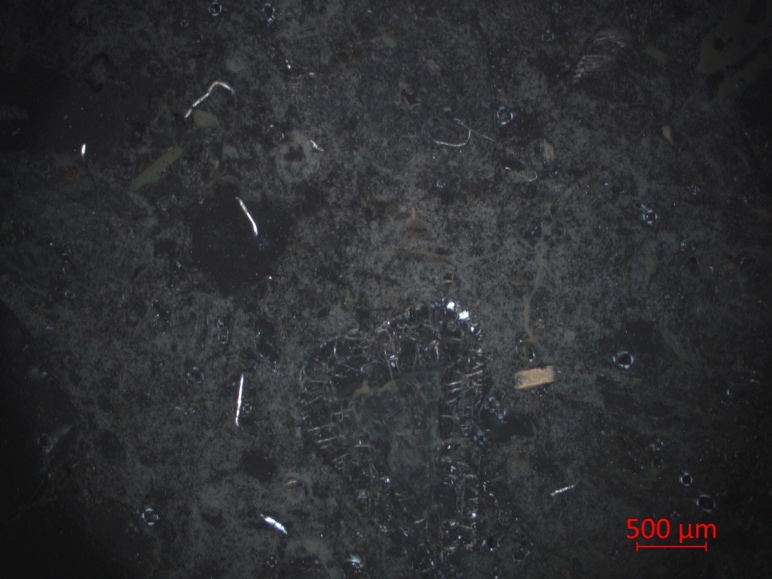


A

B

Figura 11: Sample QRT A) photograph of parallel and B) crossed nicols.

QRT is as an altered rock with a large presence of voids, with relics of olivine and orthopyroxene crystals. A few k-feldspars (sanidine) can be observed. The groundmass is glassy. Biotite crystals are present.

**Rock sample Filone**


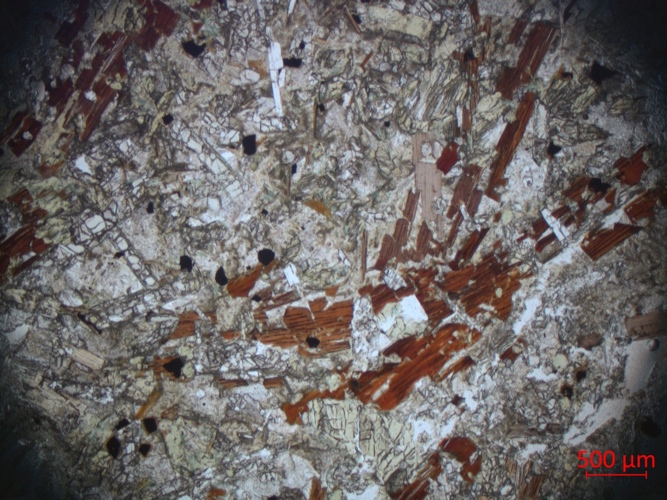

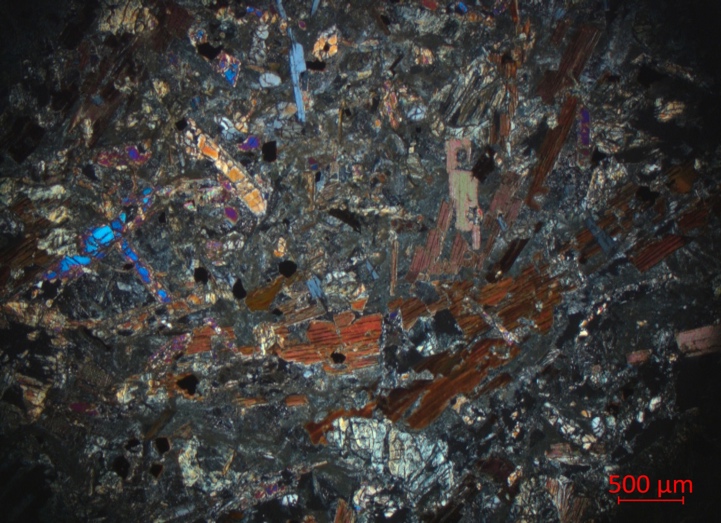


A

B


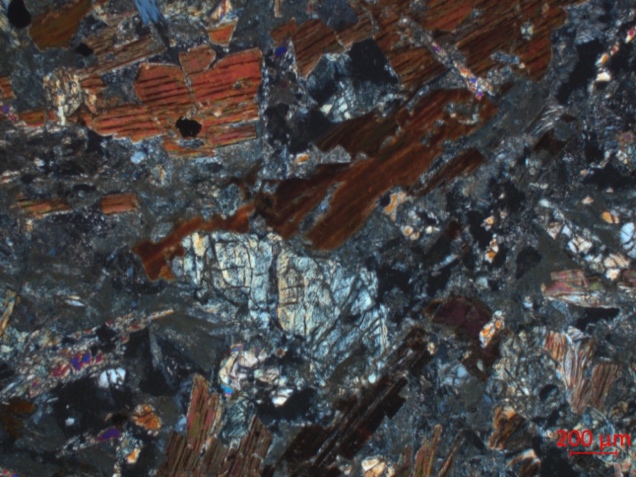


C

Figure 12: Sample Filone A) photograph of parallel and B) crossed nicols.; C) detail of serpentinized olivine

The Filone sample at parallel nicols (Fig. 12A) consists of mafic minerals and chlorite with high content of biotite and muscovite, orthopyroxene, and (serpentinized) olivine (detail in Fig. 12C). The matrix is mostly serpentine, indicative of hydrothermal metamorphism, and glass. Apatite and iron oxides are present.

**Rock Sample altered Filone**


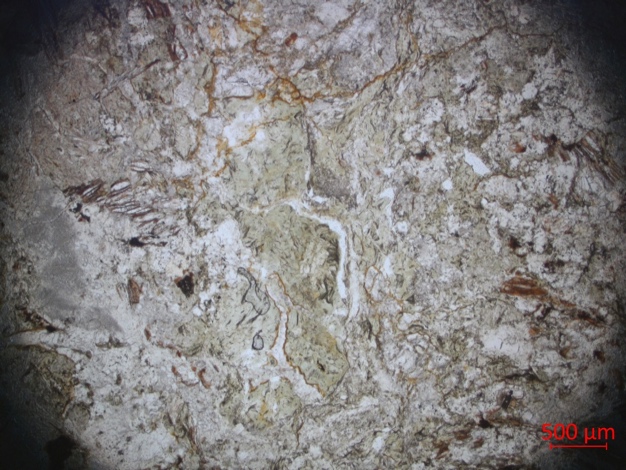

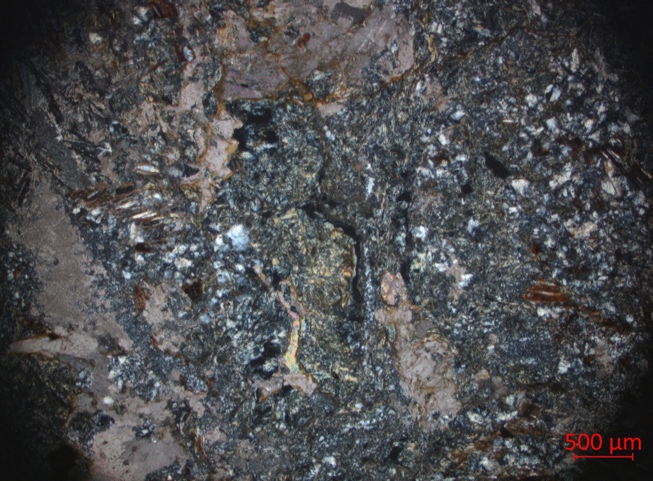


B

A

Figure 13: Sample Filone A) photograph of parallel and B) crossed nicols.

This rock is the altered equivalent of Filone. The serpentine content has increased, and the pyroxene and olivine clasts are no longer visible, being completely serpentinized. Calcite is found in both the groundmass (mostly serpentine and glass) and as individual crystals. Biotite and muscovite crystals are still present, and they are also partially serpentinized. Additionally, clasts of quartz, plagioclase, chlorite and iron oxides are present.

**References**

Dunham, R. J. (1962). Classification of carbonate rocks according to depositional textures.

Folk, R.L. (1959). Practical Petrographic Classification of Limestones. AAPG Bulletin. 43 (1): 1–38. doi:10.1306/0BDA5C36-16BD-11D7-8645000102C1865D
