# Supplementary material for: Provenance and distribution of potentially toxic elements (PTEs) in stream sediments from the eastern Hg-district of Mt. Amiata (central Italy)
Source: Environ Geochem Health. 2025 Mar 20;47(4):123. doi: 10.1007/s10653-025-02434-8 (PMC11925987; doi:10.1007/s10653-025-02434-8)
Supplement: Supplementary file 3 — Supplementary file3 (DOCX 71 KB) [file 10653_2025_2434_MOESM3_ESM.docx]

**Supplementary Materials S2- Results of the XRF analysis (major and trace elements) and aqua regia extraction of the stream sediments**

Table S2.1: Geographic coordinates in UTM 32N WGS 84, major elements as oxides and expressed in wt % analyzed by XRF analysis and LOI in % in stream sediments

| **Sample** | **X** | **Y** | **SiO_2_** | **TiO_2_** | **Al_2_O_3_** | **Fe_2_O_3_** | **MnO** | **MgO** | **CaO** | **Na_2_O** | **K_2_O** | **P_2_O_5_** | **LOI** |
| --- | --- | --- | --- | --- | --- | --- | --- | --- | --- | --- | --- | --- | --- |
| **STA01** | **718052** | **4750343** | 56.2 | 0.59 | 21.3 | 2.95 | 0.06 | 0.95 | 2.00 | 1.15 | 5.14 | 0.25 | 9.34 |
| **STA02** | **719259** | **4750888** | 51.8 | 0.71 | 18.6 | 4.68 | 0.21 | 1.41 | 6.68 | 0.92 | 5.36 | 0.33 | 9.32 |
| **STA03** | **717273** | **4750985** | 59.8 | 0.62 | 19.8 | 3.62 | 0.08 | 0.99 | 1.58 | 1.35 | 6.46 | 0.16 | 5.54 |
| **STA04** | **717187** | **4751354** | 52.2 | 0.80 | 18.6 | 4.63 | 0.11 | 1.24 | 6.56 | 0.92 | 4.91 | 0.23 | 9.80 |
| **STA05** | **717452** | **4752192** | 56.8 | 0.72 | 19.4 | 7.47 | 0.25 | 1.16 | 1.37 | 0.76 | 4.26 | 0.20 | 7.59 |
| **STA06** | **717626** | **4752892** | 55.6 | 0.73 | 18.8 | 5.13 | 0.13 | 1.41 | 2.20 | 0.91 | 3.96 | 0.16 | 11.04 |
| **STA07** | **717261** | **4752774** | 54.7 | 0.95 | 21.3 | 5.99 | 0.07 | 1.56 | 1.22 | 0.66 | 3.07 | 0.18 | 10.34 |
| **STA08** | **718940** | **4751767** | 48.7 | 0.60 | 14.4 | 5.69 | 0.09 | 1.58 | 12.36 | 0.82 | 4.81 | 0.18 | 10.77 |
| **STA09** | **718371** | **4752015** | 50.5 | 0.83 | 22.3 | 7.17 | 0.05 | 2.49 | 2.64 | 0.73 | 3.98 | 0.15 | 9.16 |
| **STA10** | **718550** | **4752460** | 55.9 | 0.84 | 19.7 | 6.92 | 0.13 | 2.33 | 1.92 | 0.78 | 3.65 | 0.11 | 7.72 |
| **STA11** | **715691** | **4752433** | 59.1 | 0.73 | 17.6 | 6.34 | 0.12 | 1.56 | 2.08 | 1.41 | 7.18 | 0.22 | 3.69 |
| **STA12** | **715330** | **4751784** | 55.1 | 0.68 | 19.4 | 5.15 | 0.12 | 1.35 | 2.13 | 1.07 | 5.94 | 0.24 | 8.84 |
| **STA13** | **715207** | **4751443** | 60.9 | 0.54 | 17.8 | 3.74 | 0.07 | 0.72 | 1.52 | 1.30 | 6.42 | 0.15 | 6.79 |
| **STA14** | **716041** | **4750629** | 58.5 | 1.03 | 17.5 | 7.58 | 0.14 | 2.00 | 1.63 | 1.32 | 6.78 | 0.25 | 3.21 |
| **STA15** | **716276** | **4750273** | 59.7 | 1.07 | 16.3 | 8.05 | 0.15 | 1.98 | 1.97 | 1.46 | 6.45 | 0.21 | 2.69 |
| **STA16** | **717549** | **4750891** | 58.7 | 0.72 | 20.1 | 3.94 | 0.07 | 1.09 | 1.47 | 1.29 | 6.24 | 0.18 | 6.16 |
| **STA17** | **720237** | **4749997** | 45.5 | 0.67 | 16.8 | 6.44 | 0.10 | 2.32 | 9.37 | 0.52 | 3.26 | 0.11 | 14.94 |
| **STA18** | **719147** | **4750262** | 42.0 | 0.61 | 15.9 | 5.59 | 0.07 | 2.20 | 10.20 | 0.49 | 3.50 | 0.13 | 19.35 |
| **STA19** | **716760** | **4750250** | 62.5 | 0.76 | 18.1 | 3.86 | 0.08 | 0.97 | 1.70 | 1.55 | 6.72 | 0.15 | 3.59 |
| **STA20** | **718012** | **4751997** | 50.0 | 0.91 | 21.9 | 7.14 | 0.08 | 2.42 | 3.02 | 0.70 | 3.92 | 0.15 | 9.74 |
| **STA21** | **716569** | **4741570** | 40.0 | 0.57 | 13.4 | 9.59 | 0.28 | 0.75 | 10.75 | 0.31 | 1.20 | 0.10 | 23.07 |
| **STA22** | **715480** | **4741839** | 44.4 | 0.53 | 13.6 | 11.60 | 0.24 | 1.73 | 10.13 | 0.32 | 1.72 | 0.11 | 15.56 |
| **STA23** | **716387** | **4742377** | 50.9 | 0.73 | 16.4 | 10.09 | 0.34 | 1.46 | 3.60 | 0.35 | 2.53 | 0.11 | 13.49 |
| **STA24** | **716018** | **4742358** | 48.6 | 0.71 | 16.9 | 11.23 | 0.45 | 1.29 | 4.20 | 0.45 | 2.25 | 0.11 | 13.81 |
| **STA25** | **716354** | **4745512** | 52.0 | 0.95 | 19.6 | 7.99 | 0.17 | 2.08 | 2.89 | 0.64 | 3.52 | 0.17 | 10.05 |
| **STA26** | **716076** | **4744133** | 47.3 | 0.84 | 18.7 | 7.03 | 0.09 | 2.23 | 4.11 | 0.52 | 3.40 | 0.11 | 15.68 |
| **STA27** | **717643** | **4754954** | 48.3 | 0.81 | 18.2 | 6.86 | 0.14 | 1.61 | 6.76 | 0.55 | 2.08 | 0.24 | 14.45 |
| **STA28** | **717831** | **4756025** | 51.5 | 0.80 | 17.5 | 8.12 | 0.33 | 1.74 | 4.16 | 0.53 | 2.15 | 0.21 | 13.02 |
| **STA29** | **720001** | **4756534** | 48.4 | 0.76 | 18.2 | 7.17 | 0.12 | 2.52 | 6.26 | 0.58 | 3.33 | 0.11 | 12.62 |
| **STA30** | **719730** | **4756151** | 51.0 | 0.75 | 18.0 | 7.38 | 0.15 | 2.33 | 5.82 | 0.61 | 2.90 | 0.11 | 10.94 |
| **STA31** | **718692** | **4756159** | 40.7 | 0.47 | 12.4 | 7.28 | 0.22 | 1.58 | 13.53 | 0.25 | 1.98 | 0.13 | 21.52 |
| **STA32** | **715385** | **4753122** | 59.5 | 0.77 | 18.4 | 5.36 | 0.11 | 1.40 | 1.95 | 1.40 | 7.59 | 0.20 | 3.32 |
| **STA33** | **717759** | **4754069** | 52.7 | 0.86 | 19.3 | 7.45 | 0.14 | 2.21 | 3.45 | 0.68 | 2.77 | 0.13 | 10.37 |
| **STA34** | **717564** | **4753211** | 57.0 | 0.91 | 20.1 | 6.65 | 0.17 | 1.73 | 1.63 | 0.84 | 3.33 | 0.17 | 7.45 |
| **STA35** | **719247** | **4748299** | 59.2 | 0.77 | 20.6 | 3.80 | 0.07 | 0.97 | 2.10 | 1.30 | 6.92 | 0.16 | 4.05 |
| **STA36** | **717762** | **4748958** | 57.2 | 1.18 | 17.6 | 7.81 | 0.14 | 1.98 | 1.83 | 1.30 | 7.29 | 0.21 | 3.46 |
| **STA37** | **718399** | **4745035** | 49.9 | 0.86 | 19.0 | 7.04 | 0.09 | 2.36 | 3.92 | 0.65 | 3.48 | 0.11 | 12.52 |
| **STA38** | **720216** | **4745347** | 48.2 | 0.82 | 17.5 | 7.28 | 0.10 | 2.53 | 6.18 | 0.65 | 3.18 | 0.12 | 13.45 |
| **STA39** | **720356** | **4755887** | 47.4 | 0.77 | 18.6 | 7.29 | 0.12 | 2.84 | 5.87 | 0.57 | 3.71 | 0.11 | 12.65 |
| **STA40** | **720359** | **4756718** | 47.9 | 0.71 | 17.1 | 6.50 | 0.13 | 1.95 | 6.54 | 0.52 | 2.86 | 0.11 | 15.65 |
| **STA41** | **719221** | **4755690** | 53.1 | 0.84 | 20.0 | 6.01 | 0.04 | 2.13 | 1.45 | 0.70 | 3.35 | 0.12 | 12.22 |
| **STA42** | **715875** | **4743368** | 48.1 | 0.77 | 17.5 | 11.27 | 0.33 | 1.54 | 3.13 | 0.44 | 3.08 | 0.10 | 13.81 |
| **STA43** | **719688** | **4753056** | 53.3 | 0.84 | 20.1 | 6.97 | 0.14 | 2.49 | 2.49 | 0.69 | 3.55 | 0.12 | 9.32 |
| **STA44** | **721579** | **4751582** | 46.9 | 0.72 | 17.8 | 6.43 | 0.08 | 2.44 | 8.39 | 0.54 | 3.31 | 0.13 | 13.23 |
| **STA45** | **715307** | **4741182** | 51.2 | 0.90 | 18.2 | 7.80 | 0.13 | 1.01 | 3.80 | 0.31 | 1.79 | 0.09 | 14.80 |
| **STA46** | **718771** | **4736827** | 61.0 | 0.50 | 12.2 | 7.40 | 0.58 | 1.43 | 4.75 | 0.28 | 1.62 | 0.15 | 10.09 |
| **STA47** | **718317** | **4737070** | 60.7 | 0.50 | 12.6 | 6.37 | 0.51 | 1.44 | 5.13 | 0.30 | 1.83 | 0.15 | 10.50 |
| **STA48** | **718382** | **4738028** | 42.8 | 0.65 | 15.0 | 6.22 | 0.37 | 2.09 | 8.15 | 0.40 | 2.14 | 0.18 | 21.98 |
| **STA49** | **717925** | **4737928** | 45.4 | 0.80 | 16.9 | 6.49 | 0.19 | 2.12 | 7.10 | 0.35 | 1.87 | 0.20 | 18.58 |
| **STA50** | **718688** | **4737413** | 48.7 | 0.93 | 18.8 | 6.94 | 0.30 | 2.15 | 1.14 | 0.36 | 2.12 | 0.18 | 18.42 |
| **STA51** | **715383** | **4738108** | 55.0 | 0.37 | 11.5 | 9.34 | 0.29 | 0.78 | 5.70 | 0.17 | 1.55 | 0.16 | 15.07 |
| **STA52** | **716978** | **4737666** | 44.4 | 0.74 | 18.1 | 6.84 | 0.12 | 1.04 | 8.55 | 0.53 | 1.65 | 0.15 | 17.94 |
| **STA53** | **715369** | **4737071** | 37.5 | 0.41 | 11.8 | 6.42 | 0.15 | 1.38 | 15.88 | 0.32 | 1.40 | 0.14 | 24.63 |
| **STA54** | **717277** | **4738358** | 50.3 | 0.84 | 17.3 | 7.14 | 0.43 | 2.55 | 1.18 | 0.41 | 2.22 | 0.18 | 17.47 |
| **9080A22** | **714516** | **4742562** | 48.5 | 0.84 | 19.0 | 8.51 | 0.15 | 2.12 | 4.06 | 0.55 | 3.30 | 0.10 | 12.93 |
| **9081A22** | **714436** | **4742475** | 45.7 | 0.60 | 15.1 | 8.02 | 0.15 | 2.25 | 10.22 | 0.43 | 2.45 | 0.11 | 14.94 |
| **9083A22** | **714666** | **4741679** | 45.9 | 0.71 | 18.0 | 9.22 | 0.12 | 1.46 | 5.34 | 0.41 | 2.58 | 0.10 | 16.22 |
| **9163A22** | **714966** | **4739854** | 48.8 | 0.70 | 17.7 | 8.00 | 0.16 | 1.74 | 3.53 | 0.55 | 2.47 | 0.09 | 16.24 |
| **9171A22** | **715116** | **4736858** | 39.8 | 0.37 | 11.3 | 5.90 | 0.13 | 1.37 | 18.55 | 0.32 | 1.46 | 0.15 | 20.66 |
| **9211A22** | **716201** | **4739649** | 45.1 | 0.66 | 15.6 | 10.50 | 0.24 | 1.67 | 4.65 | 0.45 | 1.90 | 0.10 | 19.12 |
| **GIT04** | **719124** | **4751887** | 34.3 | 0.58 | 27.2 | 10.36 | 0.15 | 0.77 | 2.76 | 0.36 | 2.03 | 0.54 | 20.91 |
| **GIT12** | **720292** | **4751930** | 49.4 | 0.86 | 19.9 | 7.16 | 0.15 | 2.18 | 4.87 | 0.59 | 3.57 | 0.22 | 11.12 |
| **Green Lake** | **717319** | **4751227** | 57.8 | 0.71 | 19.1 | 3.66 | 0.06 | 1.10 | 3.59 | 1.13 | 5.76 | 0.17 | 6.86 |
| **MSIE11** | **717588** | **4740665** | 49.2 | 0.82 | 18.4 | 8.41 | 0.20 | 1.23 | 4.82 | 0.62 | 1.73 | 0.14 | 14.40 |
| **MSIE16** | **718359** | **4740961** | 48.7 | 0.93 | 20.4 | 6.50 | 0.11 | 1.64 | 4.79 | 0.68 | 1.95 | 0.17 | 14.11 |
| **MSIE05** | **717640** | **4741128** | 37.6 | 0.56 | 14.3 | 5.72 | 0.08 | 0.73 | 17.30 | 0.35 | 1.30 | 0.11 | 21.94 |
| **PA-1** | **720345** | **4752129** | 50.9 | 0.89 | 20.5 | 6.47 | 0.11 | 2.54 | 3.98 | 0.67 | 3.64 | 0.18 | 10.20 |
| **SALTO1** | **726203** | **4742176** | 42.5 | 0.69 | 16.7 | 5.24 | 0.10 | 2.06 | 11.17 | 0.36 | 1.66 | 0.11 | 19.38 |
| **SALTO2** | **726203** | **4742176** | 45.7 | 0.93 | 20.1 | 6.18 | 0.06 | 0.88 | 5.50 | 0.30 | 1.51 | 0.11 | 18.81 |
| **SALTO3** | **726203** | **4742176** | 47.6 | 0.82 | 19.1 | 5.76 | 0.10 | 4.69 | 7.87 | 0.23 | 2.23 | 0.14 | 11.54 |
| **SALTO4** | **726203** | **4742176** | 42.8 | 0.68 | 16.7 | 4.77 | 0.07 | 2.60 | 8.95 | 0.25 | 1.83 | 0.12 | 21.21 |
| **SIE BR2** | **718984** | **4740941** | 46.9 | 0.77 | 17.5 | 6.83 | 0.12 | 1.78 | 8.48 | 0.55 | 2.14 | 0.18 | 14.71 |
| **SIE189** | **720169** | **4741653** | 47.6 | 0.81 | 19.0 | 6.95 | 0.13 | 2.44 | 7.60 | 0.64 | 3.29 | 0.12 | 11.39 |

Table S2.2: Concentrations of trace elements (mg/kg) of the stream sediments analyzed by XRF and sorted according to atomic number

| **Sample** | **S** | **V** | **Cr** | **Co** | **Ni** | **Cu** | **As** | **Rb** | **Sr** | **Zr** |
| --- | --- | --- | --- | --- | --- | --- | --- | --- | --- | --- |
| **STA01** | 550 | 67 | 39 | 6 | 20 | 19 | 61 | 369 | 371 | 258 |
| **STA02** | 630 | 96 | 53 | 11 | 33 | 54 | 45 | 276 | 419 | 211 |
| **STA03** | 150 | 70 | 37 | 7 | 19 | 14 | 56 | 371 | 405 | 230 |
| **STA04** | 580 | 99 | 59 | 12 | 31 | 23 | 51 | 281 | 415 | 270 |
| **STA05** | 120 | 145 | 99 | 25 | 42 | 34 | 69 | 227 | 318 | 168 |
| **STA06** | 310 | 145 | 98 | 16 | 44 | 38 | 29 | 244 | 307 | 173 |
| **STA07** | 170 | 191 | 138 | 21 | 54 | 44 | 24 | 202 | 253 | 198 |
| **STA08** | 760 | 96 | 61 | 14 | 38 | 27 | 30 | 210 | 463 | 171 |
| **STA09** | 1290 | 162 | 128 | 24 | 64 | 52 | 11 | 160 | 171 | 123 |
| **STA10** | 290 | 163 | 119 | 24 | 59 | 58 | 13 | 165 | 205 | 141 |
| **STA11** | 70 | 97 | 56 | 16 | 29 | 17 | 46 | 291 | 446 | 246 |
| **STA12** | 320 | 102 | 71 | 14 | 36 | 25 | 40 | 271 | 439 | 220 |
| **STA13** | 120 | 70 | 43 | 9 | 26 | 22 | 43 | 345 | 461 | 221 |
| **STA14** | 90 | 119 | 61 | 20 | 24 | 13 | 39 | 281 | 372 | 281 |
| **STA15** | 50 | 109 | 51 | 20 | 23 | 13 | 46 | 278 | 339 | 280 |
| **STA16** | 290 | 77 | 41 | 8 | 19 | 14 | 56 | 359 | 385 | 254 |
| **STA17** | 270 | 129 | 99 | 20 | 56 | 42 | 15 | 128 | 301 | 132 |
| **STA18** | 880 | 117 | 86 | 15 | 50 | 44 | 14 | 148 | 362 | 135 |
| **STA19** | 120 | 73 | 43 | 8 | 18 | 13 | 50 | 376 | 415 | 226 |
| **STA20** | 530 | 175 | 140 | 25 | 60 | 51 | 15 | 175 | 194 | 133 |
| **STA21** | 230 | 128 | 85 | 37 | 56 | 39 | 11 | 43 | 361 | 98 |
| **STA22** | 2000 | 124 | 94 | 38 | 61 | 47 | 13 | 46 | 286 | 94 |
| **STA23** | 160 | 160 | 115 | 44 | 78 | 52 | 14 | 85 | 194 | 105 |
| **STA24** | 940 | 155 | 119 | 54 | 87 | 57 | 14 | 73 | 232 | 101 |
| **STA25** | 330 | 164 | 109 | 31 | 57 | 47 | 19 | 166 | 270 | 165 |
| **STA26** | 290 | 169 | 117 | 26 | 57 | 47 | 9 | 135 | 251 | 127 |
| **STA27** | 520 | 197 | 162 | 26 | 58 | 48 | 9 | 87 | 261 | 117 |
| **STA28** | 220 | 191 | 135 | 32 | 70 | 63 | 11 | 88 | 151 | 106 |
| **STA29** | 760 | 157 | 119 | 24 | 66 | 48 | 12 | 128 | 276 | 122 |
| **STA30** | 670 | 150 | 112 | 23 | 62 | 48 | 10 | 111 | 245 | 123 |
| **STA31** | 790 | 116 | 84 | 21 | 62 | 49 | 34 | 60 | 491 | 105 |
| **STA32** | 130 | 92 | 54 | 13 | 25 | 14 | 42 | 325 | 500 | 205 |
| **STA33** | 210 | 176 | 117 | 27 | 60 | 52 | 12 | 127 | 201 | 133 |
| **STA34** | 230 | 173 | 114 | 24 | 51 | 44 | 23 | 205 | 261 | 170 |
| **STA35** | 240 | 76 | 38 | 7 | 19 | 18 | 68 | 371 | 449 | 221 |
| **STA36** | 80 | 133 | 58 | 21 | 24 | 13 | 53 | 287 | 403 | 260 |
| **STA37** | 460 | 163 | 112 | 26 | 57 | 46 | 10 | 129 | 267 | 139 |
| **STA38** | 410 | 145 | 106 | 26 | 59 | 40 | 10 | 115 | 271 | 133 |
| **STA39** | 450 | 155 | 121 | 24 | 64 | 48 | 9 | 135 | 230 | 119 |
| **STA40** | 730 | 149 | 111 | 22 | 62 | 55 | 15 | 117 | 414 | 132 |
| **STA41** | 19760 | 166 | 125 | 18 | 55 | 60 | 8 | 143 | 134 | 125 |
| **STA42** | 160 | 162 | 125 | 51 | 82 | 51 | 13 | 97 | 187 | 101 |
| **STA43** | 250 | 165 | 136 | 27 | 66 | 64 | 9 | 147 | 170 | 127 |
| **STA44** | 840 | 141 | 108 | 21 | 53 | 44 | 15 | 147 | 269 | 138 |
| **STA45** | 410 | 175 | 124 | 39 | 58 | 47 | 10 | 85 | 291 | 137 |
| **STA46** | 250 | 136 | 96 | 29 | 66 | 69 | 19 | 58 | 159 | 75 |
| **STA47** | 310 | 118 | 90 | 21 | 72 | 69 | 20 | 60 | 168 | 79 |
| **STA48** | 370 | 148 | 113 | 23 | 74 | 69 | 9 | 79 | 234 | 104 |
| **STA49** | 430 | 190 | 141 | 26 | 65 | 56 | 10 | 85 | 161 | 121 |
| **STA50** | 190 | 234 | 172 | 36 | 75 | 67 | 11 | 93 | 140 | 127 |
| **STA51** | 3590 | 112 | 85 | 42 | 86 | 66 | 49 | 47 | 206 | 74 |
| **STA52** | 630 | 166 | 109 | 24 | 53 | 46 | 8 | 68 | 337 | 125 |
| **STA53** | 1900 | 104 | 83 | 20 | 48 | 40 | 69 | 47 | 321 | 85 |
| **STA54** | 260 | 206 | 156 | 36 | 86 | 71 | 10 | 101 | 120 | 122 |
| **9080A22** | 380 | 163 | 121 | 32 | 69 | 51 | 10 | 116 | 228 | 121 |
| **9081A22** | 680 | 123 | 91 | 26 | 56 | 35 | 8 | 78 | 279 | 108 |
| **9083A22** | 360 | 165 | 120 | 39 | 63 | 50 | 10 | 89 | 248 | 106 |
| **9163A22** | 290 | 168 | 121 | 33 | 69 | 59 | 8 | 100 | 201 | 126 |
| **9171A22** | 1230 | 88 | 73 | 14 | 47 | 40 | 54 | 46 | 326 | 82 |
| **9211A22** | 170 | 183 | 155 | 49 | 92 | 55 | 14 | 63 | 193 | 93 |
| **GIT04** | 4760 | 86 | 56 | 44 | 62 | 44 | 48 | 152 | 278 | 182 |
| **GIT12** | 1330 | 150 | 131 | 25 | 76 | 56 | 21 | 169 | 205 | 184 |
| **Green Lake** | 640 | 92 | 59 | 9 | 25 | 21 | 44 | 347 | 436 | 256 |
| **MSIE11** | 290 | 206 | 145 | 35 | 65 | 56 | 10 | 77 | 261 | 118 |
| **MSIE16** | 640 | 221 | 168 | 25 | 60 | 52 | 8 | 89 | 261 | 131 |
| **MSIE05** | 100 | 119 | 85 | 17 | 48 | 44 | 5 | 53 | 437 | 120 |
| **PA-1** | 590 | 162 | 134 | 20 | 64 | 61 | 8 | 151 | 189 | 139 |
| **SALTO1** | 4740 | 143 | 104 | 17 | 50 | 36 | 9 | 58 | 338 | 133 |
| **SALTO2** | 750 | 191 | 117 | 26 | 57 | 46 | 7 | 70 | 332 | 158 |
| **SALTO3** | 6990 | 145 | 103 | 17 | 50 | 47 | 13 | 69 | 132 | 138 |
| **SALTO4** | 1320 | 143 | 102 | 15 | 47 | 46 | 14 | 71 | 227 | 139 |
| **SIE BR2** | 820 | 179 | 138 | 25 | 67 | 59 | 9 | 91 | 297 | 125 |
| **SIE189** | 610 | 159 | 128 | 23 | 67 | 58 | 7 | 127 | 221 | 125 |

Table S2.3: Concentration of pH, As, Sb Co, Cr, Cu, Ni, and V (mg/kg) analyzed by ICP-AES, Hg (mg/kg) by method EPA7473 and Organic Matter (OM) expressed as wt. % in stream sediments. n.d.= no detected.

| **Sample** | **pH** | **As_ICP** | **Hg** | **Sb_ICP** | **Co_ICP** | **Cr_ICP** | **Cu_ICP** | **Ni_ICP** | **V_ICP** | **OM** |
| --- | --- | --- | --- | --- | --- | --- | --- | --- | --- | --- |
| **STA1** | 8.17 | 19 | 1.9 | <1 | 3.5 | 10.2 | 8.6 | 6.6 | 17.4 | 7.6 |
| **STA2** | 7.75 | 14 | 3.4 | 2 | 6.1 | 15.6 | 39.0 | 16.3 | 21.1 | 4.7 |
| **STA3** | 8.23 | 21 | 2.4 | <1 | 4.9 | 16.0 | 3.8 | 6.8 | 28.6 | 2.9 |
| **STA4** | 7.94 | 18 | 22.3 | 1 | 3.8 | 9.1 | 5.9 | 8.0 | 14.6 | 5.1 |
| **STA5** | 8.01 | 49 | 17.3 | 5 | 13.5 | 24.3 | 15.9 | 20.4 | 33.5 | 5.6 |
| **STA6** | 7.94 | 6 | 0.5 | 3 | 7.9 | 17.1 | 15.9 | 16.1 | 19.7 | 3.5 |
| **STA7** | 8.2 | 10 | 0.6 | 6 | 18.4 | 44.1 | 33.9 | 36.1 | 50.5 | 6.7 |
| **STA8** | 8.29 | 11 | 64.6 | 2 | 4.2 | 7.6 | 6.4 | 9.1 | 12.3 | 3 |
| **STA9** | 7.81 | 4 | 0.8 | 2 | 4.6 | 11.0 | 14.4 | 14.0 | 7.5 | 4.2 |
| **STA10** | 8.25 | 5 | 0.4 | 8 | 13.9 | 28.6 | 39.6 | 35.2 | 23.1 | 2.5 |
| **STA11** | 8.04 | 11 | 0.1 | <1 | 3.3 | 8.3 | 3.5 | 6.4 | 15.2 | 2.4 |
| **STA12** | 7.48 | 16 | 1.1 | 1 | 3.5 | 7.0 | 5.8 | 6.9 | 12.5 | 4.4 |
| **STA13** | 7.44 | 18 | 0.3 | <1 | 2.6 | 5.7 | 3.4 | 4.0 | 11.3 | 7.3 |
| **STA14** | 7.18 | 6 | 1.4 | <1 | 4.4 | 14.3 | 3.6 | 6.5 | 25.2 | 2.2 |
| **STA15** | 7 | 6 | 0.4 | <1 | 4.6 | 15.0 | 3.5 | 6.7 | 26.3 | 2.3 |
| **STA16** | 7.33 | 23 | 4.9 | <1 | 4.3 | 15.1 | 3.5 | 6.1 | 26.2 | 3.9 |
| **STA17** | 8.13 | 3 | 0.2 | 3 | 9.9 | 19.7 | 21.0 | 24.2 | 16.0 | 4.7 |
| **STA18** | 8.12 | 4 | 0.7 | 4 | 8.6 | 19.2 | 25.9 | 23.3 | 17.0 | 7.4 |
| **STA19** | 6.54 | 7 | 0.85 | <1 | 2.6 | 7.2 | 2.9 | 4.4 | 12.6 | 3.8 |
| **STA20** | 7.02 | 7 | 3.32 | 6 | 10.1 | 23.1 | 31.2 | 26.1 | 15.9 | 4.4 |
| **STA21** | 7.64 | 3 | 29.4 | 4 | 13.7 | 13.8 | 14.4 | 21.3 | 19.9 | 6.5 |
| **STA22** | 7.82 | 8 | 487 | 9 | 16.2 | 16.9 | 24.6 | 35.0 | 24.4 | 3.9 |
| **STA23** | 6.75 | 7 | 14.5 | 5 | 18.2 | 18.8 | 27.0 | 36.6 | 21.7 | 6.1 |
| **STA24** | 7.86 | 7 | 8.7 | 4 | 16.4 | 14.1 | 23.7 | 33.7 | 17.2 | 5.2 |
| **STA25** | 7.65 | 8 | 1.31 | 3 | 10.8 | 15.4 | 18.0 | 18.8 | 15.4 | 6.5 |
| **STA26** | 7.95 | 3 | 0.501 | 4 | 10.1 | 19.0 | 20.8 | 22.3 | 15.2 | 7.7 |
| **STA27** | 7.68 | 4 | 0.209 | 3 | 9.7 | 21.2 | 19.0 | 20.0 | 22.9 | 6.3 |
| **STA28** | 7.15 | 7 | 0.143 | 4 | 14.6 | 26.3 | 32.2 | 29.5 | 28.8 | 6.2 |
| **STA29** | 7.66 | 7 | 0.9 | 5 | 13.6 | 28.4 | 35.7 | 37.4 | 19.9 | 3.3 |
| **STA30** | 7.36 | 3 | 6.52 | 4 | 10.8 | 21.8 | 29.2 | 28.8 | 18.5 | 4.1 |
| **STA31** | 7.85 | 24 | 1.12 | 4 | 12.7 | 14.8 | 29.2 | 28.6 | 19.4 | 14.7 |
| **STA32** | 7.46 | 4 | 0.242 | <1 | 2.1 | 5.5 | 2.7 | 3.9 | 10.2 | 1.6 |
| **STA33** | 7.59 | 3 | 0.92 | 5 | 12.2 | 24.0 | 27.2 | 28.1 | 23.0 | 5.6 |
| **STA34** | 7.82 | 5 | 0.491 | 4 | 11.1 | 24.4 | 22.9 | 23.4 | 26.5 | 5 |
| **STA35** | 7.9 | 23 | 0.381 | 2 | 2.8 | 7.0 | 4.2 | 4.9 | 12.5 | 2.6 |
| **STA36** | 8.97 | 19 | 0.325 | <1 | 4.5 | 14.8 | 3.5 | 6.6 | 27.0 | 1.5 |
| **STA37** | 8.14 | 5 | 0.189 | 6 | 18.5 | 26.4 | 36.8 | 42.2 | 21.3 | 4.7 |
| **STA38** | 7.78 | 3 | 0.236 | 4 | 11.6 | 19.1 | 20.8 | 28.4 | 15.1 | 4.2 |
| **STA39** | 7.5 | 2 | 0.117 | 3 | 9.1 | 16.6 | 21.3 | 23.5 | 12.0 | 28.5 |
| **STA40** | 8.23 | 8 | 1.98 | 4 | 10.3 | 19.8 | 26.3 | 27.0 | 15.4 | 5.9 |
| **STA41** | 4.17 | 3 | 0.088 | 6 | 11.9 | 33.6 | 52.0 | 37.7 | 23.6 | 7.2 |
| **STA42** | 8.7 | 3 | 0.474 | 8 | 22.6 | 24.1 | 32.7 | 50.3 | 25.1 | 22 |
| **STA43** | 7.97 | 2 | 0.104 | 4 | 11.7 | 23.1 | 38.5 | 32.0 | 15.9 | 4.5 |
| **STA44** | 7.46 | 5 | 27.7 | 8 | 11.2 | 24.1 | 36.5 | 30.0 | 17.2 | 3.2 |
| **STA45** | 7.93 | 4 | 2.1 | 8 | 29.2 | 33.4 | 34.2 | 39.8 | 41.3 | 6.5 |
| **STA46** | 7.85 | 5 | 209 | 13 | 8.7 | 10.9 | 18.4 | 15.7 | 15.0 | 3.6 |
| **STA47** | 7.92 | 7 | 740 | 4 | 8.5 | 11.3 | 22.7 | 19.0 | 15.2 | 4.2 |
| **STA48** | 7.31 | 2 | 0.72 | 5 | 14.8 | 28.2 | 38.1 | 32.8 | 33.6 | 19.7 |
| **STA49** | 7.26 | 3 | 7.3 | 5 | 13.2 | 33.6 | 28.9 | 27.9 | 37.6 | 8.2 |
| **STA50** | 7.05 | 4 | 68 | 5 | 19.0 | 40.6 | 38.7 | 35.6 | 44.5 | 5.9 |
| **STA51** | 7.67 | 51 | 1.95 | 48 | 26.6 | 18.4 | 44.6 | 51.4 | 26.4 | 4.4 |
| **STA52** | 7.3 | 2 | 72 | 4 | 8.7 | 10.9 | 22.8 | 19.0 | 17.2 | 5.7 |
| **STA53** | 7.49 | 30 | 379 | 84 | 6.6 | 9.1 | 14.4 | 14.7 | 12.6 | 4.4 |
| **STA54** | 7.12 | 3 | 7.3 | 6 | 22.7 | 45.7 | 51.1 | 47.7 | 52.2 | 7.8 |
| **MSIE-16** | 7.7 | 4 | 8.4 | 6 | 17.6 | 44.9 | 52 | 36.6 | 44.2 | 7.7 |
| **MSIE-04** | 7.7 | 4 | 31 | 10 | 23.8 | 34.8 | 54.5 | 54 | 41.4 | 7.7 |
| **sie 189** | 7.73 | 3 | 31 | 8 | 16.1 | 31.4 | 43.5 | 43.7 | 24.3 | 9.3 |
| **SIE Br2** | 7.76 | 3 | 11.1 | 7 | 17.6 | 33.7 | 44.9 | 45.5 | 38.7 | 6.8 |
| **MSIE 05** | 7.61 | 2 | 5 | 5 | 13 | 25.3 | 33.4 | 29.9 | 32.5 | 15.7 |
| **Msie 11** | 7.66 | 4 | 16.6 | 9 | 22.6 | 40.6 | 56.6 | 50.2 | 54.1 | 6.5 |
| **PA01** | n.d. | 2 | 850 | 6 | 15.6 | 32.4 | 64.6 | 42 | 21.4 | 5.1 |
| **GIT0** | n.d. | 311 | 62.9 | <1 | 10.3 | 11.5 | 12.6 | 14.7 | 69 | n.d. |
| **GIT12** | n.d. | 7 | 123 | 6 | 15.1 | 28 | 53 | 42.4 | 17.9 | 5.1 |
| **GIT4** | n.d. | 21 | 114 | 5 | 12.4 | 11.3 | 20.7 | 26.8 | 19.5 | 12.2 |
| **Green lake** | 6.29 | 8.9 | 0.6 | 1 | 3.6 | 10.1 | 5.4 | 7 | 16.8 | 3.5 |
| **Salto 1** | 7.56 | 3.9 | 31 | 5.9 | 13.1 | 23 | 26.7 | 29.1 | 32.5 | 5.5 |
| **Salto 2** | 7.53 | 3 | 0.8 | 5.9 | 16 | 27.5 | 30.8 | 32.6 | 44 | 7.2 |
| **Salto 3** | 7.57 | 10.8 | 22 | 4.9 | 11 | 25.4 | 27.1 | 26.6 | 32.4 | 2.3 |
| **Salto 4** | 7.41 | 7.9 | 3.7 | 5.9 | 11.5 | 29.1 | 29.1 | 28.6 | 35.7 | 4.5 |
| **9163A22** | 7.78 | 5.8 | 3.4 | <1 | 24.4 | 62.7 | 76.1 | 77.3 | 82.1 | 10.1 |
| **9171A22** | 7.74 | 84 | 153 | <1 | 15.2 | 31.9 | 58 | 48.2 | 49.6 | 3.7 |
| **9080A22** | 7.75 | 4.3 | 105 | <1 | 46.7 | 66.4 | 60.5 | 74.5 | 75 | 4.9 |
| **9081A22** | 7.82 | 3.7 | 0.8 | <1 | 27.4 | 43 | 47.3 | 52 | 59.7 | 3.7 |
| **9083A22** | 7.87 | 5.4 | **Hg** | <1 | 24.1 | 53.7 | 61.9 | 71.6 | 74.5 | 6.6 |
| **9211A22** | 7.6 | 9.1 | 1.9 | <1 | 38.4 | 80 | 84.2 | 95.9 | 99.2 | 5.7 |
